# Efficacy and safety of anti-androgens in the management of polycystic ovary syndrome: a systematic review and meta-analysis of randomised controlled trials

**DOI:** 10.1016/j.eclinm.2023.102162

**Published:** 2023-08-09

**Authors:** Simon Alesi, Maria Forslund, Johanna Melin, Daniela Romualdi, Alexia Peña, Chau Thien Tay, Selma Feldman Witchel, Helena Teede, Aya Mousa

**Affiliations:** a Monash Centre for Health Research and Implementation (MCHRI), Monash University, Clayton, VIC, Australia; b Department of Obstetrics and Gynecology, Institute of Clinical Sciences, Sahlgrenska Academy, University of Gothenburg, Gothenburg, Sweden; c Department of Obstetrics and Gynecology, University of Helsinki, Helsinki University Hospital, Helsinki, Finland; d Division of Paediatric Endocrinology, UPMC Children's Hospital of Pittsburgh, University of Pittsburgh, Pittsburgh, PA, USA; e Fondazione Policlinico Universitario Agostino Gemelli, Rome, Italy; f Discipline of Paediatrics, The University of Adelaide and Robinson Research Institute, Adelaide, Australia

**Keywords:** Anti-androgens, Combined oral contraceptives, Guideline, Meta-Analyses, Systematic review, Polycystic ovary syndrome (PCOS), Spironolactone

## Abstract

**Background:**

Anti-androgens and combined oral contraceptive pills (COCPs) may mitigate hyperandrogenism-related symptoms of polycystic ovary syndrome (PCOS). However, their efficacy and safety in PCOS remain unclear as previous reviews have focused on non-PCOS populations. To inform the 2023 International Evidence-based Guideline in PCOS, we conducted the first systematic review and meta-analysis investigating the efficacy and safety of anti-androgens in the management of hormonal and clinical features of PCOS.

**Methods:**

We systematically searched MEDLINE, Embase, PsycInfo, All EBM reviews, and CINAHL up to 28th June 2023 for randomised controlled trials (RCTs) examining oral anti-androgen use, alone or in combination with metformin, COCPs, lifestyle, or other interventions, in women of any age, with PCOS diagnosed by Rotterdam, National Institutes of Health or Androgen Excess & PCOS Society criteria, and using a form of contraception. Non-English studies and studies of less than 6 months duration or which used the same anti-androgen regimen in both/all groups were excluded in order to establish efficacy for the clinical outcomes of interest. Three authors screened articles against selection criteria and assessed risk of bias and quality using the Grading of Recommendations, Assessment, Development and Evaluations (GRADE) framework. Critical outcomes (prioritised during guideline development for GRADE purposes) included weight, body mass index (BMI), irregular cycles, hirsutism, liver function, and quality of life. Random effects meta-analyses were conducted where appropriate. This study is registered with PROSPERO, CRD42022345640.

**Findings:**

From 1660 studies identified in the search, 27 articles comprising 20 unique studies were included. Of these, 13 studies (n = 961) were pooled in meta-analysis. Seven studies had a high risk of bias, nine moderate and four low. Anti-androgens included finasteride, flutamide, spironolactone, or bicalutamide. In meta-analysis, anti-androgens + lifestyle were superior to metformin + lifestyle for hirsutism (weighted mean difference [WMD] [95% CI]: −1.59 [−3.06, −0.12], p = 0.03; *I*^*2*^ = 74%), SHBG (7.70 nmol/l [0.75, 14.66], p = 0.03; *I*^*2*^ = 0%), fasting insulin and fasting insulin: glucose ratio (−2.11 μU/ml [−3.97, −0.26], p = 0.03; *I*^*2*^ = 0% and −1.12 [−1.44, −0.79], p < 0.0001, *I*^*2*^ = 0%, respectively), but were not superior to placebo + lifestyle for hirsutism (−0.93, [−3.37, 1.51], p = 0.45; *I*^*2*^ = 76%) or SHBG (9.72 nmol/l [−0.71, 20.14], p = 0.07; *I*^*2*^ = 31%). Daily use was more effective for hirsutism than use every three days (−3.48 [−4.58, −2.39], p < 0.0001, *I*^*2*^ = 1%), and resulted in lower androstenedione levels (−0.30 ng/ml [−0.50, −0.10], p = 0.004; *I*^*2*^ = 0%). Combination treatment with anti-androgens + metformin + lifestyle resulted in lower testosterone compared with metformin + lifestyle (−0.29 nmol/l [−0.52, −0.06], p = 0.01; *I*^*2*^ = 61%), but there were no differences in hirsutism when anti-androgens + metformin + lifestyle were compared with either anti-androgens + lifestyle or metformin + lifestyle. In limited meta-analyses (n = 2 trials), combining anti-androgens with COCP resulted in poorer lipid profiles compared with COCP ± placebo, with no differences in other outcomes.

**Interpretation:**

Current evidence does not support the use of anti-androgens preferentially to COCPs to treat hyperandrogenism in PCOS. Anti-androgens could be considered to treat hirsutism in PCOS, where COCPs are contraindicated, poorly tolerated, or present a sub-optimal response after a minimum 6-month period, with consideration of clinical context and individual risk factors and characteristics.

**Funding:**

10.13039/501100000925National Health and Medical Research Council (NHMRC) of Australia Monash University.

**Research in context**

**Evidence before this study**

The 2018 International Evidence-based Guideline in Polycystic Ovary Syndrome (PCOS) identified no systematic reviews of randomised controlled trials (RCTs) examining the use of anti-androgens in PCOS. To identify studies and systematic reviews published since the 2018 guideline, we searched electronic databases including MEDLINE, Embase, PsycInfo, All EBM reviews, and CINAHL from 2017 to 28th June 2023. We included search terms related to: polycystic ovary syndrome OR oligo-ovulation OR anovulation OR hyperandrogenism AND combined oral contraceptive pills OR metformin OR androgen antagonists OR spironolactone OR finasteride OR cyproterone acetate or flutamide OR anti-obesity OR orlistat OR sibutramine OR inositol AND meta-analysis OR review OR clinical trial OR random. We identified nine newly published articles, but found no systematic reviews or meta-analyses of RCTs investigating the use of anti-androgens in PCOS.

**Added value of this study**

We conducted the first systematic review of RCTs investigating the efficacy and safety of anti-androgen pharmacological agents in PCOS. The study used a comprehensive search, including both adult and adolescent populations and using internationally endorsed guideline methodology for identifying, evaluating, and appraising the literature, including the Grading of Recommendations Assessment Development and Evaluations (GRADE) tool to inform guideline recommendations. Combined with studies from the previous 2018 guideline, a total of 27 articles reporting on 20 unique RCTs were included. We assessed a wide range of outcomes relevant to PCOS as determined by experts and consumer groups in the guideline development process, thereby providing broad insights into the efficacy of anti-androgens, with or without other interventions, in managing key features of PCOS. Our findings showed that anti-androgens may have possible benefits for treating hirsutism, in circumstances where combined oral contraceptive pills (COCPs) are contraindicated, poorly tolerated, or have been ineffective for over six months, with consideration of clinical context and individual needs and risk factors.

**Implications of all the available evidence**

This study has directly informed the recommendations of the 2023 International Evidence-based Guideline for the Assessment and Management of PCOS, endorsed by 39 organisations globally. Our findings have addressed key gaps in the literature and provide evidence to guide clinical practice. However, while these recommendations are based on the best available evidence, this is largely of low certainty and differences in the efficacy and side effects of different anti-androgens and their effectiveness across various PCOS features, ages and anthropometric characteristics could not be accurately determined. Further high-quality RCTs in this area are encouraged.

## Introduction

Polycystic ovary syndrome (PCOS) is the most common endocrinological disorder in women of reproductive age, with a prevalence of 8–13%.^1^ According to the Rotterdam criteria, PCOS is diagnosed based on the presence of two out of three characteristics: clinical or biochemical hyperandrogenism, menstrual irregularities (oligo or anovulation), and the detection of polycystic ovary morphology on ultrasound, after the exclusion of other causes.^2^ The Androgen Excess-PCOS Society (AE-PCOS) and National Institutes of Health (NIH) state that PCOS should be defined by the presence of hyperandrogenism, ovarian dysfunction, and the exclusion of other related disorders.^3^ While the AE-PCOS and NIH largely agree with the diagnostic criteria outlined in Rotterdam, hyperandrogenism is considered to be a fundamental component in the pathophysiology of PCOS.^4^^,^^5^ Reproductive (irregular menses, infertility and pregnancy complications),^6^ metabolic (increased prevalence of obesity, type 2 diabetes, and cardiovascular disease), and psychological (depression, disordered eating, body image distress, and reduced quality of life) characteristics are associated with PCOS.^7^^,^^8^

Lifestyle management is recommended as the first-line treatment option for PCOS. However, in some circumstances where lifestyle management is unsuccessful, pharmacological agents including the combined oral contraceptive pill (COCP), anti-obesity agents, metformin, and anti-androgen medications, could be employed for clinical management.^9^ The COCP is often prescribed for adult women with PCOS, for management of irregular menses and clinical hyperandrogenism (hirsutism and acne).^10^ Similarly, COCPs are recommended for adolescent girls diagnosed with PCOS and those considered to be “at risk for PCOS”. Metformin has been used for ovulation induction, promotion of weight loss and/or maintenance, and reduction of pregnancy complications.^11^, ^12^, ^13^

Anti-androgen medications such as spironolactone, flutamide, finasteride, and cyproterone acetate (CPA) have been utilised to decrease hyperandrogenism-related symptoms. Anti-androgen medications act in one of three ways; either by competitively inhibiting the androgen-binding receptors; decreasing androgen production (although the manner in which this occurs is not well-understood); or inhibiting 5-α-reductase in the skin, which is an enzyme that converts testosterone into its active form, 5-α-dihydrotestosterone (DHT).^14^ Spironolactone and finasteride are dose-dependent competitive inhibitors of the androgen receptor and have also been observed to inhibit 5-α-reductase activity.^15^ Other anti-androgens such as CPA, flutamide, and bicalutamide are thought to competitively inhibit testosterone binding to the androgen receptor.^16^ Via these mechanisms, anti-androgen medications could ameliorate the hyperandrogenic state of PCOS and improve the various hyperandrogenism-related symptoms associated with the condition.

While anti-androgens and their mechanisms of action allude to plausible benefits, the 2018 PCOS guidelines^9^ concluded that evidence was lacking for the use of all identified anti-androgen medications in PCOS. Existing systematic reviews, including a network meta-analysis,^17^ have focused on women with idiopathic hirsutism^17^ or broader hirsute populations^18^ and, to our knowledge, no systematic reviews have examined the use of anti-androgens in the management of PCOS. In response to this identified gap, recent published studies have appealed for re-examination of the potential benefits of anti-androgens in PCOS. Therefore, as part of the updated 2023 International Evidence-based Guidelines in PCOS, this systematic review and meta-analysis aimed to investigate the efficacy and safety of anti-androgens, alone or in combination, on anthropometric, hormonal and clinical features of PCOS.

## Methods

### Search strategy and selection criteria

This systematic review is reported in accordance with the Preferred Reporting Items for Systematic Reviews and Meta-Analyses (PRISMA) guideline,^19^ and was developed to inform clinical practice recommendations in the update of the International Evidence-based Guideline for the Assessment and Management of PCOS. This review was part of a larger evidence synthesis covering several pharmacological treatments including COCP, metformin, and anti-androgens and, although each intervention review is reported separately (due to different eligibility criteria), the combined protocol was registered *a priori* on the international prospective register of systematic reviews (PROSPERO): CRD42022345640. The methodology utilised in this systematic review is endorsed by the National Health and Medical Research Council (NHMRC) of Australia^20^ and the European Society of Human Reproduction and Embryology (ESHRE)^21^ and has been previously described in detail in the 2018 guidelines^9^ and in the publicly available Technical Report.^22^

The present systematic review addresses the following clinical question:

*Are anti-androgen pharmacological agents, alone or in combination, effective for management of hormonal and clinical PCOS features and weight in adolescents and adults with PCOS?*

The Participant-Intervention-Comparison-Outcome (PICO) framework was adopted, with selection criteria developed *a priori* by the multi-disciplinary guideline development group, including consumers. These eligibility criteria are outlined in Table 1. The search strategy, including database selection and search terms (Medical Subject Headings (MeSH) and keywords), was developed in consultation with technical and content experts, clinicians with expertise in PCOS management, and a skilled medical librarian. As this was part of a larger evidence synthesis, a variety of keywords relating to PCOS, anti-androgens, metformin, COCP, and relevant outcomes, were used in the search strategy (Supplementary Material). The following databases were searched: MEDLINE, EMBASE, APA PsycInfo, all evidence-based medicine (EBM) reviews (all via Ovid processing), and CINAHL (via EBSCO). The previous 2018 International Evidence-based Guidelines in PCOS searched from inception to 11th January 2017.^9^ For the current systematic review, we incorporated the studies identified in the 2018 guideline and reran the search from 2017 to 8th July 2022, with a further updated search on 28th June 2023.

**Table 1:** Eligibility criteria (PICO) for study inclusion.

	Participants (P)	Intervention (I)	Comparison (C)	Outcomes (O)
Inclusion	Females with PCOS (diagnosed by Rotterdam, NIH, or AES) of any age, ethnicity, and weight. Subgroups: adolescents (10–19 y), adults, post-menopausal. Contraception must be reported.	Oral anti-androgen pharmacological agents (SPL, CPA finasteride, flutamide, bicalutamide) alone or in combination with lifestyle, metformin, OCP/COCP, anti-obesity agents. All doses, duration of more than 6 months.	Placebo or any other intervention (listed in intervention) or combinations of those listed in intervention.	Several outcomes were included, relating to hormonal (FAI, testosterone, SHBG, DHEAS, androstenedione, hirsutism by FG scores, and irregular cycles); anthropometric (weight, BMI, WHR); and metabolic parameters (total, HDL, or LDL] cholesterol, triglycerides, insulin resistance/glucose metabolism by HOMA, hyperinsulinemic-euglycemic clamps, or OGTT, and CRP). Psychological outcomes and adverse effects were also extracted, which included QoL, depression, mood, liver function tests (transaminases), and pregnancy complications.
Exclusion	Females without PCOS.		Agent or combination used in the intervention (i.e., CPA as part of COCP).	

**Abbreviations: AES**, Androgen Excess Society; **BMI**, body mass index; **CPA**, cyproterone acetate; **CRP**, C-reactive protein; **DHEAS**, dehydroepiandrosterone sulphate; **FAI**, free androgen index; **FG**, Ferriman-Gallwey; **HDL**, high density lipoprotein; **HOMA**, homeostatic model assessments; **LDL**, low density lipoprotein; **NIH**, National Institute of Health; **OCP/COCP**, oral contraceptive pill/combined oral contraceptive pill; **OGTT**, oral glucose tolerance test; **PCOS**, polycystic ovary syndrome; **QoL**, quality of life; **SHBG**; sex-hormone binding globulin; **SPL**, spironolactone; **WHR**, waist-to-hip ratio.

Consistent with the previous guideline,^9^ the search included only randomised controlled trial (RCT) study designs (or systematic reviews of RCTs to identify eligible studies) and was restricted to English language studies. When incorporating studies from the previous guideline, we ensured that the current inclusion criteria were met. These criteria differ slightly from the 2018 criteria, in that we are no longer excluding RCTs which either did not utilise a robust method of randomisation or did not describe the method clearly in the manuscript. In the current update, we are considering randomisation methods/reporting in the risk of bias assessments, rather than excluding the studies completely on this basis.

Screening was undertaken using Covidence (www.covidence.org). Title and abstract screening and full text screening were conducted in duplicate by two of three independent reviewers (SA, MF, and JM). Risk of bias (RoB) was assessed for each study in duplicate by two of three independent reviewers (SA, MF, and JM), using criteria developed *a priori.* For studies included in the 2018 guideline, the RoB assessments were derived from the previous review by the 2018 guideline evidence team.^9^ Individual quality items included selection bias, performance bias, detection bias, attrition bias, reporting bias, as well as other bias (such as conflicts of interest and accuracy of statistical analyses). Any disagreement or uncertainty was resolved by discussion among the reviewers to reach consensus or referred to the guideline evidence team if required. Each study was allocated a rating of low, moderate, or high RoB (Supplementary Material). In circumstances where multiple studies arise from a single patient population, the main study reporting the primary outcome(s) (which often also had the largest sample size and complete outcome set) was assessed for RoB. All smaller sub-studies were denoted with the same RoB ranking allocated to the main study.

### Data analysis

Data were extracted by one reviewer (SA), with independent cross-checking by two reviewers (MF, JM) to ensure accuracy. A template was developed for data extraction of relevant items, including study details (author, year of publication, country of origin, study design, sample size), participants (population characteristics, setting, and method of PCOS diagnosis), intervention and comparison (type, dose, duration, frequency, and combinations) and relevant outcomes, including units and assessment methods where necessary.

Meta-analyses were performed using Review manager V.5.4. Due to clinical heterogeneity from differences in doses, intervention timing, and outcome measures, random effects models were used for all meta-analyses. Effect estimates are presented as the weighted mean difference (WMD) for continuous outcomes, and the odds ratio (OR) for dichotomous outcomes, with 95% confidence intervals (CIs). Results are presented in summary tables and forest plots. Where only a single study reported an outcome or comparison, this was included as a single-paper meta-analysis, to facilitate the communication and comparison of results across studies, thus simplifying assessments of convergence while providing more precise estimates of effects and the uncertainty surrounding these estimates.^23^ Where data for a given outcome were reported in a main study and again in a sub-study of the same patient population, only one study was included in the relevant meta-analysis, usually the main study with the larger sample size. Where data were not reported or were reported only as median (and interquartile ranges) or changes from baseline, this precluded meta-analysis and these studies were instead presented narratively in a descriptive analysis.

Subgroup analyses were planned *a priori* to stratify studies by age, ethnicity or phenotype, wherever possible. Sensitivity analyses by RoB were conducted to examine the impact of high risk studies on results. Here, high RoB studies are excluded from each meta-analysis to establish their influence on results, and increase confidence that the observed effects (or lack thereof) are unlikely to be the product of biased data. Statistical heterogeneity was determined by an *I*^*2*^, whereby *I*^*2*^ > 50% is considered high, urging caution in the interpretation of those results. Publication bias was assessed by visual inspection of funnel plot asymmetry to detect small study effects.

Using the Grading of Recommendations Assessment, Development and Evaluation (GRADE) framework, outcomes were prioritised as either critical or important after careful deliberation among the clinical leads of the guideline development group for this review question.^24^^,^^25^ Critical outcomes included weight, body mass index (BMI), irregular cycles, hirsutism, liver function, and quality of life (QoL). Detailed appraisals of GRADE components are presented in the Supplementary Material, which include the RoB assessments, inconsistency (e.g. different directions of effect, confidence intervals not overlapping, and statistical heterogeneity), indirectness (e.g. important variations in populations or interventions), imprecision (small sample sizes and wide confidence intervals) or other biases (e.g. study design issues). The GRADE assessments were conducted in duplicate by two of three reviewers (SA, MF and JM).

### Role of the funding source

The funder of the study had no role in study design, data collection, data analysis, data interpretation, or writing of the report. SA, MF, JM and AM had access to the dataset and all authors (SA, AM, JM, DR, AP, CTT, SFW, HT and AM) had final responsibility for the decision to submit for publication.

## Results

**Fig. 1:**
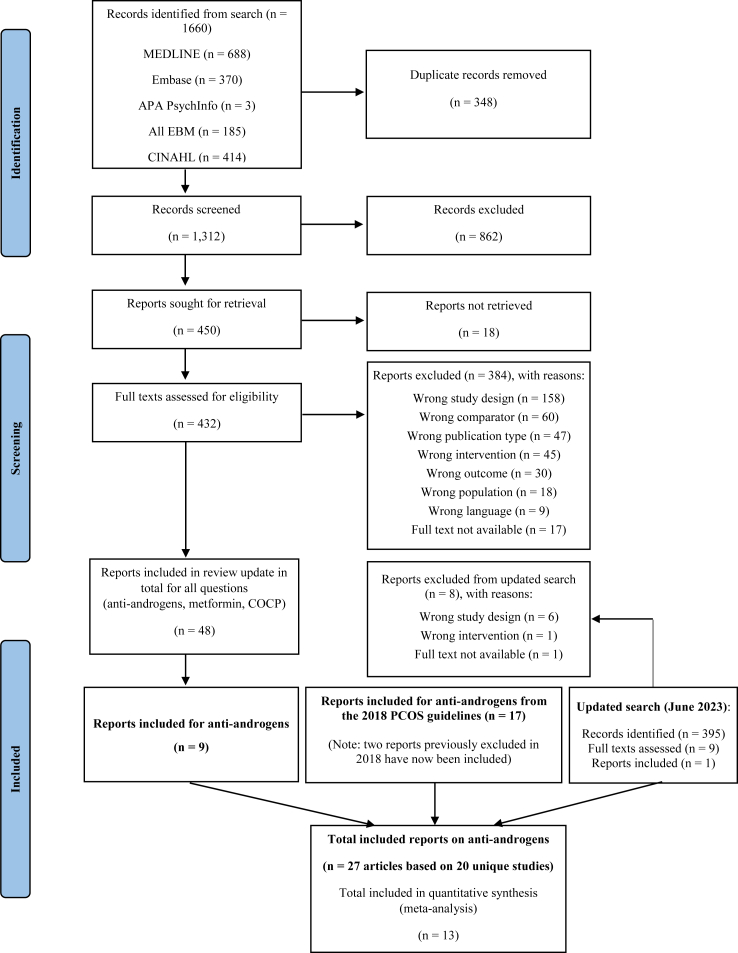
PRISMA diagram for literature search process and total number of studies screened and included at each stage. PCOS, polycystic ovary syndrome.

After screening 1312 titles and abstracts and 432 full texts, 48 articles were identified from the combined search for anti-androgens, metformin, and COCP, of which nine pertained to anti-androgens specifically and were included in the present review (Fig. 1). In addition to these nine newly identified articles, 17 studies of anti-androgens were included from the previous 2018 guideline (two of which were previously excluded but met current criteria). An updated search was also conducted in June 2023, where 395 titles and abstracts and nine full texts were screened. From these, one additional eligible study was identified, bringing the total to 27 articles corresponding to 20 unique studies. Of these, 13 could be pooled in meta-analysis. The full text articles excluded with reasons for exclusion are listed in the Supplementary Material.

### Characteristics and quality of included trials

Characteristics of the 27 included articles comprising the 20 unique studies are presented in Table 2, and a summary of results from meta-analyses of key outcomes is provided in Table 3. Additional detailed results are provided in the Supplementary Material. Although planned, subgroup analyses were not possible due to the small number and high heterogeneity of the included studies.

**Table 2:** Key characteristics of studies included in a systematic review and meta-analysis of anti-androgen use in polycystic ovary syndrome.

Author, year, country	Population/Setting	Intervention N	Intervention description	Comparison N	Comparison description	Follow Up	Outcomes	Type of contraception	Risk of bias
Alpañés 2017, Spain	Women with PCOS/Androgen excess outpatient clinic	1: 18	**1:** 30 μg EE+ 150 μg DG + 100 mg spironolactone	2: 13	**2:** Metformin 850 mg b.i.d.	12 months	Frequency of menstrual dysfunction hirsutism score, BMI, waist circumference, serum total and free testosterone, androstenedione and DHEAS, OGTT, serum insulin and plasma glucose, HOMA, adverse effects	Non-hormonal contraception	High
Amiri 2014, Iran	Overweight and obese infertile women with PCOS/Fatemezahra Infertility and Reproductive Health Centre	1: 27 2: 27	**1:** 250 mg FLU 2/day +1 month HC pre-diet **2:** 500 mg MET 3/day +250 mg FLU 2/day +1 month HC pre-diet	3: 25 4: 26	**3:** 500 mg MET 3/day + 1-month HC pre-diet **4.** PLAC	6 months	BMI, WHR, Hirsutism, SHBG, Testosterone, DHEAS, fasting insulin, fasting glucose, OGTT, QUICKI, Total cholesterol, HDL, LDL, Triglycerides	Barrier contraception	High
Diri 2017, Turkey	Patients with PCOS/Department of Endocrinology Erciyes University Medical School	1: 16 2: 19	**1:** FIN 5 mg/day **2:** FIN 5 mg/day + MET	3: 17	**3:** MET 1700 mg/day	12 months	BMI, Hirsutism, SHBG, Free testosterone, DHEAS, Androstenedione, HOMA-IR	Barrier contraception	High
Dumesic 2023, USA	Normal weight women with PCOS/An academic medical centre	1: 5	1: 125 mg/day FLU	2: 6	**2:** Placebo	6 months	BMI, body weight, total testosterone, free testosterone, androstenedione, dihydrotestosterone, DHEAS, fasting glucose, fasting insulin, insulin sensitivity, SHBG, total cholesterol	Abstinence or nonhormonal form of contraception	Moderate
Falsetti 1997, Italy Falsetti 1999, Italy^b^	Hirsute women with PCOS/Department of Gynaecological Endocrinology of the University of Brescia	1: 22 1: 32	**1:** FIN 5 mg once/day	2: 22 2: 32	**2:** FLU 250 mg b.i.d.	6 months 12 months	Hirsutism, SHBG, Testosterone, free testosterone, DHEAS, Androstenedione, fasting insulin, GI severe.	Barrier contraception	High^a^
Gambineri 2004, Italy Gambineri 2006, Italy	Overweight women with PCOS/Division of endocrinology S. Orsola-Malpighi Hospital, Italy	1: 10 2: 10 1: 17 2: 20	**1:** FLU 250 mg orally b.d. + hypocaloric diet **2:** MET 850 mg orally b.d. + 250 mg FLU 250 mg orally twice/day + hypocaloric diet	3: 10 4: 10 3:20 4: 19	**3:** MET 850 mg orally b.d. + hypocaloric diet **4:** PLAC + hypocaloric diet	6 months 12 months	Body weight, BMI, waist circumference, Hirsutism, frequency of menstruation, total testosterone, free-androgen index, androstenedione, DHEA-S, SHBG, fasting glucose, fasting insulin, QUICKI, ISI, LDL, HDL, Triglycerides Gambineri 2004 added: HOMA,	Non-hormonal contraception	Low^a^
Ganie 2004, India	Women with PCOS/Attending Endocrine and Metabolism Clinical of the All-India Institute of Medical Sciences between 2001 and 2002	1: 34	**1:** SPL 50 mg/day + lifestyle advice	2: 35	**2:** MET 1000 mg/day + lifestyle advice	6 months	BMI, WHR, Menstrual cyclicity, hirsutism, fasting blood glucose, HOMA, Testosterone	Barrier contraception	Moderate^a^
Ganie 2013, India	Women with PCOS/Tertiary care referral centre	1: 51 2: 62	**1:** SPL 50 mg/day + diet counselling **2:** SPL 50 mg/day + MET 1000 mg/day + diet counselling	3: 56	**3:** MET 1000 mg/day + diet counselling	6 months	Body weight, BMI, WHR, Menstrual cyclicity, Hirsutism, testosterone, fasting glucose, fasting insulin, HOMA-IR, QUICKI	Barrier contraception	Low^a^
Hagag 2014, Israel	Women with hirsutism due to PCOS/University affiliated endocrinology clinic, Israel	1: 72 2: 70	**1:** 250 μg NOR + 35 μg EE [250 μg NOR + 35 μg EE] + 100 mg SPL **2:** [2 mg CPA + 35 μg EE] + 10 mg CPA added	3: 25	**3:** 250 μg NOR + 35 μg EE	12 months	Weight change, acne, Adverse events (Nausea, Breast tenderness, Nipple discharge, menorrhagia, headache, etc)	COCP (as part of intervention)	Moderate^a^
Ibanez 2004, Spain	Nonobese adolescents and adults with PCOS/Endocrinology Unit, Hospital Saint Joan de Deu, University of Barcelona	1: 16 2: 11	**1:** MET 850 mg + FLU 62.5 mg (adults) **2:** OCP + MET 850 mg + FLU 62.5 mg	3: 16 4: 11	**3:** EE 30 μg + 0.3 mg DRSP (adolescents) **4:** EE 30 μg + 0.3 mg DRSP (adults)	9 months	BMI, Hirsutism, Fasting glucose/insulin ratio, SHBG, Testosterone, TG, HDL, LDL	Adolescents abstained from sex (no need for contraception); Adults were on COCP (as part of intervention)	Moderate^a^
Ibanez 2020, Spain de Zegher 2021, Spain Ibanez 2017, Spain Malpique 2019, Spain^b^ Diaz 2018, Spain^b^	Nonobese adolescents with PCOS/Endocrinology Unit, Hospital Saint Joan de Deu	1: 31 1: 29 1: 18	**1:** SPL 50 mg/d + pioglitazone 7.5 mg/d + metformin 850 mg/d (SPIOMET).	2: 31 2: 29 1: 18	**2:** EE 20 μg –levonorgestrel 100 mg	12 months (and 12 months post-treatment)	BMI, hirsutism score, SHBG, TT, Androstenedione, insulin, HOMA, OGTT, TG, LDL, HDL, CRP, de Zegher: FAI, TT Ibanez 2017: Acne scores	Advice on contraception–abstained from sex (no need for contraception)	Moderate
Mazza 2014, Italy	Overweight and obese women with PCOS/Endocrine Unit of University Magna Graecia of Catanzaro	1: 28	**1:** SPL 25 mg/day + MET 1700 mg/day + lifestyle modification (HCD: 1300 kcal/d)	2: 28	**2:** MET 1700 mg/day + lifestyle modification (HCD: 1300 kcal/d)	6 months	Weight, BMI, Hirsutism, cholesterol, HDL, LDL, triglycerides, fasting glucose, fasting insulin, HOMA, total testosterone, SHBG, FAI, DHEAS	Low-dose SPL	Low^a^
Mehrabian 2016, Iran	Women with PCOS/Midwifery clinic of Al-Zahra Hospital	1: 34	**1:** FLU 62.5 mg + OCP (0.03 mg EE + 0.15 mg LVG)	2: 34	**2:** MET 1000 mg/day	6 months	Waist circumference, triglycerides, fasting blood glucose, CRP, HDL, BMI,	COCP (as part of intervention)	Low^a^
Meyer 2007, Australia Burchall 2017, Australia	Overweight women with PCOS	1: 33 1: 16	**1:** 50 mg SPL + low-dose OCP b.d. (EEμg + LVG)	2: 31 3: 36 2: 21 3: 23	**2:** High-dose OCP (35 μg EE + 2 mg CPA) **3:** MET 2000 mg/day	6 months	Weight, BMI, OGTT, insulin, HOMA, testosterone, OGTT, Cholesterol, LDL, HDL, TG, CRP, TT, SHBG, FAI	COCP (as part of intervention)	Moderate^a^
Moretti 2018, Italy	Women with PCOS/Unit of endocrinology, section of reproductive endocrinology, University of Rome	1: 28	**1:** OCP (EE 0.030 mg + DRSP 2 mg or CPA 2 mg or dienogest 2 mg) + BC 50 mg o.d.	2: 24	**2:** OCP 0.030 mg + DRSP 2 mg or CPA 2 mg or dienogest 2 mg) + PLAC	12 months	Hirsutism, weight, BMI, total cholesterol, HDL, triglycerides, LDL, fasting glucose	COCP (as part of intervention)	Moderate^a^
Spritzer 2000, Brazil	Women with PCOS/Gynaecological Endocrinology Unit at Hospital	1: 10	**1:** 200 mg/d SPL, 20 d/month	2: 9	**2:** CPA 50 mg/day, 20 d/month + 35 mg/d EE over the last 10 days of CPA	12 months	Hirsutism	Barrier contraception	Moderate^a^
Tartagni 2000, Italy	Women with PCOS/Outpatients in an academic research environment	1: 9	**1:** FIN + Diane-35 (CPA 2 mg + EE 35 μg)	2: 9	**2:** Diane-35 (CPA 2 mg + EE 35 μg)	6 months	Hirsutism, free testosterone, DHEAS, SHBG, Androstenedione	COCP (as part of intervention)	High^a^
Tartagni 2004, Italy	Women with hirsutism due to PCOS/Obstetrics and gynaecology outpatient clinic	1: 8	**1:** FIN 2.5 mg/day	2: 8	**2:** FIN 2.5 mg every 3 days	10 months	Total testosterone, DHEAS, SHBG, androstenedione, BMI, Hirsutism	Non hormonal contraception	High^a^
Tartagni 2014, Italy	Women with hirsutism due to PCOS/Obstetrics and gynaecology outpatient clinic	1: 7	**1:** FIN 2.5 mg every 3 days	2: 7	**2:** Placebo	6 months	BMI, Hirsutism, SHBG, Testosterone, DHEAS, Androstenedione, GI-related adverse effects	Non-hormonal contraception	High^a^
Vieira 2012, Brazil	Women with PCOS/University Hospital of Ribeirao Preto School of Medicine between 2007 and 2009	1: 20	**1:** OCP (2 mg CPA + 30 mcg EE) + SPL 100 mg/day	2: 21	**2:** OCP (2 mg CPA + EE 30 mcg)	12 months	Weight, BMI, SHBG, FAI, Testosterone, free testosterone, fasting insulin, fasting glucose, total cholesterol, HDL, LDL, HOMA, CRP	COCP (as part of intervention)	Moderate^a^

**Abbreviations**: **b.i.d,** two times daily; **BC**, bicalutamide; **BMI**, body mass index; **CPA**, cyproterone acetate; **CRP**, c-reactive protein; **DHEAS**, dehydroepiandrosterone sulphate; **EE**, ethinylestradiol; **FAI**, free androgen index; **FLU**, flutamide; **FIN**, finasteride; **GI**, glycaemic index; **HCL**, hypocaloric diet; **HDL**, high density lipoprotein; **HOMA-IR**, homeostasis model assessment-estimated insulin resistance; **IR**, insulin resistance; **LDL**; low density lipoprotein; **LVG**, levonorgestrel; **MET**, metformin; **MA**, meta-analysis; **OCP/COCP**, oral contraceptive pill/combined oral contraceptive pill; **OGTT**, oral glucose tolerance test; **PLAC**, placebo; **PCOS**, polycystic ovary syndrome; **QUICKI,** the quantitative insulin-sensitivity check index; **SHBG**, sex hormone binding globulin; **SPL**, spironolactone; **TT**, total testosterone.

a Risk of bias derived from 2018 PCOS guideline.

b No additional outcomes from Diaz 2018 and Malpique 2019–no data were extracted in these circumstances.

**Table 3:** Summary of meta-analysis results examining the use of anti-androgens in polycystic ovary syndrome.

Comparison^a^	Studies (participants), duration	BMI/Weight	Hirsutism/Acne	Hormonal measures	Glycaemic/IR measures	Lipids	Menstruation
**AA + LS** vs Placebo + LS (adults)	2 (89) 6–12 months	NS = BMI	NS = hirsutism	NS= SHBG	–	–	–
**AA daily** vs every 3 days (adults)	2 (80) 10–12 months	–	↓ hirsutism	↓ Androstenedione NS = DHEAS, SHBG, T	–	–	–
**AA + LS** vs Metformin + LS (adults)	2–4 (89–265) 6–12 months	NS = Weight, BMI, WHR	↓ hirsutism	↑ SHBG NS = T, DHEAS	↓ fasting insulin; ↓ fasting insulin: glucose ratio NS= FBG, QUICKI	–	↑ frequency
**AA + Metformin + LS** vs Metformin + LS (adults)	2–4 (92–262) 6–12 months	NS = Weight, BMI, WHR	NS = hirsutism	↓ T NS= SHBG, DHEAS	↓ FBG NS = fasting insulin, HOMA-IR	NS= HDL, LDL, TG	–
**AA + LS** vs AA + Metformin + LS (adults)	2–3 (91–204), 6–12 months	NS = BMI	NS = hirsutism	NS= SHBG, T	↑ FBG NS = QUICKI, fasting insulin	–	–
**AA + COCP** vs COCP ± Placebo (adults)	2–3 (59–130), 6–12 months	NS = Weight, BMI	–	NS = T, SHBG	–	↑ TG, ↑ LDL, ↑ TC NS= HDL	–
**AA + COCP** vs Metformin (adults)	2 (107) 6 months	NS = BMI	–	–	–	–	–

AA, anti-androgens; BMI, body mass index; DHEAS, dehydroepiandrosterone sulphate; FAI, free androgen index; FBG, fasting blood glucose; HDL/LDL, high-density/low-density lipoprotein; HOMA-IR, homeostatic model assessment of insulin resistance; IR, insulin resistance; ISI, insulin sensitivity index; QUICKI, Quantitative insulin-sensitivity check index; LS, lifestyle; NS, not significant; OGTT, oral glucose tolerance test; SHBG, sex-hormone binding globulin; T, testosterone; TC, total cholesterol; TG, triglycerides; WHR, waist hip ratio.

a Effect reported in the table relates to the intervention listed in bold (e.g. for the first row, the up or down arrows indicate higher or lower effect for each outcome, respectively, with the AA + LS intervention).

Overall, sample sizes varied from 11 to 167 participants, with treatment durations ranging from six to 12 months. Studies were conducted in Italy (n = 7), Spain (n = 3), Brazil (n = 2), Iran (n = 2), India (n = 2), Australia (n = 1), Israel (n = 1), Turkey (n = 1), and the United States of America (n = 1). Anti-androgen medications included spironolactone, finasteride, flutamide, and bicalutamide, with comparators including placebo, metformin, COCP, lifestyle, or a combination of these. Most of the included studies were in adults, with two in adolescents. The mean BMI of participants ranged from healthy (18.5–24.9 kg/m^2^) to overweight (25.0–29.9 kg/m^2^) and obese (≥30.0 kg/m^2^), but most studies were in women with a BMI ≥25.0 kg/m^2^. Methods of contraception included abstinence, barrier, or hormonal contraceptives as part of the intervention or comparison, and were reported in all studies (as this was an eligibility criterion).

In quality appraisal, four studies were classified as being of low RoB, nine were moderate, and seven were high risk. The most common reasons for high RoB ratings were the lack of concealed or centralised allocation, absence of participant and/or investigator blinding, high drop-out rate, and lack of pre-registered protocols (Table S1). In the GRADE assessments, the certainty of evidence in the reported outcomes mostly ranged from very low to low, due to concerns regarding RoB, small sample sizes (imprecision), and statistical heterogeneity (inconsistency), with very few outcomes ranging from moderate to high (detailed in the Supplementary Material).

### Placebo and lifestyle comparisons

#### Anti-androgen vs placebo—adolescents and adults

One high RoB trial compared low-dose finasteride (2.5 mg daily) vs placebo for six months in 14 adolescents with PCOS (age 15–19 years).^26^ Hirsutism assessed by Ferriman-Gallwey (FG) scores was lower with anti-androgens compared with placebo (WMD [95% CI]: −4.00 [−6.81, −1.19], p = 0.005), but dehydroepiandrosterone sulphate (DHEAS) was higher (0.60 μmol/l [0.07, 1.13], p = 0.03). There were no differences in BMI, testosterone, sex-hormone binding globulin (SHBG), or androstenedione (Supplementary Material). One moderate RoB trial compared low-dose flutamide (125 mg daily) vs placebo for six months in 11 normal-weight adults with PCOS.^27^ In single study analysis, there were no differences in any outcomes assessed, including weight, BMI, lipids, DHT, DHEAS, androstenedione, total and free testosterone, or glycaemic measures (Supplementary Material).

#### Anti-androgen + lifestyle vs placebo + lifestyle—adults

Two RCTs compared anti-androgens (flutamide 250 mg twice daily) + lifestyle intervention with placebo + lifestyle in adult women with PCOS, one with 6-month follow-up with high RoB,^28^ and the other 12 months with low RoB.^29^ In meta-analysis of these two RCTs (n = 89; Table S2), there were no differences in BMI (WMD [95% CI]: −3.08 kg/m^2^ [−8.67, 2.50], hirsutism (−0.93, [−3.37, 1.51], p = 0.45; *I*^*2*^ = 76%) or SHBG (9.72 nmol/l [−0.71, 20.14], p = 0.07; *I*^*2*^ = 31%), while other outcomes could not be assessed due to discrepancies in units in the high RoB study by Amiri et al.^28^ (Supplementary Material).

In examining the single low RoB study by Gambineri et al.,^29^ anti-androgens (flutamide 250 mg twice daily) + lifestyle resulted in lower body weight (−17.00 kg, [−25.37, −8.63], p < 0.0001), DHEAS (−2.34 μg/ml [−4.06, −0.62], p = 0.008), frequency of menstruation (−0.80 [−1.54, −0.06], p = 0.03), fasting insulin (−4.00 μU/ml [−6.98, −1.02], p = 0.009), low-density lipoprotein (LDL) cholesterol (−21.00 mg/dl [−40.93, −1.07], p = 0.04) and triglycerides (−50.00 mg/dl [−77.60, −22.40], p = 0.0004), and higher quantitative insulin-sensitivity check index (QUICKI) (0.04 [0.02, 0.06], p < 0.0001) and insulin sensitivity index (5.10 [2.32, 7.88], p = 0.0003), compared with placebo + lifestyle. There were no differences in other outcomes assessed (Table 3 and Supplementary Material).

### Anti-androgen comparisons

#### Anti-androgen (daily) vs anti-androgen (every 3 days)—adults

Two high RoB studies were pooled in meta-analysis, comparing finasteride regimens (2.5 mg once daily vs every 3 days) or flutamide regimens (250 mg twice daily vs every three days) for 10–12 months.^30^^,^^31^ In meta-analysis, daily anti-androgen use was superior for reducing hirsutism (WMD [95% CI]: −3.48 FG score [−4.58, −2.39], p < 0.0001) and androstenedione (−0.30 ng/ml [−0.50, −0.10], p = 0.004), with no differences in SHBG, testosterone, or DHEAS (n = 80; Table S3). For outcomes reported in a single study (n = 64),^30^ there were no differences in BMI or adverse effects such as decreased libido or headache; however, dry skin was higher in the daily anti-androgen group (OR [95% CI]: 6.60 [2.21, 19.73], p = 0.0007) and three women dropped out with treatment of flutamide due to liver toxicity (high transaminase levels).

### Metformin comparisons

#### Anti-androgens vs metformin with or without anti-androgens—adults

A single high RoB trial compared anti-androgens (finasteride 5 mg once daily) alone or with metformin vs metformin alone for 12 months in adult women with PCOS.^32^ There were no differences in BMI, hirsutism, free testosterone, SHBG, DHEAS, androstenedione, or HOMA-IR in either comparison (Supplementary Material).^32^

#### Anti-androgens + lifestyle vs metformin + lifestyle—adults

Four RCTs^28^^,^^29^^,^^33^^,^^34^ reported the use of anti-androgens (flutamide 250 mg twice daily or spironolactone 50 mg once or twice daily) + lifestyle compared with metformin + lifestyle for 6–12 months in adult women with PCOS. Two had a low RoB,^29^^,^^33^ one moderate^34^ and one high.^28^ The study by Amiri et al.^28^ was excluded from meta-analysis in circumstances where the accuracy of units was unclear. In meta-analysis, anti-androgens + lifestyle resulted in lower hirsutism (n = 265, WMD [95% CI]: −1.59 [−3.06, −0.12], p = 0.03), fasting insulin (n = 213; −2.11 μU/ml [−3.97, −0.26], p = 0.03) and fasting glucose-insulin ratio (n = 176; −1.12 [−1.44, −0.79], p < 0.0001), and higher HDL (n = 37; 0.21 mmol/l [0.05, 0.37], p = 0.01), frequency of menstruation (n = 176, 0.79 cycles/year, [0.05, 1.53], p = 0.04) and SHBG (n = 89; 7.70 nmol/l [0.75, 14.66], p = 0.03), compared with metformin + lifestyle (Table S4). No differences in other outcomes were found (Table 3 and Supplementary Material). Of note, when removing Amiri et al.^28^ in a sensitivity analysis, hirsutism and SHBG were no longer significant (p = 0.11 for both; not shown).

#### Anti-androgens + metformin + lifestyle vs metformin + lifestyle—adults

Four RCTs compared anti-androgens (flutamide 250 mg twice daily or spironolactone 25–50 mg daily) + metformin + lifestyle vs metformin + lifestyle alone, with 6–12 months follow-up in adult women with PCOS. Three had low RoB^29^^,^^33^^,^^35^ and one had high RoB.^28^ In meta-analysis (n = 262; Table S5), the anti-androgen + metformin + lifestyle group had lower testosterone (WMD [95% CI]: −0.29 nmol/l [−0.52, −0.06], p = 0.01) and fasting glucose (−2.93 mg/dl [−5.78, −0.09], p = 0.04). In sensitivity analysis excluding the high RoB study by Amiri et al.,^28^ WHR favoured the anti-androgen + metformin + lifestyle intervention (n = 118; −0.03 [−0.06, −0.00], p = 0.02; not shown), but the difference in fasting glucose was no longer significant (p = 0.12; not shown).

For outcomes analysed in a single study,^29^ the anti-androgens + metformin + lifestyle intervention resulted in a higher insulin sensitivity index, with no differences in other outcomes (Table 3; Supplementary Material).

#### Anti-androgens + metformin + lifestyle vs. anti-androgens + lifestyle—adults

Three RCTs of 6–12 month duration in adult women with PCOS compared anti-androgens (flutamide 250 mg once or twice daily, or spironolactone 50 mg daily) + lifestyle + metformin vs anti-androgens + lifestyle only, of which two had low RoB^29^^,^^33^ and one high.^28^ In meta-analysis, anti-androgen + lifestyle only (without metformin) resulted in higher fasting glucose (n = 204, WMD: 3.81 mg/dl, 95% CI: 1.35, 6.28, p = 0.002; Table S6). There were no differences in other outcomes (Table 3; Supplementary Material). Results were unchanged in sensitivity analysis after removing the high RoB study by Amiri et al.^28^ (not shown).

For outcomes assessed in single studies by Gambineri et al.^29^ or Ganie et al.,^33^ anti-androgen + lifestyle resulted in higher WHR (n = 113; 0.05 [0.03, 0.07], p < 0.0001) than the metformin combination, with no differences in other outcomes (Table 3; Supplementary Material).

### COCP comparisons

#### Anti-androgens vs anti-androgens + COCP—adults

One moderate RoB trial compared 12-month treatment with spironolactone (200 mg daily) vs CPA (50 mg daily) + ethinyl estradiol (35 μg daily) for hirsutism in adult women with idiopathic hirsutism and PCOS.^36^ In the PCOS subgroup (n = 19), hirsutism was lower with the combination treatment (WMD: −4.00 [−4.90, −3.10], p < 0.0001).

#### Anti-androgens + COCP vs COCP with or without placebo—adults

A total of six RCTs compared anti-androgens (spironolactone 50–100 mg daily, finasteride 5 mg daily, or bicalutamide 50 mg daily) + COCP with COCP ***±*** placebo, with 6–12 months follow-up in adult women with PCOS. Five had moderate RoB^37^, ^38^, ^39^, ^40^, ^41^ and one had a high RoB.^42^ In meta-analyses of two studies (n = 93), total cholesterol (WMD [95% CI]: 20.81 mg/dl [7.81, 33.82], p = 0.002), LDL (15.12 mg/dl [3.20, 27.04], p = 0.01), and triglycerides (41.34 mg/dl [20.26, 62.42], p = 0.0001) were higher with anti-androgens + COCP compared with COCP alone, with no difference in other outcomes (Table S7, Supplementary Material). In sensitivity analysis excluding the high RoB study^42^ from the assessment of SHBG, SHBG was lower in the anti-androgen + COCP group compared to COCP alone (WMD: −84.40 nmol/l [−142.23, −26.57], p = 0.004; not shown). An additional sensitivity analysis was conducted to exclude Moretti et al.^40^ since this was the only placebo-controlled study that used bicalutamide while the others used either spironolactone or finasteride. Most results remained unchanged, except for LDL and triglycerides which were no longer significant (p = 0.06 and p = 0.22; not shown).

In single study analyses (n = 41), fasting insulin (3.50 μIU/ml [0.20, 6.80], p = 0.04), HOMA-IR (0.70 [0.02, 1.38], p = 0.04), and FAI (0.50 [0.01, 0.99], p = 0.04), were higher in the anti-androgen + COCP compared to the COCP alone or with placebo,^41^ with no differences in fasting glucose (p = 0.24; Supplementary Material).

Other outcomes or studies were assessed using descriptive analyses as data were not amenable to single-study or pooled meta-analyses. In the study by Moretti et al. (n = 52),^40^ there were no differences in fasting glucose, or in side effects including alanine transaminase (ALT), aspartate aminotransferase (AST), menorrhagia, hypercholesterolaemia, hypertriglyceridaemia, dysmenorrhoea, menstrual spotting, and minor depressive state/mood reduction between the groups. Meyer et al.^39^ (n = 64) reported that both high-dose COCP (35 μg ethinyl estradiol + 2 mg CPA) and low-dose COCP (20 μg ethinyl estradiol + 100 μg levonorgestrel) + anti-androgens (spironolactone 50 mg twice daily) reduced testosterone, DHEAS, and FAI, while increasing SHBG. There were small but significant changes in lipids, mostly in the higher-dose COCP group.^39^ The follow-up sub-study that assessed additional outcomes by Burchall et al.^37^ (n = 38) also reported similar reductions in hormonal parameters as those in the main study by Meyer et al.^39^ In Hagag et al. (n = 97),^38^ treatment with COCP + anti-androgens (spironolactone 100 mg daily) was more effective than COCP monotherapy for reducing hirsutism. Tartagni et al.^42^ (n = 18) reported improvements in DHT in the anti-androgens + COCP compared with the COCP group at 6 months, with within-group improvements in free testosterone, DHT, DHEAS, and SHBG from baseline values.

#### Anti-androgens + COCP vs metformin—adults

Four RCTs compared anti-androgens (flutamide 62.5 mg daily or spironolactone 50–100 mg daily) + COCP vs metformin for 6–12 months in adult women with PCOS (n = 207), one of which had low RoB,^43^ two moderate,^37^^,^^39^ and one high.^44^ Two studies could be combined in meta-analysis for one outcome, BMI, showing no difference (p = 0.93, Table S8). In descriptive analysis, the study by Alpañés et al.^44^ reported that hirsutism, total and free testosterone, androstenedione, DHEAS and menstrual dysfunction were lower with anti-androgens + COCP compared with metformin. Meyer et al.^39^ also reported that testosterone and DHEAS reduced with anti-androgens (spironolactone 50 mg daily) + COCP, but did not change with metformin; whereas, insulin resistance improved with metformin, but did not change in the anti-androgen + COCP group. In the study by Burchall et al.^37^ (n = 68), the anti-androgen + COCP group had lower C-reactive protein (CRP) and rates of menstrual dysfunction, while fasting blood glucose was higher, compared with metformin.

#### Anti-androgens + metformin (with or without COCP) vs COCP—adults and adolescents

A single moderate RoB trial investigated the combination therapy of anti-androgens (flutamide 62.5 mg daily) + metformin with or without COCP vs COCP alone for nine months in adults and adolescents with PCOS.^45^ In adults (n = 32), the anti-androgen + metformin group (without COCP) had reduced SHBG, triglycerides, LDL and HDL compared with COCP alone (Supplementary Material). In adolescents (n = 22), SHBG was lower with anti-androgens + metformin + COCP, compared to COCP alone (Supplementary Material).

#### Anti-androgens + metformin + pioglitazone (SPIOMET) vs COCP—adolescents

One study (reported in five articles/sub-studies)^46^, ^47^, ^48^, ^49^, ^50^ with moderate RoB compared low-dose anti-androgens (spironolactone 50 mg daily) + metformin + pioglitazone (SPIOMET) against COCP for 12 months in adolescent girls with PCOS (n = 62 in the main study). The SPIOMET group had reduced hirsutism (WMD [95% CI]: −3.00 [−5.77, −0.23], p = 0.03), SHBG (−29.00 nmol/l [−39.56, −18.44], p < 0.0001), fasting insulin (−62.00 pmol/l [−81.40, −42.60], p < 0.0001), ALT (−0.09 μkat/l [−0.16, −0.02], HOMA-IR (−1.80 [−2.42, −1.18], p < 0.0001), LDL (−0.05 mmol/l [−0.78, −0.22] p = 0.0004), CRP (−18.10 mmol/l [−25.75, −10.45], p < 0.0001), and FAI (−2.20 [−4.38, −0.02], p = 0.05), but higher androstenedione (1.00 nmol/l [0.29, 1.71], p = 0.006) compared with the COCP group.

## Discussion

To our knowledge, this systematic review and meta-analysis is the first evidence synthesis of RCTs investigating the efficacy and safety of anti-androgen pharmacological agents, alone or in combination, on endocrine and metabolic features in adolescents and adults with PCOS. Our findings address key gaps in the literature and directly inform the current 2023 update of the International Evidence-based Guideline for the Assessment and Management of PCOS. Based on findings from this review, the guideline recommends that anti-androgen pharmacological agents could be considered to treat clinical hyperandrogenism (hirsutism), in situations where the COCP and/or cosmetic therapies (including mechanical laser and light therapies for hair reduction) are contraindicated, poorly tolerated, or present a sub-optimal response after a minimum period of six months. Where appropriate, the use of effective contraception is strongly recommended, and women should be advised that anti-androgens may cause under-virilisation of a male fetus. It should be noted that this recommendation is based on the best available evidence, which remains largely limited as noted by the high heterogeneity (12 comparisons across 20 studies) and the low to very low GRADE certainty across most outcomes. Therefore, the recommendation remains general, and should not override clinical judgment with consideration of individual circumstances and perspectives.

In relation to safety, specific recommendations on optimal doses or formulations cannot be made based on the available evidence, due to high levels of heterogeneity across the included studies, with a considerable number of comparisons. In view of the limited data in PCOS, ascertaining the required information from general population literature may be appropriate. Based on general population recommendations, spironolactone doses of 25–100 mg daily appear to have low risks of adverse side effects, while high doses of CPA (≥10 mg) may lead to meningioma or venous thromboembolism, and flutamide and bicalutamide are associated with increased risks of liver toxicity.^51^ Most importantly, anti-androgens should not be used in the absence of effective contraception in sexually active individuals, due to the risks of under-virilisation of a male fetus if an unplanned pregnancy occurs.^52^

Moreover, combination therapy with anti-androgens + COCP compared to COCP (±placebo) resulted in poorer lipid profiles in adult women with PCOS. This is consistent with other studies reporting that hormonal contraception may alter lipid profiles,^53^ potentially via alterations in the estrogen receptor that affects hepatic apolipoprotein upregulation.^54^ However, when removing the study by Moretti et al.^40^ from the meta-analysis, the only study to use bicalutamide, there was no longer a significant difference in lipids. This indicates that bicalutamide, while effective for some PCOS outcomes such as hirsutism, worsens lipid metabolism. The risk of liver toxicity with bicalutamide has been reported in the general population, although risks of serious liver toxicity and lipid-related lung diseases are rare.^55^ This is concerning as PCOS is already a condition with intrinsic metabolic and lipid abnormalities, which could be further exacerbated by bicalutamide therapy. Flutamide has also been associated with hepatotoxicity,^56^ which was reflected in this systematic review where three women dropped out from the study by Falsetti et al.^30^ due to flutamide-induced hepatotoxicity. Therefore, due to the risks of liver toxicity and dyslipidaemia, the use of bicalutamide or flutamide is not recommended for treating clinical hyperandrogenism in women with PCOS.

While other anti-androgen pharmacological agents, such as spironolactone, may be considered for treating clinical hirsutism, first-line therapy remains the COCP.^57^ This is because the COCP can improve menstrual cyclicity while providing effective contraception, whereas anti-androgens are teratogenic since they can interfere with external genital development of male fetuses.^52^ Consistent with our findings, a systematic review and network meta-analysis by Barrionuevo et al.^17^ of 43 studies reported that anti-androgens in combination with the COCP may have additive benefits for hirsutism, noting important methodological limitations in the available evidence. However, Barrionuevo et al.^17^ investigated women with idiopathic hirsutism and specifically excluded women with PCOS. An earlier systematic review of 28 studies by Koulouri et al.^18^ identified improvements in hirsutism following anti-androgen treatment, which were associated with BMI; however, this review included broader hirsute populations, rather than PCOS specifically, and is likely outdated given that the search was conducted 17 years ago (in 2006). Although PCOS constitutes 65–75% of hirsutism cases,^5^ other causes of hirsutism such as Cushing's syndrome, androgen-producing tumours, hyperprolactinaemia, and peripheral androgen activity, may necessitate different and more nuanced treatment approaches than those needed in PCOS,^58^ particularly due to the cardiometabolic implications of the latter. Here, we extend the potential benefits of anti-androgens on hirsutism to women with PCOS, while maintaining that COCP remains first-line therapy pending further study.

Anti-androgens and COCPs can reduce hirsutism via different mechanisms. The COCP increases SHBG production and decreases luteinising hormone (LH)-induced stimulation of theca cells, leading to reduced circulating free testosterone concentrations.^59^ Conversely, anti-androgens promote reductions in clinical hyperandrogenism by competitively inhibiting androgen-binding receptors, likely via inhibiting 5-α-reductase, as well as by other mechanisms which are hitherto not well-understood. Although early research indicated that spironolactone may interfere with androgen synthesis,^60^ these data have not been replicated or validated using modern techniques. Therefore, further studies elucidating the mechanisms of action of anti-androgens are warranted.

This is, to our knowledge, the first systematic review and meta-analysis examining the use of anti-androgens specifically in women with PCOS. We conducted a comprehensive search, using international gold-standard methodology for identifying, evaluating, and appraising the literature (including the GRADE tool), in line with the internationally endorsed PCOS guideline methodology. All data extraction and quality assessments were conducted in duplicate and/or cross-checked to limit subjectivity bias and maximise accuracy. We included both adult and adolescent populations, with a wide range of outcomes relevant to PCOS as determined by experts and consumer groups in the guideline development process, thereby providing broad insights into the efficacy of anti-androgens, with or without other interventions, in managing key features of PCOS.

Several limitations in the literature were apparent. Most notably, significant heterogeneity in the included studies and variations in methodological quality were evident, with many studies having small sample sizes and varied interventions, comparators, doses, frequencies, and population characteristics. This was reflected in the GRADE certainty of evidence, which was classified as very low or low for the majority of outcomes. Given that the 20 included studies represented 12 different comparisons, this heterogeneity precluded meta-analysis for many comparisons, with limited sensitivity analyses, and we were unable to perform sub-group stratification by important variables including age, weight/BMI, insulin resistance, lifestyle variables or severity of symptoms, given the small sample sizes across most comparisons. In addition, separate assessments of specific anti-androgens were not possible and there were insufficient studies in adolescents (n = 2 trials) to allow for meaningful comparisons. For these reasons, the results presented herein may not reflect the real-life experiences of all patients with PCOS or their treating clinicians, and treatments should be tailored to individual needs and preferences. In the meantime, further high-quality, well-powered RCTs are needed to clarify the effects of anti-androgens in women with PCOS with greater certainty. In particular, given that only two studies were identified in adolescents in this review, studies of adolescents as well as ‘at risk’ populations (adolescents with PCOS features, but not meeting formal diagnostic criteria) are lacking and this remains a key gap in the evidence. Other research priorities include large-scale studies comparing efficacy of different anti-androgen types, doses, combinations and treatment schedules, as well as optimal monitoring methods for adverse events. Studies should be sufficiently sized to allow for exploration of efficacy among specific phenotypes and by important variables including weight, age and metabolic parameters. For comparisons that show differences, validation in large scale population-based studies would be of value.

Limitations in the review process should also be noted. Due to being poorly reported or presented in a format which was not amenable to meta-analysis, some data were excluded, leading to missing or incomplete information for some comparisons. Although exclusion of these data was necessary to ensure consistency and rigor in the review process, this meant that sample sizes were small for many comparisons and it is possible that their inclusion may have impacted on our results, urging caution in the interpretation of these findings. We addressed this limitation to some extent via sensitivity analysis by RoB; however, this was not possible for all comparisons or outcomes due to the small number of included studies. Moreover, studies in languages other than English were excluded due to resource and time constraints, and some full texts were not found; hence, potentially relevant results may have been excluded from the review. Lastly, publication bias cannot be ruled out given that this could not be adequately judged by inspection of funnel plots (since less than 10 studies were assessed in each analysis) and grey literature (unpublished work) was not sourced or included in the review.

In summary, anti-androgens may have possible benefits on clinical hyperandrogenism (i.e., hirsutism) in combination with effective contraception but, per guideline recommendations, should be used in circumstances where COCP (and/or cosmetic options including mechanical laser and light therapies for hair reduction) are contraindicated, poorly tolerated, or have been ineffective for a minimum period of 6 months. This general recommendation is based on the best available evidence, which remains limited due to high heterogeneity; hence, application should incorporate clinical judgement, contextual factors and individual characteristics and preferences. While the optimal types and doses of anti-androgens in PCOS cannot be determined from the available evidence, general population data suggests that 25–100 mg daily of spironolactone appears to be safe. Based on the available evidence, COCPs remain the recommended first-line therapy for clinical hyperandrogenism in PCOS, until further high-quality, adequately powered studies can demonstrate further benefits for anti-androgens in the context of PCOS.

## Contributors

SA, MF, and JM conducted the screening process, risk of bias, and GRADE assessments. SA conducted the meta-analysis, evidence synthesis, and wrote the first draft of the manuscript, supervised by AM. SA, MF, JM and AM accessed and verified the underlying data. SW, DR, and AV were the clinical leads that provided content expertise and scoped the review. CTT, HT, and AM supervised the review process and provided input on meta-analysis and systematic review methodologies. All authors reviewed and edited the manuscript contributing substantial intellectual input in line with ICMJE criteria for authorship and approved the final version for publication.

## Data sharing statement

All data are secondary and are available in the original published studies. Data produced through meta-analysis will be available with publication (in the manuscript and/or Supplementary Material). Additional information can be supplied by the corresponding author upon reasonable request.

## Declaration of interests

All authors declare no competing interests. JM received funding from the Orion Research Foundation and the Medical Society of Finland. MF received funding from the Gothenburg Medical Association, Sahlgrenska University Hospital, the Iris Foundation and the Hjalmar Svensson Foundation, as well as honoraria from Gedeon Richter. HT received funding from the National Health and Medical Research Council (NHMRC) through the Centre for Research Excellence for Women's Health in Reproductive Life (CRE-WHiRL). SFW received support from the NHMRC-funded guideline to attend the guideline meeting and is a member of the board of directors for the Paediatric Endocrine Society. CTT is supported by CRE- WHiRL and chairs the Androgen Excess and Polycystic Ovary Syndrome Society Early Career Special Interest Group. None of these funding organisations had any role in the study design, data collection and analysis, decision to publish, or preparation of the manuscript. All other authors declare no competing interests.
