# Supplementary material for: Efficacy and safety of anti-androgens in the management of polycystic ovary syndrome: a systematic review and meta-analysis of randomised controlled trials
Source: eClinicalMedicine. 2023 Aug 9;63:102162. doi: 10.1016/j.eclinm.2023.102162 (PMC10424142; doi:10.1016/j.eclinm.2023.102162)
Supplement: Supplementary Material [file mmc1.pdf]

Supplementary File 1

Search Strategies for Electronic Database Searches

OVID Medline, All EBM, PsycInfo, EMBASE (n = 1,246)	CINAHL (n = 414)
1 exp polycystic ovary syndrome/<br>2 polycystic ovar*.mp.<br>3 poly-cystic ovar*.mp.<br>4 PCO*.mp.<br>5 (stein-leventhal or leventhal).mp.<br>6 anovulation/<br>7 anovulat*.mp.<br>8 oligo-ovulat*.mp.<br>9 oligoovulat*.mp.<br>10 (ovar* adj5 (sclerocystic or polycystic or poly-cystic or degenerat* or hyperandrogen* or hyper-androgen*)).mp.<br>11 or/1-10<br>12 exp Contraceptives, Oral/<br>13 ((Oral and contracept*) or OCP or COCP).tw.<br>14 exp Metformin/<br>15 Metformin*.tw.<br>16 exp Androgen Antagonists/<br>17 *Spironolactone/<br>18 *Finasteride/<br>19 (anti?androgen* or anti androgen or androgen antagonist* or spironolactone or cyproterone acetate or finasteride or flutamide).mp.<br>20 exp Anti-Obesity Agents/<br>21 *Obesity/th [Therapy]<br>22 ((anti?obesity or anti obesity or weight loss) and (agent* or drug*)).mp.<br>23 (orlistat or sibutramine).mp.<br>24 *Inositol/<br>25 (inositol* or myo?inositol* or myo inositol* or meso?inositol* or meso inositol* or i-inositol* or epi?inositol* or epi inositol* or chiro?inositol* or chiro inositol* or l-chiro?inositol* or l-chiro inositol*).mp.<br>26 or/12-25<br>27 search$.tw. or meta-analysis.mp. or meta-analysis.pt. or review.pt. or di.xs. or associated.tw.<br>28 clinical trial.mp. or clinical trial.pt. or random.mp. or tu.xs.<br>29 27 or 28<br>30 11 and 26 and 29	**S26** S11 AND S25<br>**S25** S12 OR S13 OR S14 OR S15 OR S16 OR S17 OR S18 OR S19 OR S20 OR S21 OR S22 OR S23 OR S24<br>**S24** (inositol* or myo?inositol* or meso?inositol* or i-inositol* or epi? inositol* or chiro? inositol or l-chiro? inositol*)<br>S23 (MH "Inositol+")<br>**S22** (orlistat or sibutramine)<br>**S21** ((anti?obesity or weight loss or weight-loss) and (agent* or drug*))<br>**S20** (MH "Antiobesity Agents+")<br>**S19** (anti? androgen* or anti androgen or androgen antagonist* or spironolactone or cyproterone acetate or finasteride or flutamide)<br>**S18** (MH

Supplementary File 1

	"Finasteride")<br>**S17** (MH "Spironolactone+")<br>**S16** (MH "Androgen Antagonists+")<br>S15 metformin*<br>**S14** (MH "Metformin")<br>**S13** ((Oral and contracept*) or OCP or COCP)<br>**S12** (MH "Contraceptives, Oral+")<br>**S11** S1 OR S2 OR S3 OR S4 OR S5 OR S6 OR S7 OR S8 OR S9 OR S10<br>**S10** ovar* N5 sclerocystic or ovar* N5 polycystic or ovar* N5 polycystic or ovar* N5 degenerat* or ovar* N5 hyperandrogen* or ovar* N5 hyperandrogen*<br>**S9** oligoovulat*<br>**S8** oligo-ovulat*<br>**S7** SU anovulation<br>**S6** SU ovarian Cysts<br>**S5** stein-leventhal or Leventhal<br>**S4** PCO*<br>**S3** poly-cystic ovar*

Supplementary File 1

	**S2** polycystic ovar*<br>**S1** SU polycystic ovary syndrome
**Search string: (**exp polycystic ovary syndrome/ OR polycystic ovar*.mp. OR poly-cystic ovar*.mp. OR PCO*.mp. OR (stein-leventhal or leventhal).mp. OR anovulation/ OR anovulat*.mp. OR oligo-ovulat*.mp. OR oligoovulat*.mp. OR (ovar* adj5 (sclerocystic or polycystic or poly-cystic or degenerat* or hyperandrogen* or hyper-androgen*)).mp. AND (exp Contraceptives, Oral/ OR ((Oral and contracept*) or OCP or COCP).tw. OR exp Metformin/ OR Metformin*.tw. OR exp Androgen Antagonists/ OR *Spironolactone/ OR *Finasteride/ OR (anti?androgen* or anti androgen or androgen antagonist* or spironolactone or cyproterone acetate or finasteride or flutamide).mp. OR exp Anti-Obesity Agents/ OR *Obesity/th [Therapy] OR ((anti?obesity or anti obesity or weight loss) and (agent* or drug*)).mp. OR (orlistat or sibutramine).mp. OR *Inositol/ OR (inositol* or myo?inositol* or myo inositol* or meso?inositol* or meso inositol* or i-inositol* or epi?inositol* or epi inositol* or chiro?inositol* or chiro inositol* or l-chiro?inositol* or l-chiro inositol*).mp.) AND (search$.tw. or meta-analysis.mp. or meta-analysis.pt. or review.pt. or di.xs. or associated.tw. OR clinical trial.mp. or clinical trial.pt. or random.mp. or tu.xs.).	

List of Excluded Studies with Reasons

	Citation	Reason
1.	Unknown. Effect of green tea pills and metformin versus placebo on the Nrf2-antioxidant system and proinflammatory cytokines, including IL-6 and TNF-a, in peripheral blood mononuclear cells of women with polycystic ovary syndrome: a single blind randomized clinica 2017.	Wrong publication type.
2.	Effect of supplementation in treatment of women with polycystic ovary syndrome. Clinical trial of the effect of inofolic supplementation compared with metformin on parameters of mental health and oxidative stress in women with polycystic ovary syndrome 2017.	Wrong publication type.
3.	Effect of inofolic supplementation in treatment of women with polycystic ovary syndrome. Clinical trial of the effect of inofolic supplementation compared with metformin on metabolic profiles and gene expression related to insulin and lipid in women with polycystic ovary syndrome 2017.	Full text not obtainable.
4.	Comparison of oral contraceptives including Contrasmine, Etisterone and Desoceptive with Ovustop-L (LD) on clinical, biochemical and metabolic findings, and quality of life in women with polycystic ovary syndrome. A Randomized cross-over clinical trial to assess the effectiveness of oral contraceptives including Contrasmine, Etisterone and Desoceptive with Ovustop-L (LD) on clinical, biochemical and metabolic findings, and quality of life in women with polycystic o 2017.	Full text not obtainable.
5.	The efficacy of Fennel infusion and cupping on ovarian failure. Comparison of ovarian cupping and fennel infusion with Metformin on oligomenorrhea and ovulation in women with polycystic ovarian syndrome: a clinical trial 2017.	Full text not obtainable.
6.	Scientific Impact Paper No. 13: Metformin Therapy for the Management of Infertility in Women with Polycystic Ovary Syndrome. Obstetrician & Gynaecologist 2017, 19, 339-339, doi:10.1111/tog.12436.	Wrong study design.
7.	Effect of using metformin on the incidence of gestational diabetes and preeclampsia in pregnant women with polycystic ovary. Effect of using metformin versus not using on the incidence of gestational diabetes and preeclampsia in pregnant women with polycystic ovary: A randomized clinical trial 2018.	Wrong publication type
8.	?Effects of myo-inositol on induction of ovulation. Comparison the effects of myo-inositol plus clomiphene citrate with metformin plus clomiphene citrate on induction of ovulation among patients with polycystic ovarian syndrome. 2018.	Wrong publication type
9.	New strategies to lose weight for women with polycystic ovary syndrome. Novel strategies in weight loss in women with polycystic ovary syndrome: does the gut microbiome play a role? 2018.	Wrong publication type
10.	A study to compare the efficacy of two drugs on the success of assisted reproductive therapy in women with polycystic ovarian syndrome and undergoing treatment with IVF. Randomised Control Trial comparing the effects of Metformin to Myoinositol on ART outcome in women with PCOS undergoing IVF cycles 2018.	Wrong publication type

Supplementary File 1

11.	A study to compare the efficacy and adverse effects of metformin versus myoinositol plus d-chiroinositol combination therapy in polycystic ovarian syndrome. A prospective randomised comparative study of metformin versus myoinositol plus d-chiroinositol combination therapy in polycystic ovarian syndrome 2019.	Wrong publication type
12.	Effect of combined electroacupuncture and medical therapy on insulin resistance in polycystic ovary syndrome patients. Combination of electroacupuncture and pharmacological treatment in improving insulin resistance (HOMA-IR) in polycystic ovary syndrome patients: a double-blind randomized clinical trial 2020.	Wrong study design
13.	Study to find Effects of Chandraprabha Vati(Ayurvedic Medicine) in Polycystic Ovarian Syndrome Characterised by Small cysts in ovary with irregular,Scanty menses and excess/unwanted hairs on Face,Thighs,Abdomen etc. â??Randomized controlled clinical trial to study the efficacy OF Chandraprabha vati in PCOS.â?? 2020.	Wrong study design
14.	A clinical trial to study the effect of exercise and metformin on mitochondrial health in patients with polycystic ovarian syndrome (PCOS). To assess the efficacy of moderate-intensity exercise training and metformin on mitophagy and mitochondrial phenotype in patients with polycystic ovarian syndrome (PCOS) 2020.	Wrong publication type
15.	Effect of oral contraceptives on levels of adipokines, and adiposity indices in women with polycystic ovary syndrome. A randomized clinical trial to compare the effectiveness of oral contraceptives containing levonorgestrel, desogestrel, cyproterone acetate, and drospirenone on levels of adipokines, and adiposity indices in women with polycystic ovary syndrome. 2020.	Wrong publication type
16.	Effect of treatment by OCP on infertility in PCOD patients. The randomized, single -blinded clinical trial comparing OCP effect before frozen embryo transfer versus gonadotropin â“ releasing hormone agonist injection on improving the outcome of pregnancy in infertile patients with hyper androgenic poly 2020.	Full text not obtainable
17.	Expression of concern: Comparison of myo-inositol and metformin on mental health parameters and biomarkers of oxidative stress in women with polycystic ovary syndrome: a randomized, double-blind, placebo-controlled trial The effects of fish oil omega-3 fatty acid supplementation on mental health parameters and metabolic status of patients with polycystic ovary syndrome: a randomized, double-blind, placebo-controlled trial. Journal of Psychosomatic Obstetrics & Gynecology 2020, 41, I-I, doi:10.1080/0167482X.2020.1842508	Wrong publication type
18.	Evaluation of therapeutic effects of crocina (saffron tablets) in patients with polycystic ovary syndrome: a randomized double-blind clinical trial. 2021.	Wrong publication type
19.	A clinical study in women suffering from polycystic ovary syndrome (PCOS) to test the drug LPRI-424 (dienogest/ethinyl estradiol) during 9 months of treatment. A multicentre, phase III, double-blind, randomised clinical trial to assess the efficacy and safety of LPRI-424 (dienogest 2.00 mg / ethinyl estradiol 0.02 mg) in the treatment of polycystic ovary syndrome (PCOS) versus placebo during 9 cycles 2021.	Wrong publication type
20.	A clinical trial to study the effect of myoinositol based therapy in combination with metformin as compared to metformin alone in women with polycystic ovarian syndrome. A Randomized Controlled Trial comparing Myoinositol based therapy in combination with Metformin versus Metformin monotherapy on the clinical, metabolic and hormonal parameters in Obese reproductive age women with Polycystic Ovarian Syndrome 2021.	Wrong publication type
21.	Efficacy of very low carbohydrate diet combined with metformin in overweight / obese PCOS patients on changing of clinical phenotype, gut microbiota and plasma metabolome after treatment: a randomized, controlled clinical trial. 2021.	Full text not obtainable
22.	A Phase II, randomised, multi-centric, multi-national clinical trial to evaluate the efficacy, tolerability, and safety of a fixed dose combination of Spironolactone, Pioglitazone & Metformin (SPIOMET) for adolescent girls and young adult women (AYAs) with polycystic ovary syndrome (PCOS). 2021.	Wrong publication type
23.	Investigation on the efficacy and safety of Ceylon cinnamon (Cinammomum zeylanicum) compared to metformin in ameliorating symptoms of Polycystic Ovary Syndrome (PCOS): A randomized controlled trial. Investigation on the Efficacy and Safety of Ceylon Cinnamon (Cinammomum zeylanicum) and Metformin in Ameliorating Polycystic Ovary Syndrome (PCOS): A Randomized Controlled Trial 2022.	Wrong comparator
24.	Abdalla, M.A.; Deshmukh, H.; Atkin, S.; Sathyapalan, T. The potential role of incretin-based therapies for polycystic ovary syndrome: a narrative review of the current evidence. Therapeutic Advances in Endocrinology and Metabolism 2021, 12, doi:https://dx.doi.org/10.1177/2042018821989238.	Wrong study design
25.	Abdalmageed, O.S.; Farghaly, T.A.; Abdelaleem, A.A.; Abdelmagied, A.E.; Ali, M.K.; Abbas, A.M. Impact of Metformin on IVF Outcomes in Overweight and Obese Women With Polycystic Ovary Syndrome: A Randomized Double-Blind Controlled Trial. Reproductive	Wrong intervention

Supplementary File 1

	sciences (Thousand Oaks, Calif.) 2019, 26, 1336-1342, doi:https://dx.doi.org/10.1177/1933719118765985.	
26.	Acmaz, G.; Cınar, L.; Acmaz, B.; Aksoy, H.; Kafadar, Y.T.; Madendag, Y.; Ozdemir, F.; Sahin, E.; Muderris, I. The Effects of Oral Isotretinoin in Women with Acne and Polycystic Ovary Syndrome. BioMed research international 2019, 10.1155/2019/2513067, 1-5, doi:10.1155/2019/2513067.	Wrong study design
27.	Advani, K.; Batra, M.; Tajpuriya, S.; Gupta, R.; Saraswat, A.; Nagar, H.D.; Makwana, L.; Kshirsagar, S.; Kaul, P.; Ghosh, A.K., et al. Efficacy of combination therapy of inositols, antioxidants and vitamins in obese and non-obese women with polycystic ovary syndrome: an observational study. Journal of Obstetrics & Gynaecology 2020, 40, 96-101, doi:10.1080/01443615.2019.1604644.	Wrong study design
28.	Ahc, M. What Are the Roles of the Combined Oral Contraceptive Pill and Metformin in the Management of Polycystic Ovary Syndrome? OB/GYN Clinical Alert 2020, 36, N.PAG-N.PAG.	Wrong study design
29.	Ainehchi, N.; Khaki, A.; Ouladsahebmadarek, E.; Hammadeh, M.; Farzadi, L.; Farshbaf-Khalili, A.; Asnaashari, S.; Khamnei, H.J.; Khaki, A.A.; Shokoohi, M. The effect of clomiphene citrate, herbal mixture, and herbal mixture along with clomiphene citrate on clinical and para-clinical parameters in infertile women with polycystic ovary syndrome: A randomized controlled clinical trial. Archives of Medical Science 2020, 16, 1304-1318, doi:https://dx.doi.org/10.5114/AOMS.2020.93271.	Wrong intervention
30.	Akhtar, T.; Shaikh, F.; Basma; Ahmed, W.U.N.; Lashari, S.; Bhatti, N. Comparison of myoinositol versus combination of metformin and myoinositol in ovulation induction in polycystic ovarian syndrome. Pakistan Journal of Medical and Health Sciences 2021, 15, 1494-1496, doi:http://dx.doi.org/10.53350/pjmhs211561494.	Wrong outcome
31.	Alalami, H.; Sathyapalan, T.; Atkin, S.L. Cardiovascular profile of pharmacological agents used for the management of polycystic ovary syndrome. Therapeutic Advances in Endocrinology and Metabolism 2019, 10, doi:http://dx.doi.org/10.1177/2042018818805674.	Wrong study design
32.	Alalfy, M.; Rashwan, A.S.S.A.; Hussein, M.; Bakry, A.; Eid, A.; Eid, M.M. The Use of N-Acetyl Cysteine Versus Chromium Picolinate as an Adjuvant to Clomiphene Citrate and Metformin in PCOS Women to Improve Ovulation Induction and Insulin Resistance: A Pilot Randomized Controlled Trial. Current Women's Health Reviews 2022, 18, e241221192204, doi:https://dx.doi.org/10.2174/1573404817666210310164353.	Wrong comparator
33.	Alhussain, F.; Alruthia, Y.; Al-Mandeel, H.; Bellahwal, A.; Alharbi, F.; Almogbel, Y.; Awwad, O.; Dala'een, R.; Alharbi, F.A. Metformin improves the depression symptoms of women with polycystic ovary syndrome in a lifestyle modification program. Patient Preference and Adherence 2020, 14, 737-746, doi:http://dx.doi.org/10.2147/PPA.S244273.	Wrong study design
34.	Ali, D.-E.S.; Shah, M.; Ali, A.; Malik, M.O.; Rehman, F.; Badshah, H.; Ehtesham, E.; Vitale, S.G. Treatment with Metformin and Combination of Metformin Plus Pioglitazone on Serum Levels of IL-6 and IL-8 in Polycystic Ovary Syndrome: A Randomized Clinical Trial. Hormone and metabolic research = Hormon- und Stoffwechselforschung = Hormones et metabolisme 2019, 51, 714-722, doi:https://dx.doi.org/10.1055/a-1018-9606.	Full text not obtainable
35.	Almalki, H.H.; Alshibani, T.M.; Alhifany, A.A.; Almohammed, O.A. Comparative efficacy of statins, metformin, spironolactone and combined oral contraceptives in reducing testosterone levels in women with polycystic ovary syndrome: a network meta-analysis of randomized clinical trials. BMC women's health 2020, 20, 1-6, doi:10.1186/s12905-020-00919-5.	Wrong intervention
36.	Amiri, M.; Kabir, A.; Nahidi, F.; Shekofteh, M.; Ramezani Tehrani, F. Effects of combined oral contraceptives on the clinical and biochemical parameters of hyperandrogenism in patients with polycystic ovary syndrome: a systematic review and meta-analysis. European journal of contraception & reproductive health care 2018, 23, 64-77, doi:10.1080/13625187.2018.1435779.	Wrong comparator
37.	Amiri, M.; Nahidi, F.; Yarandi, R.B.; Khalili, D.; Tohidi, M.; Tehrani, F.R. Effects of oral contraceptives on the quality of life of women with polycystic ovary syndrome: a crossover randomized controlled trial. Health & Quality of Life Outcomes 2020, 18, N.PAG-N.PAG, doi:10.1186/s12955-020-01544-4.	Wrong population
38.	Amiri, M.; Ramezani Tehrani, F.; Nahidi, F.; Kabir, A.; Azizi, F.; Carmina, E. Effects of oral contraceptives on metabolic profile in women with polycystic ovary syndrome: A meta-analysis comparing products containing cyproterone acetate with third generation progestins. Metabolism: clinical and experimental 2017, 73, 22-35, doi:https://dx.doi.org/10.1016/j.metabol.2017.05.001.	Wrong comparator
39.	Amiri, M.; Tehrani, F.R.; Nahidi, F.; Kabir, A.; Azizi, F. Comparing the Effects of Combined Oral Contraceptives Containing Progestins With Low Androgenic and Antiandrogenic Activities on the Hypothalamic-Pituitary-Gonadal Axis in Patients With Polycystic Ovary	Wrong comparator

Supplementary File 1

	Syndrome: Systematic Review and Meta-Analysis. Journal of Medical Internet Research 2018, 20, 1-1, doi:10.2196/resprot.9024.	
40.	Amirkhanloo, F.; Esmaeilzadeh, S.; Mirabi, P.; Abedini, A.; Amiri, M.; Saghebi, R.; Golsorkhtabaramiri, M. Comparison of Foeniculum Vulgare versus metformin on insulin resistance and anthropometric indices of women with polycystic ovary, an open-label controlled trial study. Obesity Medicine 2022, 31, 100401, doi:https://dx.doi.org/10.1016/j.obmed.2022.100401.	Wrong comparator
41.	Ammar, I.M.M.; Salem, M.A.A. Amelioration of polycystic ovary syndrome-related disorders by supplementation of thymoquinone and metformin. Middle East Fertility Society Journal 2021, 26, 29, doi:http://dx.doi.org/10.1186/s43043-021-00076-1.	Wrong comparator
42.	Andræ, F.; Abbott, D.; Stridsklev, S.; Schmedes, A.V.; Odsæter, I.H.; Vanky, E.; Salvesen, Ø. Sustained Maternal Hyperandrogenism During PCOS Pregnancy Reduced by Metformin in Non-obese Women Carrying a Male Fetus. Journal of Clinical Endocrinology & Metabolism 2020, 105, 1-9, doi:10.1210/clinem/dgaa605.	Wrong population
43.	Anonymous. Metformin Therapy for the Management of Infertility in Women with Polycystic Ovary Syndrome: Scientific Impact Paper No. 13. BJOG : an international journal of obstetrics and gynaecology 2017, 124, e306-e313, doi:https://dx.doi.org/10.1111/1471-0528.14764.	Wrong study design
44.	Anonymous. Screening and Management of the Hyperandrogenic Adolescent: ACOG Committee Opinion, Number 789. Obstetrics and gynecology 2019, 134, e106-e114, doi:https://dx.doi.org/10.1097/AOG.0000000000003475.	Wrong study design
45.	Armanini, D.; Boscaro, M.; Bordin, L.; Sabbadin, C. Controversies in the Pathogenesis, Diagnosis and Treatment of PCOS: Focus on Insulin Resistance, Inflammation, and Hyperandrogenism. International journal of molecular sciences 2022, 23, doi:https://dx.doi.org/10.3390/ijms23084110.	Wrong study design
46.	Artini, P.G.; Obino, M.E.R.; Sergiampietri, C.; Pinelli, S.; Papini, F.; Casarosa, E.; Cela, V. PCOS and pregnancy: a review of available therapies to improve the outcome of pregnancy in women with polycystic ovary syndrome. Expert review of endocrinology & metabolism 2018, 13, 87-98, doi:https://dx.doi.org/10.1080/17446651.2018.1431122	Wrong study design
47.	Arya, S.; Hansen, K.R.; Wild, R.A. Metformin, rosiglitazone, or both for obese women with polycystic ovary syndrome? Fertility & Sterility 2020, 113, 87-88, doi:10.1016/j.fertnstert.2019.10.006.	Wrong study design
48.	Asanidze, E.; Kristesashvili, J.; Pkhaladze, L.; Khomasuridze, A. The value of anti-Mullerian hormone in the management of polycystic ovary syndrome in adolescents. Gynecological endocrinology : the official journal of the International Society of Gynecological Endocrinology 2019, 35, 974-977, doi:https://dx.doi.org/10.1080/09513590.2019.1616689.	Wrong comparator
49.	Ashok Kumar, M.; Samuel Gideon George, P.; Dasari, A.; Shanmugasundaram, P. A single-blinded randomized trial to evaluate the efficacy of N-acetyl cysteine over metformin in patients with polycystic ovarian syndrome. Drug Invention Today 2018, 10, 241-243.	Wrong comparator
50.	Aversa, A.; La Vignera, S.; Rago, R.; Gambineri, A.; Nappi, R.E.; Calogero, A.E.; Ferlin, A. Fundamental concepts and novel aspects of polycystic ovarian syndrome: Expert consensus resolutions. Frontiers in endocrinology 2020, 11, 516, doi:https://dx.doi.org/10.3389/fendo.2020.00516.	Wrong study design
51.	Azizi Kutenaei, M.; Hosseini Teshnizi, S.; Ghaemmaghami, P.; Eini, F.; Roozbeh, N. The effects of myo-inositol vs. metformin on the ovarian function in the polycystic ovary syndrome: a systematic review and meta-analysis. European review for medical and pharmacological sciences 2021, 25, 3105-3115, doi:https://dx.doi.org/10.26355/eurrev_202104_25565.	Wrong comparator
52.	Bahadur, A.; Yadav, A.; Chaturvedi, J.; Mundhra, R.; Rajput, R.; Naithani, M.; Bhattacharya, N.; Prerna, J.; Kumari, S.; Verma, N., et al. Effect of two different doses of Vitamin D supplementation on clinical, metabolic and hormonal profiles of Insulin-resistant PCOS patients: a Randomized Controlled Trial. Human reproduction (Oxford, England) 2020, 35, i451.	Wrong comparator
53.	Bahman, M.; Hajimehdipoor, H.; Bioos, S.; Hashem-Dabaghian, F.; Afrakhteh, M.; Tansaz, M. Effect of aslagh capsule, a traditional compound herbal product on oligomenorrhea in patients with polycystic ovary syndrome: A three-arm, open-label, randomized, controlled trial. Galen Medical Journal 2019, 8, 1261, doi:http://dx.doi.org/10.31661/gmj.v0i0.1261.	Wrong comparator
54.	Baldani, D.P.; Skrgatic, L.; Ougouag, R.; Kasum, M. The cardiometabolic effect of current management of polycystic ovary syndrome: strategies of prevention and treatment. Gynecological endocrinology : the official journal of the International Society of	Wrong study design

	Gynecological Endocrinology 2018, 34, 87-91, doi:https://dx.doi.org/10.1080/09513590.2017.1381681.	
55.	Bansal, Y.; Sharma, N. Effect of metformin on levels of androgen in obese women having PCOS with & without Mg supplementation: a randomized control trial. Clinica chimica acta 2022, 530, S412, doi:https://doi.org/10.1016/j.cca.2022.04.441.	Wrong publication type
56.	Battaglia, C.; Battaglia, B.; Casadio, P.; Rizzo, R.; Artini, P.G. Metformin metabolic and vascular effects in normal weight hyperinsulinemic polycystic ovary syndrome patients treated with contraceptive vaginal ring. A pilot study. Gynecological endocrinology : the official journal of the International Society of Gynecological Endocrinology 2020, 36, 1062-1069, doi:https://dx.doi.org/10.1080/09513590.2020.1770213.	Wrong comparator
57.	Behboudi-Gandevani, S.; Abtahi, H.; Saadat, N.; Tohidi, M.; Ramezani Tehrani, F. Effect of phlebotomy versus oral contraceptives containing cyproterone acetate on the clinical and biochemical parameters in women with polycystic ovary syndrome: a randomized controlled trial. Journal of ovarian research 2019, 12, 78, doi:https://dx.doi.org/10.1186/s13048-019-0554-9.	Wrong comparator
58.	Bhide, P.; Pundir, J.; Gudi, A.; Shah, A.; Homburg, R.; Acharya, G. The effect of myo-inositol/di-chiro-inositol on markers of ovarian reserve in women with PCOS undergoing IVF/ICSI: A systematic review and meta-analysis. Acta obstetricia et gynecologica Scandinavica 2019, 98, 1235-1244, doi:10.1111/aogs.13625.	Wrong intervention
59.	Bidhendi Yarandi, R.; Behboudi-Gandevani, S.; Amiri, M.; Ramezani Tehrani, F. Metformin therapy before conception versus throughout the pregnancy and risk of gestational diabetes mellitus in women with polycystic ovary syndrome: A systemic review, meta-analysis and meta-regression. Diabetology and Metabolic Syndrome 2019, 11, 58, doi:http://dx.doi.org/10.1186/s13098-019-0453-7.	Wrong outcome
60.	Bjekic-Macut, J.; Vukasin, T.; Velija-Asimi, Z.; Burekovic, A.; Zdravkovic, M.; Andric, Z.; Brankovic, M.; Crevar-Marinovic, S.; Madic, T.; Stanojlovic, O., et al. Polycystic Ovary Syndrome: A Contemporary Clinical Approach. Current pharmaceutical design 2021, 27, 3812-3820, doi:https://dx.doi.org/10.2174/1381612827666210119104721.	Wrong study design
61.	Bordewijk, E.M.; Nahuis, M.; Costello, M.F.; Van der Veen, F.; Tso, L.O.; Mol, B.W.J.; van Wely, M. Metformin during ovulation induction with gonadotrophins followed by timed intercourse or intrauterine insemination for subfertility associated with polycystic ovary syndrome. The Cochrane database of systematic reviews 2017, 1, CD009090, doi:https://dx.doi.org/10.1002/14651858.CD009090.pub2.	Wrong intervention
62.	Boyd, M.; Ziegler, J. Polycystic Ovary Syndrome, Fertility, Diet, and Lifestyle Modifications: A Review of the Current Evidence. Topics in Clinical Nutrition 2019, 34, 14-30, doi:10.1097/TIN.0000000000000161.	Wrong study design
63.	Burgart, J.M. Polycystic Ovary Disease and Obesity: Leptin, Weight-loss Medication, and Bariatric Surgery. Clinical Obstetrics & Gynecology 2021, 64, 90-95, doi:10.1097/GRF.0000000000000599.	Wrong study design
64.	Cai, M.; Zhang, Y.; Qu, S.; Zhang, M. The safety and efficacy of canagliflozin in women with polycystic ovary syndrome: a randomized control trial. Diabetes 2021, 70, doi:https://doi.org/10.2337/db21-132-LB.	Wrong publication type
65.	Campbell, A. What to Know About Metformin. Diabetes Self-Management 2019, 36, 20-21.	Wrong study design
66.	Cantelmi, T.; Lambiase, E.; Unfer, V.R.; Gambioli, R.; Unfer, V. Inositol treatment for psychological symptoms in Polycystic Ovary Syndrome women. European review for medical and pharmacological sciences 2021, 25, 2383-2389, doi:https://dx.doi.org/10.26355/eurrev_202103_25278.	Wrong study design
67.	Cao, Q.; Hu, Y.; Fu, J.; Huang, X.; Wu, L.; Zhang, J.; Huang, W. Gestational metformin administration in women with polycystic ovary syndrome: A systematic review and meta-analysis of randomized control studies. Journal of Obstetrics & Gynaecology Research 2021, 47, 4148-4157, doi:10.1111/jog.15044.	Wrong outcome
68.	Cao, Y.; Chen, H.; Zhao, D.; Zhang, L.; Yu, X.; Zhou, X.; Liu, Z. The efficacy of Tung's acupuncture for sex hormones in polycystic ovary syndrome: A randomized controlled trial. Complementary therapies in medicine 2019, 44, 182-188, doi:10.1016/j.ctim.2019.04.016.	Wrong intervention
69.	Cao, Y.; Zhang, L.; Zhao, D.; Liu, Z. [DONG's extraordinary acupoints for the ovarian function of polycystic ovary syndrome:a randomized controlled pilot trial]. Zhongguo zhen jiu = Chinese acupuncture & moxibustion 2017, 37, 710-714, doi:https://dx.doi.org/10.13703/j.0255-2930.2017.07.007.	Wrong language
70.	Capozzi, A.; Scambia, G.; Lello, S. Polycystic ovary syndrome (PCOS) and adolescence: How can we manage it? European Journal of Obstetrics & Gynecology & Reproductive Biology 2020, 250, 235-240, doi:10.1016/j.ejogrb.2020.04.024	Wrong study design

Supplementary File 1

71.	Cappelli, V.; Musacchio, M.C.; Bulfoni, A.; Morgante, G.; De Leo, V. Natural molecules for the therapy of hyperandrogenism and metabolic disorders in PCOS. European review for medical and pharmacological sciences 2017, 21, 15-29.	Wrong study design
72.	Carmina, E.; Azziz, R.; Bergfeld, W.; Escobar-Morreale, H.F.; Futterweit, W.; Huddleston, H.; Lobo, R.; Olsen, E. Female Pattern Hair Loss and Androgen Excess: A Report From the Multidisciplinary Androgen Excess and PCOS Committee. The Journal of clinical endocrinology and metabolism 2019, 104, 2875-2891, doi:https://dx.doi.org/10.1210/jc.2018-02548.	Wrong study design
73.	Carmina, E.; Dreno, B.; Lucky, W.A.; Agak, W.G.; Dokras, A.; Kim, J.J.; Lobo, R.A.; Tehrani, F.R.; Dumesic, D. Female Adult Acne and Androgen Excess: A Report From the Multidisciplinary Androgen Excess and PCOS Committee. Journal of the Endocrine Society 2022, 6, 1-11, doi:10.1210/jendso/bvac003.	Wrong intervention
74.	Carvalho, L.M.L.; Ferreira, C.N.; Candido, A.L.; Reis, F.M.; Soter, M.O.; Sales, M.F.; Silva, I.F.O.; Nunes, F.F.C.; Gomes, K.B. Metformin reduces total microparticles and microparticles-expressing tissue factor in women with polycystic ovary syndrome. Archives of gynecology and obstetrics 2017, 296, 617-621, doi:https://dx.doi.org/10.1007/s00404-017-4471-0.	Wrong study design
75.	Casey, G. Metformin - for more than just diabetes? Kai Tiaki Nursing New Zealand 2019, 25, 20-20.	Wrong study design
76.	Chatzis, P.; Tziomalos, K.; Pratilas, G.C.; Makris, V.; Sotiriadis, A.; Dinas, K. The Role of Antiobesity Agents in the Management of Polycystic Ovary Syndrome. Folia medica 2018, 60, 512-520, doi:https://dx.doi.org/10.2478/folmed-2018-0036.	Wrong study design
77.	Chen, M.; Yang, P.; Chen, H.; Chen, S.; Ho, H. The efficacy of long-term metformin treatment in women with polycystic ovary syndrome. Fertility & Sterility 2017, 108, e245-e246, doi:10.1016/j.fertnstert.2017.07.738.	Wrong study design
78.	Chen, X.; He, S.; Wang, D. Effects of metformin on body weight in polycystic ovary syndrome patients: model-based meta-analysis. Expert review of clinical pharmacology 2021, 14, 121-130, doi:https://dx.doi.org/10.1080/17512433.2021.1863788.	Wrong study design
79.	hen, Y.; Li, M.; Deng, H.; Wang, S.; Chen, L.; Li, N.; Xu, D.; Wang, Q. Impact of metformin on C-reactive protein levels in women with polycystic ovary syndrome: A meta-analysis. Oncotarget 2017, 8, 35425-35434, doi:http://dx.doi.org/10.18632/oncotarget.16019.	Wrong comparator
80.	Chen, Z.; Tan, J.; Wang, H.; Zheng, B.; Liu, J.; Hao, G.; Guo, Z.; Sun, Z.; Yu, Q. A Randomized Cohort Study: is It Worth the Time to Receive Antiandrogenic Pretreatment Before Ovulation Induction for Women With Polycystic Ovary Syndrome? Frontiers in endocrinology 2022, 13, doi:https://doi.org/10.3389/fendo.2022.813188.	Wrong comparator
81.	Cignarella, A.; Mioni, R.; Sabbadin, C.; Dassie, F.; Parolin, M.; Vettor, R.; Barbot, M.; Scaroni, C. Pharmacological Approaches to Controlling Cardiometabolic Risk in Women with PCOS. International journal of molecular sciences 2020, 21, doi:https://dx.doi.org/10.3390/ijms21249554.	Wrong study design
82.	Condorelli, R.A.; Calogero, A.E.; Di Mauro, M.; Mongioi, L.M.; Cannarella, R.; Rosta, G.; La Vignera, S. Androgen excess and metabolic disorders in women with PCOS: beyond the body mass index. Journal of endocrinological investigation 2018, 41, 383-388, doi:https://dx.doi.org/10.1007/s40618-017-0762-3.	Wrong study design
83.	Costello, M.F.; Misso, M.L.; Balen, A.; Boyle, J.; Devoto, L.; Garad, R.M.; Hart, R.; Johnson, L.; Jordan, C.; Legro, R.S., et al. Evidence summaries and recommendations from the international evidence-based guideline for the assessment and management of polycystic ovary syndrome: Assessment and treatment of infertility. Human Reproduction Open 2019, 2019, hoy021, doi:http://dx.doi.org/10.1093/hropen/hoy021.	Wrong outcome
84.	Costello, M.F.; Misso, M.L.; Balen, A.; Boyle, J.; Devoto, L.; Garad, R.M.; Hart, R.; Johnson, L.; Jordan, C.; Legro, R.S., et al. A brief update on the evidence supporting the treatment of infertility in polycystic ovary syndrome. Australian & New Zealand journal of obstetrics & gynaecology 2019, 59, 867-873, doi:10.1111/ajo.13051.	Wrong study design
85.	Craig, M.; Temples, H.S.; Weir, B. Polycystic Ovarian Syndrome in Adolescents: Early Diagnosis and Intervention. Journal of Pediatric Healthcare 2020, 34, 166-170, doi:10.1016/j.pedhc.2019.11.007.	Wrong study design
86.	rellin, H. What are the most effective oral medications for ovulation induction in women with PCOS? Evidence-Based Practice 2022, 25, 26-27, doi:10.1097/EBP.0000000000001408.	Wrong study design
87.	Crouch, R.; Hamilton, J.; Raymond, T. Is metformin effective for treating infertility associated with PCOS? Evidence-Based Practice 2022, 25, 35-36, doi:10.1097/EBP.0000000000001328.	Wrong study design
88.	Cui, N.; Feng, X.; Zhao, Z.; Zhang, J.; Xu, Y.; Wang, L.; Hao, G. Restored Plasma Anandamide and Endometrial Expression of Fatty Acid Amide Hydrolase in Women With	Wrong study design

Supplementary File 1

	Polycystic Ovary Syndrome by the Combination Use of Diane-35 and Metformin. Clinical therapeutics 2017, 39, 751-758, doi:10.1016/j.clinthera.2017.02.007.	
89.	Daneshjou, D.; Mehranjani, M.S.; Zadehmodarres, S.; Shariatzadeh, S.M.A.; Mofarahe, Z.S. Sitagliptin/metformin improves the fertilization rate and embryo quality in polycystic ovary syndrome patients through increasing the expression of GDF9 and BMP15: a new alternative to metformin (a randomized trial). Journal of reproductive immunology 2022, 150, doi:https://doi.org/10.1016/j.jri.2022.103499.	Wrong intervention
90.	Daneshjou, D.; Soleimani Mehranjani, M.; Zadeh Modarres, S.; Shariatzadeh, M.A. Sitagliptin/Metformin: A New Medical Treatment in Polycystic Ovary Syndrome. Trends in endocrinology and metabolism: TEM 2020, 31, 890-892, doi:https://dx.doi.org/10.1016/j.tem.2020.09.002.	Wrong study design
91.	Daneshjou, D.; Zadeh Modarres, S.; Soleimani Mehranjani, M.; Shariat Zadeh, S.M.A. Comparing the effect of sitagliptin and metformin on the oocyte and embryo quality in classic PCOS patients undergoing ICSI. Irish journal of medical science 2021, 190, 685-692, doi:https://dx.doi.org/10.1007/s11845-020-02320-5.	Wrong intervention
92.	de Medeiros, S.F. Risks, benefits size and clinical implications of combined oral contraceptive use in women with polycystic ovary syndrome. Reproductive biology and endocrinology : RB&E 2017, 15, 93, doi:https://dx.doi.org/10.1186/s12958-017-0313-y.	Wrong study design
93.	de Medeiros, S.F.; Medeiros, M.A.S.d.; Santos, N.d.S.; Barbosa, B.B.; Yamamoto, M.M.W. Combined Oral Contraceptive Effects on Low-Grade Chronic Inflammatory Mediators in Women with Polycystic Ovary Syndrome: A Systematic Review and Meta-Analysis. International Journal of Inflammation 2018, 10.1155/2018/9591509, 1-13, doi:10.1155/2018/9591509.	Wrong comparator
94.	Della Corte, L.; La Rosa, V.L.; Rapisarda, A.M.C.; Valenti, G.; Morra, I.; Boccellino, A.; Zizolfi, B.; Santangelo, F.; de Rosa, N.; Sapia, F., et al. Current evidences and future perspectives on patient-oriented treatments for polycystic ovary syndrome: An overview. Italian Journal of Gynaecology and Obstetrics 2018, 30, 7-20, doi:http://dx.doi.org/10.14660/2385-0868-81.	Wrong study design
95.	Deng, Y.; Wang, Y.-F.; Zhu, S.-Y.; Ma, X.; Xue, W.; Ma, R.-L.; Sun, A.-J. Is There An Advantage of Using Dingkun Pill () alone or in Combination with Diane-35 for Management of Polycystic Ovary Syndrome? A Randomized Controlled Trial. Chinese journal of integrative medicine 2020, 26, 883-889, doi:https://dx.doi.org/10.1007/s11655-020-3097-4.	Wrong comparator
96.	Devi, N.; Boya, C.; Chhabra, M.; Bansal, D. N-acetyl-cysteine as adjuvant therapy in female infertility: a systematic review and meta-analysis. Journal of basic and clinical physiology and pharmacology 2020, 32, 899-910, doi:https://dx.doi.org/10.1515/jbcpp-2020-0107.	Wrong population
97.	Devi, N.; Boya, C.; Chhabra, M.; Bansal, D. N-acetyl-cysteine as adjuvant therapy in female infertility: a systematic review and meta-analysis. Journal of Basic & Clinical Physiology & Pharmacology 2021, 32, 899-910, doi:10.1515/jbcpp-2020-0107.	Wrong intervention
98.	Diaz, M.; Bassols, J.; Lopez-Bermejo, A.; De Zegher, F.; Ibanez, L. Circulating miR-451a: a biomarker to guide diagnosis and treatment of polycystic ovary syndrome in adolescent girls. Hormone research in paediatrics 2019, 91, 117, doi:https://doi.org/10.1159/000501868.	Wrong outcome
99.	Díaz, M.; Bassols, J.; López-Bermejo, A.; de Zegher, F.; Ibáñez, L. Low Circulating Levels of miR-451a in Girls with Polycystic Ovary Syndrome: Different Effects of Randomized Treatments. Journal of Clinical Endocrinology & Metabolism 2019, 10.1210/clinem/dgz204, N.PAG-N.PAG, doi:10.1210/clinem/dgz204.	Wrong outcome
100.	Dm, S.M. Abstract #1184447: effects of vitamin D supplementation on metabolic and endocrine abnormalities in polycystic ovary syndrome. Endocrine practice 2022, 28, S124, doi:https://doi.org/10.1016/j.eprac.2022.03.292.	Wrong publication type
101.	Dodd, J.M.; Grivell, R.M.; Deussen, A.R.; Hague, W.M. Metformin for women who are overweight or obese during pregnancy for improving maternal and infant outcomes. The Cochrane database of systematic reviews 2018, 7, CD010564, doi:https://dx.doi.org/10.1002/14651858.CD010564.pub2.	Wrong population
102.	Doi, S.A.R.; Furuya-Kanamori, L.; Toft, E.; Musa, O.A.H.; Islam, N.; Clark, J.; Thalib, L. Metformin in pregnancy to avert gestational diabetes in women at high risk: Meta-analysis of randomized controlled trials. Obesity reviews : an official journal of the International Association for the Study of Obesity 2020, 21, e12964, doi:https://dx.doi.org/10.1111/obr.12964.	Wrong outcome
103.	Dubois, W.I.L. METFORMIN: THE UNAUTHORIZED BIOGRAPHY. Diabetes Self-Management 2022, 39, 62-67.	Wrong publication type

Supplementary File 1

104. Duguech, L.M.M.; Legro, R.S. Pharmacologic Treatment of Polycystic Ovary Syndrome: Alternate and Future Paths. Seminars in reproductive medicine 2017, 35, 326-343, doi:https://dx.doi.org/10.1055/s-0037-1603729.	Wrong study design
105. Dwivedi, A.N.D.; Ganesh, V.; Shukla, R.C.; Jain, M.; Kumar, I. Colour Doppler evaluation of uterine and ovarian blood flow in patients of polycystic ovarian disease and post-treatment changes. Clinical radiology 2020, 75, 772-779, doi:10.1016/j.crad.2020.05.023.	Wrong outcome
106. El Sharkwy, I.; Sharaf El-Din, M. l-Carnitine plus metformin in clomiphene-resistant obese PCOS women, reproductive and metabolic effects: a randomized clinical trial. Gynecological Endocrinology 2019.	Wrong intervention
107. Elkind-Hirsch, K.E.; Paterson, M.; Seidemann, E.; Gutowski, H. Body mass index does not affect suppression of hyperandrogenism but does impact carbohydrate metabolism during low-dose folate-supplemented ethinyl estradiol/drospirenone oral contraceptive therapy in women with polycystic ovary syndrome. Journal of Reproductive Medicine 2017, 62, 357.	Wrong study design
108. Elvir Zelaya, R.; Carbia, C.D.O.; Chong, A.B.D.O.; Hahn, K.D.O. In women with polycystic ovary syndrome, does pioglitazone decrease testosterone more than metformin? Evidence-Based Practice 2021, 24, 35-38, doi:10.1097/EBP.0000000000000892.	Wrong study design
109. Facchinetti, F.; Appetecchia, M.; Aragona, C.; Bevilacqua, A.; Bezerra Espinola, M.S.; Bizzarri, M.; D'Anna, R.; Dewailly, D.; Diamanti-Kandarakis, E.; Hernandez Marin, I., et al. Experts' opinion on inositols in treating polycystic ovary syndrome and non-insulin dependent diabetes mellitus: a further help for human reproduction and beyond. Expert opinion on drug metabolism & toxicology 2020, 16, 255-274, doi:https://dx.doi.org/10.1080/17425255.2020.1737675.	Wrong study design
110. Facchinetti, F.; Orru, B.; Grandi, G.; Unfer, V. Short-term effects of metformin and myo-inositol in women with polycystic ovarian syndrome (PCOS): a meta-analysis of randomized clinical trials. Gynecological endocrinology : the official journal of the International Society of Gynecological Endocrinology 2019, 35, 198-206, doi:https://dx.doi.org/10.1080/09513590.2018.1540578.	Wrong comparator
111. Fang, F.; Ni, K.; Cai, Y.; Shang, J.; Zhang, X.; Xiong, C. Effect of vitamin D supplementation on polycystic ovary syndrome: A systematic review and meta-analysis of randomized controlled trials. Complementary therapies in clinical practice 2017, 26, 53-60, doi:10.1016/j.ctcp.2016.11.008.	Wrong intervention
112. Farhadian, M.; Barati, S.; Mahmoodi, M.; Barati Mosleh, A.; Yavangui, M. Comparison of green tea and metformin effects on anthropometric indicators in women with polycystic ovarian syndrome: A clinical trial study. Journal of Reports in Pharmaceutical Sciences 2020, 9, 97, doi:https://doi.org/10.4103/jrptps.JRPTPS1419.	Wrong comparator
113. Fatima, A.; Khan, S.A.; Saifuddin, Z.; Aslam, R. Comparison of efficacy of clomiphene citrate alone and with metformin for treatment of infertility in polycystic ovarian syndrome. Rawal Medical Journal 2018, 43, 285-288.	Wrong intervention
114. Ferrer, M.J.; Silva, A.F.; Abruzzese, G.A.; Velazquez, M.E.; Motta, A.B. Lipid Metabolism and Relevant Disorders to Female Reproductive Health. Current medicinal chemistry 2021, 28, 5625-5647, doi:https://dx.doi.org/10.2174/0929867328666210106142912	Wrong study design
115. Figurova, J.; Dravecka, I.; Petrikova, J.; Javorsky, M.; Lazurova, I. The effect of alfacalcidiol and metformin on metabolic disturbances in women with polycystic ovary syndrome. Hormone molecular biology and clinical investigation 2017, 29, 85-91, doi:https://dx.doi.org/10.1515/hmbci-2016-0039.	Wrong comparator
116. Fougner, S.L.; Vanky, E.; Lovvik, T.S.; Carlsen, S.M. No impact of gestational diabetes mellitus on pregnancy complications in women with PCOS, regardless of GDM criteria used. PloS one 2021, 16, e0254895, doi:https://dx.doi.org/10.1371/journal.pone.0254895.	Wrong outcome
117. Fruzzetti, F.; Perini, D.; Russo, M.; Bucci, F.; Gadducci, A. Comparison of two insulin sensitizers, metformin and myo-inositol, in women with polycystic ovary syndrome (PCOS). Gynecological Endocrinology 2017, 33, 39, doi:https://doi.org/10.1080/09513590.2016.1236078.	Wrong comparator
118. Fujita, Y.; Inagaki, N. Metformin: clinical topics and new mechanisms of action. Diabetology International 2017, 8, 4-6, doi:http://dx.doi.org/10.1007/s13340-016-0300-0.	Wrong study design
119. Gadalla, M.A.; Norman, R.J.; Tay, C.T.; Hiam, D.S.; Melder, A.; Pundir, J.; Thangaratinam, S.; Teede, H.J.; Mol, B.W.J.; Moran, L.J. Medical and Surgical Treatment of Reproductive Outcomes in Polycystic Ovary Syndrome: An Overview of Systematic Reviews. International Journal of Fertility & Sterility 2020, 13, 257-270, doi:10.22074/ijfs.2020.5608.	Wrong study design
120. Garad, R.M.; Teede, H.J. Polycystic ovary syndrome: improving policies, awareness, and clinical care. Current Opinion in Endocrine and Metabolic Research 2020, 12, 112-118, doi:http://dx.doi.org/10.1016/j.coemr.2020.04.007.	Wrong study design

Supplementary File 1

121. Garcia-Beltran, C.; Malpique, R.; Carbonetto, B.; González-Torres, P.; Henares, D.; Brotons, P.; Muñoz-Almagro, C.; López-Bermejo, A.; Zegher, F.; Ibáñez, L. Gut microbiota in adolescent girls with polycystic ovary syndrome: Effects of randomized treatments. Pediatric obesity 2021, 16, 1-11, doi:10.1111/ijpo.12734.	Wrong outcome
122. Gariani, K.; Hugon-Rodin, J.; Philippe, J.; Righini, M.; Blondon, M. Association between polycystic ovary syndrome and venous thromboembolism: A systematic review and meta-analysis. Thrombosis research 2020, 185, 102-108, doi:https://dx.doi.org/10.1016/j.thromres.2019.11.019.	Wrong intervention
123. Gateva, A.; Unfer, V.; Kamenov, Z. The use of inositol(s) isomers in the management of polycystic ovary syndrome: a comprehensive review. Gynecological endocrinology : the official journal of the International Society of Gynecological Endocrinology 2018, 34, 545-550, doi:https://dx.doi.org/10.1080/09513590.2017.1421632.	Wrong study design
124. Genazzani, A. Inositols: reflections on how to choose the appropriate one for PCOS. Gynecological endocrinology : the official journal of the International Society of Gynecological Endocrinology 2020, 36, 1045-1046, doi:https://dx.doi.org/10.1080/09513590.2020.1846697	Wrong study design
125. Glintborg, D.; Andersen, M. MANAGEMENT OF ENDOCRINE DISEASE: Morbidity in polycystic ovary syndrome. European journal of endocrinology 2017, 176, R53-R65.	Wrong study design
126. Goldrick, K.M.; Kostroun, K.E.; Mondshine, J.N.; Robinson, R.D.; Mankus, E.B.; Knudtson, J.F. METFORMIN SHOULD BE RECOMMENDED FOR MORE PATIENTS WITH PCOS BASED ON UPDATED GUIDELINES. Fertility & Sterility 2020, 114, e404-e405, doi:10.1016/j.fertnstert.2020.08.1184.	Wrong publication type
127. Gong, W.; Mi, Y.; Shi, Y. The effect of modified erchen decoction on reproductive endocrine functions and glucose metabolism in patients with phlegm-dampness polycystic ovary syndrome complicated with insulin resistance. International Journal of Clinical and Experimental Medicine 2020, 13, 5932-5940.	Wrong intervention
128. Gordon-Elliott, J.S.; Ernst, C.L.; Fersh, M.E.; Albertini, E.; Lusskin, S.I.; Altemus, M. The Hypothalamic-Pituitary-Gonadal Axis and Women's Mental Health: PCOS, Premenstrual Dysphoric Disorder, and Perimenopause. Psychiatric Times 2017, 34, 5-8.	Wrong study design
129. Goyal, M.; Dawood, A. Debates regarding lean patients with polycystic ovary syndrome: A narrative review. Journal of Human Reproductive Sciences 2017, 10, 154-161, doi:http://dx.doi.org/10.4103/jhrs.JHRS_77_17.	Wrong study design
130. Greenhill, C. PCOS: Metformin risk for offspring. Nature Reviews Endocrinology 2018, 14, 253-253, doi:10.1038/nrendo.2018.34.	Wrong publication type
131. Grindheim, S.; Ebbing, C.; Karlsen, H.O.; Skulstad, S.M.; Real, F.G.; Lonnebotn, M.; Lovvik, T.; Vanky, E.; Kessler, J. Metformin exposure, maternal PCOS status and fetal venous liver circulation: A randomized, placebo-controlled study. PloS one 2022, 17, e0262987, doi:https://dx.doi.org/10.1371/journal.pone.0262987.	Wrong population
132. Guan, C.; Zahid, S.; Minhas, A.S.; Ouyang, P.; Vaught, A.; Baker, V.L.; Michos, E.D. Polycystic ovary syndrome: a "risk-enhancing" factor for cardiovascular disease. Fertility & Sterility 2022, 117, 924-935, doi:10.1016/j.fertnstert.2022.03.009.	Wrong study design
133. Gunalan, E.; Yaba, A.; Yilmaz, B. The effect of nutrient supplementation in the management of polycystic ovary syndrome-associated metabolic dysfunctions: A critical review. Journal of the Turkish German Gynecology Association 2018, 19, 220-232, doi:http://dx.doi.org/10.4274/jtgga.2018.0077.	Wrong study design
134. Haas, J.; Bentov, Y. Should metformin be included in fertility treatment of PCOS patients? Medical hypotheses 2017, 100, 54-58, doi:10.1016/j.mehy.2017.01.012.	Wrong study design
135. Hakimi, O.; Cameron, L.-C. Effect of Exercise on Ovulation: A Systematic Review. Sports Medicine 2017, 47, 1555-1567, doi:10.1007/s40279-016-0669-8.	Wrong population
136. Hameed, L.; Farooq, A.D.; Qureshi, T. Analysis of Unani coded formulation on the hormonal parameters of patients with polycystic ovarian syndrome. Pakistan journal of pharmaceutical sciences 2021, 34, 899-907	Wrong intervention
137. Hanem, L.G.E.; Stridsklev, S.; Juliusson, P.B.; Roelants, M.; Carlsen, S.M.; Odegard, R.; Vanky, E. Intrauterine metformin exposure influences offspring growth,-a 4-year follow-up of children born to mothers with polycystic ovary syndrome. Endocrine reviews 2017, 38.	Wrong publication type
138. Hanem, L.G.E.; Stridsklev, S.; Juliusson, P.B.; Salvesen, O.; Roelants, M.; Carlsen, S.M.; Odegard, R.; Vanky, E. Metformin Use in PCOS Pregnancies Increases the Risk of Offspring Overweight at 4 Years of Age: Follow-Up of Two RCTs. The Journal of clinical endocrinology and metabolism 2018, 103, 1612-1621, doi:https://dx.doi.org/10.1210/jc.2017-02419.	Wrong outcome

Supplementary File 1

139. Hashim, H.A.; Shokeir, T.; Badawy, A. RETRACTED: Letrozole versus combined metformin and clomiphene citrate for ovulation induction in clomiphene-resistant women with polycystic ovary syndrome: a randomized controlled trial. Elsevier B.V.: New York, New York, 2020; Vol. 114, pp 667-667.	Wrong publication type
140. Hefny, M.; Mohammad, M.; Wafa, Y. Effect of N-acetylcysteine as an adjuvant to clomiphene citrate for induction of ovulation in patients with polycystic ovary syndrome. BJOG 2018, 125, 38, doi:https://doi.org/10.1111/1471-0528.15132.	Wrong intervention
141. Heidari, B.; Change, A.Y.; Lerman, L.O.; Lerman, A. Effect of metformin on microvascular endothelial function in polycystic ovary syndrome. Circulation 2018, 138.	Wrong publication type
142. Heidari, B.; Change, A.Y.; Lerman, L.O.; Lerman, A. Abstract 12145: Effect of Metformin on Microvascular Endothelial Function in Polycystic Ovary Syndrome. Circulation 2018, 138, A12145-A12145.	Wrong publication type
143. Helvaci, N.; Yildiz, B.O. Polycystic ovary syndrome and aging: Health implications after menopause. Maturitas 2020, 139, 12-19, doi:10.1016/j.maturitas.2020.05.013.	Wrong study design
144. Hibberd, R.; Raine-Fenning, N.; Thornton, J. Re: Inositol treatment of anovulation in women with polycystic ovary syndrome: a meta-analysis of randomised trials. Wiley-Blackwell: Malden, Massachusetts, 2018; Vol. 125, pp 509-509.	Wrong intervention
145. Hjorth-Hansen, A.; Eggebo, T.; Salvesen, K.A.; Vanky, E.; Odegard, R. Head size and growth in utero and at birth in metformin exposed children born to mothers with PCOS-a randomized controlled trial. Endocrine reviews 2017, 38.	Wrong publication type
146. Hjorth-Hansen, A.; Salvesen, Ø.; Engen Hanem, L.G.; Eggebø, T.; Salvesen, K.Å.; Vanky, E.; Ødegård, R. Fetal Growth and Birth Anthropometrics in Metformin-Exposed Offspring Born to Mothers With PCOS. Journal of Clinical Endocrinology & Metabolism 2017, 10.1210/jc.2017-01191, N.PAG-N.PAG, doi:10.1210/jc.2017-01191	Wrong outcome
147. Hjorth-Hansen, A.; Salvesen, O.; Engen Hanem, L.G.; Eggebo, T.; Salvesen, K.A.; Vanky, E.; Odegard, R. Fetal Growth and Birth Anthropometrics in Metformin-Exposed Offspring Born to Mothers With PCOS. The Journal of clinical endocrinology and metabolism 2018, 103, 740-747, doi:https://dx.doi.org/10.1210/jc.2017-01191.	Wrong outcome
148. Hu, A.C.; Chapman, L.W.; Mesinkovska, N.A. The efficacy and use of finasteride in women: a systematic review. International journal of dermatology 2019, 58, 759-776, doi:https://dx.doi.org/10.1111/ijd.14370.	Wrong population
149. Huang, C.D.O. Is metformin and spironolactone better than metformin alone for improving polycystic ovarian syndrome symptoms? Evidence-Based Practice 2019, 22, 31-32, doi:10.1097/EBP.0000000000000207.	Wrong study design
150. Huddleston, H.G.; Dokras, A. Diagnosis and Treatment of Polycystic Ovary Syndrome. JAMA: Journal of the American Medical Association 2022, 327, 274-275, doi:10.1001/jama.2021.23769.	Wrong study design
151. Ibanez, L.; Del Rio, L.; Diaz, M.; Sebastiani, G.; Pozo, O.J.; Lopez-Bermejo, A.; De Zegher, F.E. Ovulation rates after randomized interventions for polycystic ovary syndrome in adolescent girls. Endocrine reviews 2017, 38.	Wrong publication type
152. Ibanez, L.; Oberfield, S.E.; Witchel, S.; Auchus, R.J.; Chang, R.J.; Codner, E.; Dabadghao, P.; Darendeliler, F.; Elbarbary, N.S.; Gambineri, A., et al. An International Consortium Update: Pathophysiology, Diagnosis, and Treatment of Polycystic Ovarian Syndrome in Adolescence. Hormone research in paediatrics 2017, 88, 371-395, doi:https://dx.doi.org/10.1159/000479371.	Wrong study design
153. Ibáñez, L.; Zegher, F. Polycystic ovary syndrome in adolescent girls. Pediatric obesity 2020, 15, N.PAG-N.PAG, doi:10.1111/ijpo.12586.	Wrong study design
154. Iervolino, M.; Lepore, E.; Forte, G.; Laganà, A.S.; Buzzaccarini, G.; Unfer, V. Natural Molecules in the Management of Polycystic Ovary Syndrome (PCOS): An Analytical Review. Nutrients 2021, 13, 1677, doi:10.3390/nu13051677.	Wrong study design
155. Jam Ashkezari, S.; Namiranian, N.; Gholami, S.; Elahi, M.; Rahmanian, M. Evaluation of the metformin effects on Anti-Mullerian Hormone in women with polycystic ovarian syndrome: a double-blind randomized clinical trial. International Journal of Reproductive BioMedicine 2019, 17, 143, doi:https://doi.org/10.18502/8.	Wrong publication type
156. Jamilian, H.; Jamilian, M.; Foroozanfard, F.; Afshar Ebrahimi, F.; Bahmani, F.; Asemi, Z. Comparison of myo-inositol and metformin on mental health parameters and biomarkers of oxidative stress in women with polycystic ovary syndrome: a randomized, double-blind, placebo-controlled trial. Journal of Psychosomatic Obstetrics & Gynecology 2018, 39, 307-314, doi:10.1080/0167482X.2017.1383381.	Wrong comparator

Supplementary File 1

157. Jamilian, M.; Farhat, P.; Foroozanfard, F.; Afshar Ebrahimi, F.; Aghadavod, E.; Bahmani, F.; Badehnoosh, B.; Jamilian, H.; Asemi, Z. Comparison of myo-inositol and metformin on clinical, metabolic and genetic parameters in polycystic ovary syndrome: a randomized controlled clinical trial. Clinical endocrinology 2017, 87, 194, doi:https://doi.org/10.1111/cen.13366.	Wrong comparator
158. Janati, S.; Behmanesh, M.A.; Najafzadehvarzi, H.; Kassani, A.; Athari, N.; Poormoosavi, S.M. Changes of Serum Level of Homocysteine and Oxidative Stress Markers by Metformin and Inositol in Infertile Women with Polycystic Ovary Syndrome: a Double Blind Randomized Clinical Trial Study. International Journal of Fertility and Sterility 2022, 16, 102, doi:https://doi.org/10.22074/IJFS.2021.530040.1125.	Wrong comparator
159. Janez, A.; Salamun, V.; Jensterle, M.; Bokal, E.V. Short-term intervention with liraglutide and metformin increased fertility potential in a subset of obese PCOS proceeding in vitro sterilisation. Diabetes 2017, 66, A561.	Wrong publication type
160. Javed, Z.; Papageorgiou, M.; Madden, L.A.; Rigby, A.S.; Kilpatrick, E.S.; Atkin, S.L.; Sathyapalan, T. The effects of empagliflozin vs metformin on endothelial microparticles in overweight/obese women with polycystic ovary syndrome. Endocrine Connections 2020, 9, 563-569, doi:http://dx.doi.org/10.1530/EC-20-0173.	Wrong outcome
161. Jazani, A.M.; Nazemiyeh, H.; Tansaz, M.; Bazargani, H.S.; Fazljou, S.M.B.; Azgomi, R.N.D.; Hamdi, K. Celery plus anise versus metformin for treatment of oligomenorrhea in polycystic ovary syndrome: a triple-blind, randomized clinical trial. Iranian Red Crescent Medical Journal 2018, 20, doi:https://doi.org/10.5812/ircmj.67181.	Wrong comparator
162. Jensterle, M.; Ferjan, S.; Janez, A. DPP4 inhibitor sitagliptin in combination with metformin prevent weight regain in obese women with pcos previously treated with liraglutide. Endocrine reviews 2017, 38.	Wrong publication type
163. Jensterle, M.; Goricar, K.; Janez, A. Add on DPP-4 inhibitor alogliptin alone or in combination with pioglitazone improved beta-cell function and insulin sensitivity in metformin treated PCOS. Endocrine research 2017, https://doi.org/10.1080/07435800.2017.1294602, 1, doi:https://doi.org/10.1080/07435800.2017.1294602.	Wrong intervention
164. Jensterle, M.; Salamun, V.; Bokal, E.V.; Janez, A. Short-term intervention with liraglutide and metformin increased fertility potential in a subset of obese women with PCOS proceeding in vitro fertilization. Endocrine reviews 2017, 38.	Wrong publication type
165. Jiang, J.; Gao, S.; Zhang, Y. Therapeutic effects of dimethyldiguanide combined with clomifene citrate in the treatment of polycystic ovary syndrome. Revista da Associacao Medica Brasileira (1992) 2019, 65, 1144-1150, doi:https://dx.doi.org/10.1590/1806-9282.65.9.1144.	Wrong intervention
166. Jiang, Q.; Shi, Y. Effect of orlistat on obese women with polycystic ovary syndrome. Journal of Bio-X Research 2018, 1, 128-131, doi:https://dx.doi.org/10.1097/JBR.0000000000000017.	Wrong study design
167. Jiang, S.; Tang, T.; Sheng, Y.; Li, R.; Xu, H. The Effects of Letrozole and Metformin Combined with Targeted Nursing Care on Ovarian Function, LH, and FSH in Infertile Patients with Polycystic Ovary Syndrome. Journal of healthcare engineering 2022, 2022, 3712166, doi:https://dx.doi.org/10.1155/2022/3712166.	Wrong study design
168. Jin, P.; Xie, Y. Treatment strategies for women with polycystic ovary syndrome. Gynecological endocrinology : the official journal of the International Society of Gynecological Endocrinology 2018, 34, 272-277, doi:https://dx.doi.org/10.1080/09513590.2017.1395841.	Wrong study design
169. Kachhawa, G.; Senthil Kumar, K.V.; Kulshrestha, V.; Khadgawat, R.; Mahey, R.; Bhatla, N. Efficacy of myo-inositol and d-chiro-inositol combination on menstrual cycle regulation and improving insulin resistance in young women with polycystic ovary syndrome: a randomized open-label study. International journal of gynaecology and obstetrics 2021, https://doi.org/10.1002/ijgo.13971, doi:https://doi.org/10.1002/ijgo.13971.	Wrong comparator
170. Kamboj, M.K.; Bonny, A.E. Polycystic ovary syndrome in adolescence: Diagnostic and therapeutic strategies. Translational Pediatrics 2017, 6, 248-255, doi:http://dx.doi.org/10.21037/tp.2017.09.11.	Wrong study design
171. Kamenov, Z.; Gateva, A. Inositols in PCOS. Molecules (Basel, Switzerland) 2020, 25, doi:https://dx.doi.org/10.3390/molecules25235566.	Wrong study design
172. Kancherla, H.; Konduri, G.; Gelly, R.B.; Tadikonda, R.R. Diagnosis and Treatment of Polycystic Ovary Syndrome (PCOS)-A Comparative Review. International Journal of Pharmaceutical Sciences Review and Research 2022, 73, 107-113, doi:https://dx.doi.org/10.47583/ijpsrr.2022.v73i01.018.	Wrong study design

Supplementary File 1

173. Kataoka, J.; Tassone, E.C.; Misso, M.; Joham, A.E.; Stener-Victorin, E.; Teede, H.; Moran, L.J. Weight Management Interventions in Women with and without PCOS: A Systematic Review. Nutrients 2017, 9, 996, doi:10.3390/nu9090996.	Wrong intervention
174. Kaur, M.D.O.; Silva, B.D.O.; Retailliau, L.D.O. Which treatments are effective at achieving weight loss among overweight or obese reproductive age women with polycystic ovary syndrome? Evidence-Based Practice 2022, 25, 35-36, doi:10.1097/EBP.0000000000001431.	Wrong study design
175. Khan, A.A.; Begum, W. Efficacy of Darchini in the management of polycystic ovarian syndrome: A randomized clinical study. Journal of Herbal Medicine 2019, 15, 100249, doi:http://dx.doi.org/10.1016/j.hermed.2018.11.005.	Wrong comparator
176. Khan, L. Polycystic Ovarian Syndrome in Adolescents: Keys to Diagnosis and Management. Pediatric annals 2021, 50, e272-e275, doi:10.3928/19382359-20210622-01.	Wrong study design
177. Kialka, M.; Galuszka-Bednarczyk, A.; Wajda, A.; Czekanska, P.; Zdzierak, B.; Mrozinska, S.; Janeczko, M.; Milewicz, T. Metformin and changes in serum lipid profile in lean patients with polycystic ovary syndrome. Przeglad lekarski 2017, 74, 144-146.	Full text not obtainable
178. Kim, H.H. Androgens, estrogens, and cardiovascular disease: considerations for women with polycystic ovary syndrome. Fertility & Sterility 2019, 112, 478-479, doi:10.1016/j.fertnstert.2019.06.025.	Wrong study design
179. Kim, Y.; Yoon, S.; Ku, S.; Lee, S.; Shin, J.; Kim, T.; Hur, J. Effect of oral contraceptives over 1-year on change in body composition profiles of women with polycystic ovary syndrome: a cohort study. Fertility & Sterility 2017, 108, e248-e248, doi:10.1016/j.fertnstert.2017.07.745.	Wrong study design
180. Kini, S.; Ramalingam, M. Hirsutism. Obstetrics, Gynaecology and Reproductive Medicine 2018, 28, 129-135, doi:http://dx.doi.org/10.1016/j.ogrm.2018.03.004.	Wrong study design
181. Kolivand, M.; Keramat, A.; Khosravi, A. The Effect of Herbal Teas on Management of Polycystic Ovary Syndrome: A Systematic Review. Journal of Midwifery & Reproductive Health 2017, 5, 1098-1106, doi:10.22038/JMRH.2017.9368.	Wrong intervention
182. Kostopoulou, E.; Anagnostis, P.; Bosdou, J.K.; Spiliotis, B.E.; Goulis, D.G. Polycystic ovary Syndrome in Adolescents: Pitfalls in Diagnosis and Management. Current obesity reports 2020, 9, 193-203, doi:https://dx.doi.org/10.1007/s13679-020-00388-9.	Wrong study design
183. Kriedt, K.J.; Alchami, A.; Davies, M.C. PCOS: diagnosis and management of related infertility. Obstetrics, Gynaecology & Reproductive Medicine 2019, 29, 1-5, doi:10.1016/j.ogrm.2018.12.001.	Wrong study design
184. Krysiak, R.; Gilowska, M.; Okopien, B. The effect of oral contraception on cardiometabolic risk factors in women with elevated androgen levels. Pharmacological reports : PR 2017, 69, 45-49, doi:https://dx.doi.org/10.1016/j.pharep.2016.09.013.	Full text not obtainable
185. Kulkarni, D.; Pai, S.; Ayyar, V.; Bantwal, G.; George, B.; Appaiah, S. Effect of metformin and Vitamin E compared to lifestyle modification on AST/Platelet ratio in PCOS patients with associated NASH. Indian Journal of Endocrinology and Metabolism 2022, 26, S36.	Wrong publication type
186. Kwon, C.-Y.; Lee, B.; Park, K.S. Oriental herbal medicine and moxibustion for polycystic ovary syndrome: A meta-analysis. Medicine 2018, 97, e12942-e12942, doi:10.1097/MD.0000000000012942.	Wrong intervention
187. Lagana, A.S.; Rossetti, P.; Sapia, F.; Chiofalo, B.; Buscema, M.; Valenti, G.; Rapisarda, A.M.C.; Vitale, S.G. Evidence-based and patient-oriented inositol treatment in polycystic ovary syndrome: Changing the perspective of the disease. International Journal of Endocrinology and Metabolism 2017, 15, e43695, doi:http://dx.doi.org/10.5812/ijem.43695.	Wrong study design
188. Laganà, A.S.; Vitagliano, A.; Noventa, M.; Ambrosini, G.; D’Anna, R.; D'Anna, R. Myo-inositol supplementation reduces the amount of gonadotropins and length of ovarian stimulation in women undergoing IVF: a systematic review and meta-analysis of randomized controlled trials. Archives of Gynecology & Obstetrics 2018, 298, 675-684, doi:10.1007/s00404-018-4861-y.	Wrong intervention
189. Lamos, E.M.; Malek, R.; Davis, S.N. GLP-1 receptor agonists in the treatment of polycystic ovary syndrome. Expert review of clinical pharmacology 2017, 10, 401-408, doi:https://dx.doi.org/10.1080/17512433.2017.1292125.	Wrong study design
190. Lazaridou, S.; Dinas, K.; Tziomalos, K. Prevalence, pathogenesis and management of prediabetes and type 2 diabetes mellitus in patients with polycystic ovary syndrome. Hormones (Athens, Greece) 2017, 16, 373-380, doi:https://dx.doi.org/10.14310/horm.2002.1757.	Wrong study design
191. Le, T.N.; Wickham, E.P.R.; Nestler, J.E. Insulin sensitizers in adolescents with polycystic ovary syndrome. Minerva pediatrica 2017, 69, 434-443, doi:https://dx.doi.org/10.23736/S0026-4946.17.04976-3.	Wrong study design

Supplementary File 1

192. Lepine, S.; Jo, J.; Metwally, M.; Cheong, Y.C. Ovarian surgery for symptom relief in women with polycystic ovary syndrome. The Cochrane database of systematic reviews 2017, 11, CD009526, doi:https://dx.doi.org/10.1002/14651858.CD009526.pub2.	Wrong comparator
193. Levin, G.; Rottenstreich, A. Inositol for women with polycystic ovary syndrome-possibly just better than placebo. Wiley-Blackwell: Malden, Massachusetts, 2019; Vol. 98, pp 262-262.	Wrong study design
194. Li, M.F.; Zhou, X.M.; Li, X.L. The Effect of Berberine on Polycystic Ovary Syndrome Patients with Insulin Resistance (PCOS-IR): A Meta-Analysis and Systematic Review. Evidence-based Complementary and Alternative Medicine 2018, 2018, 2532935, doi:http://dx.doi.org/10.1155/2018/2532935.	Wrong comparator
195. Li, R.; Zheng, S.; Mai, T.; Xue, J.; Zhang, Y. Comparison of the effects of metformin and exenatide on pregnancy rate and outcomes in overweight or obese pcos women. Diabetes 2020, 69, doi:https://doi.org/10.2337/db20-1341-P.	Wrong outcome
196. Li, S.; Wang, Y.; Cai, J.; Liu, W.; Yin, H.; Tao, T. Lifestyle intervention, metformin, and acarbose treatments differentially impact liver fat content, serum lipids, and hormone profiles in obese polycystic ovary syndrome patients with impaired glucose tolerance. Diabetes 2020, 69, doi:https://doi.org/10.2337/db20-2013-P.	Wrong outcome
197. Li, X.; Celotto, S.; Pizzol, D.; Gasevic, D.; Ji, M.-M.; Barnini, T.; Solmi, M.; Stubbs, B.; Smith, L.; Lopez Sanchez, G.F., et al. Metformin and health outcomes: An umbrella review of systematic reviews with meta-analyses. European journal of clinical investigation 2021, 51, e13536, doi:https://dx.doi.org/10.1111/eci.13536.	Wrong population
198. Li, Y.; Chen, C.; Ma, Y.; Xiao, J.; Luo, G.; Li, Y.; Wu, D. Multi-system reproductive metabolic disorder: significance for the pathogenesis and therapy of polycystic ovary syndrome (PCOS). Life sciences 2019, 228, 167-175, doi:https://dx.doi.org/10.1016/j.lfs.2019.04.046.	Wrong study design
199. Lim, C.E.D.; Ng, R.W.C.; Cheng, N.C.L.; Zhang, G.S.; Chen, H. Acupuncture for polycystic ovarian syndrome. The Cochrane database of systematic reviews 2019, 7, CD007689, doi:https://dx.doi.org/10.1002/14651858.CD007689.pub4.	Wrong intervention
200. Lim, S.S.; Hutchison, S.K.; Van Ryswyk, E.; Norman, R.J.; Teede, H.J.; Moran, L.J. Lifestyle changes in women with polycystic ovary syndrome. The Cochrane database of systematic reviews 2019, 3, CD007506, doi:https://dx.doi.org/10.1002/14651858.CD007506.pub4.	Wrong intervention
201. Lin, L.; Wang, F.; Chen, M.X.; Mo, Z.W.; Fang, T.Y.; Quan, H.B. [Pulse administration of gonadotropin-releasing hormone combined with metformin for fertility in a non-obese woman with polycystic ovary syndrome]. Zhonghua nei ke za zhi 2019, 58, 531-533, doi:https://dx.doi.org/10.3760/cma.j.issn.0578-1426.2019.07.009.	Full text not obtainable
202. Lin, W.; Feng, J.; Zhou, H.; Chen, X.; Diao, W.; Ma, P. Therapeutic efficacy of clomiphene citrate combined with metformin in patients with polycystic ovary syndrome. Journal of Clinical Pharmacy & Therapeutics 2022, 47, 321-329, doi:10.1111/jcpt.13561.	Wrong outcome
203. Liu, C.; Feng, G.; Huang, W.; Wang, Q.; Yang, S.; Tan, J.; Fu, J.; Liu, D. Comparison of clomiphene citrate and letrozole for ovulation induction in women with polycystic ovary syndrome: a prospective randomized trial. Gynecological endocrinology : the official journal of the International Society of Gynecological Endocrinology 2017, 33, 872-876, doi:https://dx.doi.org/10.1080/09513590.2017.1332174.	Wrong intervention
204. Liu, R.; Li, M.; Wang, P.; Yu, M.; Wang, Z.; Zhang, G.Z. Preventive online and offline health management intervention in polycystic ovary syndrome. World Journal of Clinical Cases 2022, 10, 3060-3068, doi:https://dx.doi.org/10.12998/wjcc.v10.i10.3060.	Wrong intervention
205. Liu, R.-B.; Liu, Y.; Lv, L.-Q.; Xiao, W.; Gong, C.; Yue, J.-X. Effects of Metformin Treatment on Soluble Leptin Receptor Levels in Women with Polycystic Ovary Syndrome. Current medical science 2019, 39, 609-614, doi:https://dx.doi.org/10.1007/s11596-019-2081-8.	Wrong study design
206. Livadas, S.; Anagnostis, P.; Bosdou, J.K.; Bantouna, D.; Paparodis, R. Polycystic ovary syndrome and type 2 diabetes mellitus: A state-ofthe- art review. World Journal of Diabetes 2022, 13, 5-26, doi:https://dx.doi.org/10.4239/wjd.v13.i1.5.	Wrong study design
207. Lovvik, T.S.; Carlsen, S.M.; Salvesen, O.; Steffensen, B.; Bixo, M.; Gomez-Real, F.; Lonnebotn, M.; Hestvold, K.V.; Zabielska, R.; Hirschberg, A.L., et al. Use of metformin to treat pregnant women with polycystic ovary syndrome (PregMet2): a randomised, double-blind, placebo-controlled trial. The lancet. Diabetes & endocrinology 2019, 7, 256-266, doi:https://dx.doi.org/10.1016/S2213-8587(19)30002-6.	Wrong population
208. Luque-Ramirez, M.; Ortiz-Flores, A.E.; Nattero-Chavez, L.; Escobar-Morreale, H.F. A safety evaluation of current medications for adult women with the polycystic ovarian syndrome not pursuing pregnancy. Expert opinion on drug safety 2020, 19, 1559-1576, doi:https://dx.doi.org/10.1080/14740338.2020.1839409.	Wrong study design

Supplementary File 1

209. Macut, D.; Bjekić-Macut, J.; Rahelić, D.; Doknić, M. Insulin and the polycystic ovary syndrome. Diabetes Research & Clinical Practice 2017, 130, 163-170, doi:10.1016/j.diabres.2017.06.011.	Wrong study design
210. Magzoub, R.; Kheirelseid, E.A.H.; Perks, C.; Lewis, S. Does metformin improve reproduction outcomes for non-obese, infertile women with polycystic ovary syndrome? Meta-analysis and systematic review. European Journal of Obstetrics & Gynecology & Reproductive Biology 2022, 269, 38-62, doi:10.1016/j.ejogrb.2022.01.025.	Wrong outcome
211. Mahmood, S.; Answer, S. Metformin and pioglitazone comparison for ovulation induction in PCOS. BJOG 2021, 128, 230, doi:https://doi.org/10.1111/1471-0528.18-16715.	Wrong publication type
212. Makaya, T.; Basu, S.; Poole, R. Management of teenagers with polycystic ovarian syndrome. Paediatrics & Child Health 2019, 29, 303-308, doi:10.1016/j.paed.2019.04.004.	Wrong study design
213. Maleki, V.; Izadi, A.; Farsad-Naeimi, A.; Alizadeh, M. Chromium supplementation does not improve weight loss or metabolic and hormonal variables in patients with polycystic ovary syndrome: A systematic review. Nutrition Research 2018, 56, 1-10, doi:10.1016/j.nutres.2018.04.003.	Wrong intervention
214. Malhotra, N.; Mahey, R.; Agarwal, A.; Rajasekaran, K.; Gupta, M. Short term effects of metformin, myo-inositol or combination on metabolic and endocrine profile of infertile women with polycystic ovarian syndrome (PCOS). Human reproduction (Oxford, England) 2019, 34, i421.	Wrong publication type
215. Manzoor, S.; Ganie, M.A.; Amin, S.; Shah, Z.A.; Bhat, I.A.; Yousuf, S.D.; Jeelani, H.; Kawa, I.A.; Fatima, Q.; Rashid, F. Oral contraceptive use increases risk of inflammatory and coagulatory disorders in women with Polycystic Ovarian Syndrome: An observational study. Scientific reports 2019, 9, 10182, doi:https://dx.doi.org/10.1038/s41598-019-46644-4.	Wrong study design
216. Manzoor, S.; Ganie, M.A.; Majid, S.; Shabir, I.; Kawa, I.A.; Fatima, Q.; Jeelani, H.; Yousuf, S.D.; Rashid, F. Analysis of Intrinsic and Extrinsic Coagulation Pathway Factors in OCP Treated PCOS Women. Indian Journal of Clinical Biochemistry 2021, 36, 278-287, doi:http://dx.doi.org/10.1007/s12291-020-00901-w.	Wrong study design
217. Marciniak, A.; Lejman-Larysz, K.; Nawrocka-Rutkowska, J.; Brodowska, A.; Songin, D. [Polycystic ovary syndrome - current state of knowledge]. Zespol policystycznych jajnikow - aktualny stan wiedzy. 2018, 44, 296-301.	Wrong language
218. Markowicz-Piasecka, M.; Huttunen, K.M.; Mateusiak, L.; Mikiciuk-Olasik, E.; Sikora, J. Is Metformin a Perfect Drug? Updates in Pharmacokinetics and Pharmacodynamics. Current pharmaceutical design 2017, 23, 2532-2550, doi:https://dx.doi.org/10.2174/1381612822666161201152941.	Wrong study design
219. Mascarenhas, M.; Balen, A.H. Treatment update for anovulation and subfertility in polycystic ovary syndrome. Current Opinion in Endocrine and Metabolic Research 2020, 12, 53-58, doi:http://dx.doi.org/10.1016/j.coemr.2020.03.003.	Wrong study design
220. Matrood, R.H.; Abdulhussain, A.S. The added effect of cabergoline to metformin on serum hormones and rate and regularity of menstruation in women with polycystic ovary syndrome. International Journal of Research in Pharmaceutical Sciences 2018, 9, 243-248, doi:http://dx.doi.org/10.26452/ijrps.v9i1.1255.	Full text not obtainable
221. Maysara, A.M.; Nassar, A.T.; Jubran, H.K. The effect of correction of serum level of vitamin D on hyperandrogenism in women with polycystic ovary syndrome and hypovitaminosis D. Clinical and experimental obstetrics & gynecology 2020, 47, 272, doi:https://doi.org/10.31083/j.ceog.2020.02.5248.	Wrong comparator
222. McLean, W. Reviews of medical journal articles. Australian Journal of Herbal & Naturopathic Medicine 2019, 31, 110-116.	Wrong study design
223. Mendoza, N.; Perez, L.; Simoncini, T.; Genazzani, A. Inositol supplementation in women with polycystic ovary syndrome undergoing intracytoplasmic sperm injection: a systematic review and meta-analysis of randomized controlled trials. Reproductive biomedicine online 2017, 35, 529-535, doi:https://dx.doi.org/10.1016/j.rbmo.2017.07.005.	Wrong intervention
224. Meng, J.; Zhu, Y. Efficacy of simvastatin plus metformin for polycystic ovary syndrome: A meta-analysis of randomized controlled trials. European Journal of Obstetrics & Gynecology & Reproductive Biology 2020, 255, 19-24, doi:10.1016/j.ejogrb.2020.11.070.	Wrong comparator
225. Meng, J.; Zhu, Y. Efficacy of simvastatin plus metformin for polycystic ovary syndrome: a meta-analysis of randomized controlled trials. European journal of obstetrics and gynecology and reproductive biology 2021, 257, 19, doi:https://doi.org/10.1016/j.ejogrb.2020.11.070.	Wrong comparator
226. Merviel, P.; James, P.; Bouée, S.; Le Guillou, M.; Rince, C.; Nachtergaele, C.; Kerlan, V. Impact of myo-inositol treatment in women with polycystic ovary syndrome in assisted reproductive technologies. Reproductive health 2021, 18, 1-8, doi:10.1186/s12978-021-01073-3.	Wrong study design

Supplementary File 1

227. Miankouhi, T.A.; Azadi, M. Evaluation of medical and traditional treatments on the fertility of women with polycystic ovary syndrome. Journal of Reproduction and Infertility 2018, 19, 115-116.	Wrong study design
228. Miao, K.; Zhou, H. Effect of statins combined or not combined with metformin on polycystic ovary syndrome: A systematic review and meta-analysis. Journal of Obstetrics & Gynaecology Research 2022, 48, 1806-1815, doi:10.1111/jog.15301.	Wrong intervention
229. Mimoto, M.S.; Oyler, J.L.; Davis, A.M. Evaluation and Treatment of Hirsutism in Premenopausal Women. JAMA: Journal of the American Medical Association 2018, 319, 1613-1614, doi:10.1001/jama.2018.2611.	Wrong population
230. Mohammed, S.B.; Nayak, B.S. Polycystic ovarian syndrome trend in a nutshell. International Journal of Women's Health and Reproduction Sciences 2017, 5, 153-157, doi:http://dx.doi.org/10.15296/ijwhr.2017.28.	Wrong study design
231. Mohsin, R.; Saeed, A.; Baig, M.M.; Khan, M. Role of letrozole and metformin vs letrozole alone in ovulation induction in patients of polycystic ovarian syndrome. Pakistan Journal of Medical and Health Sciences 2019, 13, 350-352.	Wrong comparator
232. Mokaberinejad, R.; Rampisheh, Z.; Aliasl, J.; Akhtari, E. The comparison of fennel infusion plus dry cupping versus metformin in management of oligomenorrhoea in patients with polycystic ovary syndrome: a randomised clinical trial. Journal of Obstetrics & Gynaecology 2019, 39, 652-658, doi:10.1080/01443615.2018.1541232.	Wrong comparator
233. Molin, J.; Vanky, E.; Løvvik, T.S.; Dehlin, E.; Bixo, M. Gestational weight gain, appetite regulating hormones, and metformin treatment in polycystic ovary syndrome: A longitudinal, placebo-controlled study. BJOG: An International Journal of Obstetrics & Gynaecology 2022, 129, 1112-1121, doi:10.1111/1471-0528.17042.	Wrong population
234. Monastra, G.; Unfer, V.; Harrath, A.H.; Bizzarri, M. Combining treatment with myo-inositol and D-chiro-inositol (40:1) is effective in restoring ovary function and metabolic balance in PCOS patients. Gynecological endocrinology : the official journal of the International Society of Gynecological Endocrinology 2017, 33, 1-9, doi:https://dx.doi.org/10.1080/09513590.2016.1247797.	Wrong study design
235. Monastra, G.; Vucenik, I.; Harrath, A.H.; Alwasel, S.H.; Kamenov, Z.A.; Lagana, A.S.; Monti, N.; Fedeli, V.; Bizzarri, M. PCOS and Inositols: Controversial Results and Necessary Clarifications. Basic Differences Between D-Chiro and Myo-Inositol. Frontiers in endocrinology 2021, 12, 660381, doi:https://dx.doi.org/10.3389/fendo.2021.660381.	Wrong study design
236. Moramezi, F.; Ghanbarzadeh, R.; Nikbakht, R. VP07.11: Comparison of the efficacy of metformin and inofolic in ovulation induction in patients with resistant polycystic ovarian syndrome. Ultrasound in Obstetrics & Gynecology 2021, 58, 127-127, doi:10.1002/uog.24142.	Wrong publication type
237. Morgante, G.; Massaro, M.G.; Di Sabatino, A.; Cappelli, V.; De Leo, V. Therapeutic approach for metabolic disorders and infertility in women with PCOS. Gynecological endocrinology : the official journal of the International Society of Gynecological Endocrinology 2018, 34, 4-9, doi:https://dx.doi.org/10.1080/09513590.2017.1370644.	Wrong study design
238. Morotti, E.; Giovanni Artini, P.; Persico, N.; Battaglia, C. Metformin metabolic and vascular effects in overweight/moderately obese hyperinsulinemic PCOS patients treated with contraceptive vaginal ring: a pilot study. Gynecological endocrinology : the official journal of the International Society of Gynecological Endocrinology 2019, 35, 854-861, doi:https://dx.doi.org/10.1080/09513590.2019.1613361.	Wrong comparator
239. Morsy, A.A.; Sabri, N.A.; Mourad, A.M.; Mojahed, E.M.; Shawki, M.A. Randomized controlled open-label study of the effect of vitamin E supplementation on fertility in clomiphene citrate-resistant polycystic ovary syndrome. Journal of Obstetrics & Gynaecology Research 2020, 46, 2375-2382, doi:10.1111/jog.14467.	Wrong comparator
240. Mueck, A.O.; global, A.g. Treatment of hyperandrogenism in women with ethinylestradiol and cyproteroneacetate. The European journal of contraception & reproductive health care : the official journal of the European Society of Contraception 2017, 22, 170-171, doi:https://dx.doi.org/10.1080/13625187.2017.1328170.	Wrong study design
241. Muharam, R.; Srilestari, A.; Mihardja, H.; Callestya, L.J.; Harzif, A.K. Combination of electroacupuncture and pharmacological treatment improves insulin resistance in women with polycystic ovary syndrome: double-blind randomized clinical trial. International Journal of Reproductive BioMedicine 2022, 20, 289, doi:https://doi.org/10.18502/ijrm.v20i4.10900.	Wrong intervention
242. Muhas, C.; Nishad, K.M.; Ummunnoora, K.P.; Jushna, K.; Saheera, K.V.; Dilsha, K.P. Polycystic ovary syndrome (PCOS)-an overview. International Journal of Current Pharmaceutical Research 2018, 10, 5-9, doi:http://dx.doi.org/10.22159/ijcpr.2018v10i6.30969.	Wrong study design

Supplementary File 1

243. Naderpoor, N.; Gibson-Helm, M.; Shorakae, S.; Joham, A.; Bateson. Polycystic ovary syndrome Optimal management in general practice. Medicine Today 2017, 18, 55-59.	Wrong study design
244. Nas, K.; Tuu, L. A comparative study between myo-inositol and metformin in the treatment of insulin-resistant women. European review for medical and pharmacological sciences 2017, 21, 77-82.	Wrong study design
245. Nazirudeen, R.; Natarajan, V.; Jayaraman, S.; Subbiah, S. A randomized control trial comparing myoinositol based therapy in combination with metformin versus metformin monotherapy on the clinical and hormonal parameters in obese reproductive age women with polycystic ovarian syndrome. Indian Journal of Endocrinology and Metabolism 2022, 26, S16.	Wrong publication type
246. Nehra, J.; Kaushal, J.; Singhal, S.R.; Ghalaut, V. Effect of myoinositol versus metformin on biochemical profile in polycystic ovarian syndrome in women. British journal of clinical pharmacology 2019, 85, 1654, doi:https://doi.org/10.1111/bcp.13937.	Wrong comparator
247. Nehra, J.; Kaushal, J.; Singhal, S.R.; Ghalaut, V.S. A comparative study of myo inositol versus metformin on biochemical profile in polycystic ovarian syndrome in women. International Journal of Pharmaceutical Sciences and Research 2017, 8, 1664, doi:https://doi.org/10.13040/IJPSR.0975-8232.8(4).1664-70.	Wrong comparator
248. Nehra, J.; Kaushal, J.; Singhal, S.R.; Ghalaut, V.S. Comparision of myo-inositol versus metformin on anthropometric parameters in polycystic ovarian syndrome in women. International Journal of Pharmacy and Pharmaceutical Sciences 2017, 9, 144-148, doi:http://dx.doi.org/10.22159/ijpps.2017v9i4.16359.	Wrong comparator
249. Nemati, M.; Nemati, S.; Taheri, A.M.; Heidari, B. Comparison of metformin and N-acetyl cysteine, as an adjuvant to clomiphene citrate, in clomiphene-resistant women with polycystic ovary syndrome. Journal of gynecology obstetrics and human reproduction 2017, 46, 579-585, doi:https://dx.doi.org/10.1016/j.jogoh.2017.07.004.	Wrong comparator
250. Nikolakis, G.; Kyrgidis, A.; Zouboulis, C.C. Antiandrogens as a therapeutic option for hidradenitis suppurativa/ acne inversa. Experimental dermatology 2019, 28, 14, doi:https://doi.org/10.1111/exd.13893.	Wrong study design
251. Ning, D.; Rensong, Y.; Lizhen, W.; Hongjing, Y.; Ding, N.; Yue, R.; Wang, L.; Yang, H. Chinese herbal medicine on treating obese women with polycystic ovary syndrome: A systematic review and meta-analysis protocol. Medicine 2020, 99, 1-5, doi:10.1097/MD.0000000000022982.	Wrong intervention
252. Noreen, H.; Un Nisa Rab Nawaz, Z.; Khanum, W.; Syed, S.; Saleem, H.; Tanveer, I. Effectiveness of myoinositol versus metformin on biochemical profile of women with PCOS. BJOG 2021, 128, 236, doi:https://doi.org/10.1111/1471-0528.18-16715.	Wrong publication type
253. Notaro, A.L.G.; Neto, F.T.L. The use of metformin in women with polycystic ovary syndrome: an updated review. Journal of assisted reproduction and genetics 2022, 39, 573-579, doi:https://dx.doi.org/10.1007/s10815-022-02429-9.	Wrong study design
254. Nylander, M.; Frossing, S.; Clausen, H.V.; Kistorp, C.; Faber, J.; Skouby, S.O. Effects of liraglutide on ovarian dysfunction in polycystic ovary syndrome: a randomized clinical trial. Reproductive biomedicine online 2017, 35, 121, doi:https://doi.org/10.1016/j.rbmo.2017.03.023.	Wrong outcome
255. Oliveira, F.R.; Mamede, M.; Bizzi, M.F.; Rocha, A.L.L.; Ferreira, C.N.; Gomes, K.B.; Candido, A.L.; Reis, F.M. Effects of Short Term Metformin Treatment on Brown Adipose Tissue Activity and Plasma Irisin Levels in Women with Polycystic Ovary Syndrome: A Randomized Controlled Trial. Hormone and metabolic research = Hormon- und Stoffwechselforschung = Hormones et metabolisme 2020, 52, 718-723, doi:https://dx.doi.org/10.1055/a-1157-0615.	Wrong outcome
256. Ortiz-Flores, A.E.; Luque-Ramirez, M.; Escobar-Morreale, H.F. Pharmacotherapeutic management of comorbid polycystic ovary syndrome and diabetes. Expert opinion on pharmacotherapy 2018, 19, 1915-1926, doi:https://dx.doi.org/10.1080/14656566.2018.1528231.	Wrong study design
257. Otto-Buczkowska, E.; Grzyb, K.; Jainta, N. Polycystic ovary syndrome (PCOS) and the accompanying disorders of glucose homeostasis among girls at the time of puberty. Pediatric endocrinology, diabetes, and metabolism 2018, 24, 40-44, doi:https://dx.doi.org/10.18544/PEDM-24.01.0101.	Wrong study design
258. Ozay, A.C.; Emekci Ozay, O.; Okyay, R.E.; Gulekli, B. The effect of myoinositol on ovarian blood flows in women with polycystic ovary syndrome. Gynecological endocrinology : the official journal of the International Society of Gynecological Endocrinology 2019, 35, 237-241, doi:https://dx.doi.org/10.1080/09513590.2018.1520827.	Wrong study design

Supplementary File 1

259. Pal Singh Kochar, I.; Ramachandran, S.; Sethi, A. Metformin in Adolescent PCOS: The Way Forward. Pediatric endocrinology reviews : PER 2017, 15, 142-146, doi:https://dx.doi.org/10.17458/per.vol15.2017.prs.metforminadolescentpcos	Wrong study design
260. Pani, A.; Gironi, I.; Di Vieste, G.; Mion, E.; Bertuzzi, F.; Pintaudi, B. From Prediabetes to Type 2 Diabetes Mellitus in Women with Polycystic Ovary Syndrome: Lifestyle and Pharmacological Management. International Journal of Endocrinology 2020, 10.1155/2020/6276187, 1-10, doi:10.1155/2020/6276187.	Wrong study design
261. Papaetis, G.S.; Filippou, P.K.; Constantinidou, K.G.; Stylianou, C.S. Liraglutide: New Perspectives for the Treatment of Polycystic Ovary Syndrome. Clinical drug investigation 2020, 40, 695-713, doi:https://dx.doi.org/10.1007/s40261-020-00942-2.	Wrong study design
262. Pasquali, R. Contemporary approaches to the management of polycystic ovary syndrome. Therapeutic Advances in Endocrinology and Metabolism 2018, 9, 123-134, doi:http://dx.doi.org/10.1177/2042018818756790.	Wrong study design
263. Patel, S. Polycystic ovary syndrome (PCOS), an inflammatory, systemic, lifestyle endocrinopathy. The Journal of steroid biochemistry and molecular biology 2018, 182, 27-36, doi:https://dx.doi.org/10.1016/j.jsbmb.2018.04.008.	Wrong study design
264. Pedersen, A.J.T.; Stage, T.B.; Glintborg, D.; Andersen, M.; Christensen, M.M.H. The Pharmacogenetics of Metformin in Women with Polycystic Ovary Syndrome: a Randomized Trial (in press). Basic & clinical pharmacology & toxicology 2017.	Wrong study design
265. Pedersen, A.J.T.; Stage, T.B.; Glintborg, D.; Andersen, M.; Christensen, M.M.H. The Pharmacogenetics of Metformin in Women with Polycystic Ovary Syndrome: a Randomized Trial. Basic & clinical pharmacology & toxicology 2018, 122, 239, doi:https://doi.org/10.1111/bcpt.12874.	Wrong study design
266. Perichart-Perera, O.; Mier-Cabrera, J.; Flores-Robles, C.M.; Martinez-Cruz, N.; Arce-Sanchez, L.; Alvarado-Maldonado, I.N.; Montoya-Estrada, A.; Romo-Yanez, J.; Rodriguez-Cano, A.M.; Estrada-Gutierrez, G., et al. Intensive Medical Nutrition Therapy Alone or with Added Metformin to Prevent Gestational Diabetes Mellitus among High-Risk Mexican Women: A Randomized Clinical Trial. Nutrients 2021, 14, doi:https://dx.doi.org/10.3390/nu14010062.	Wrong population
267. Perichart-Perera, O.; Mier-Cabrera, J.; Flores-Robles, C.M.; Martinez-Cruz, N.; Arce-Sanchez, L.; Alvarado-Maldonado, I.N.; Montoya-Estrada, A.; Romo-Yanez, J.; Rodriguez-Cano, A.M.; Estrada-Gutierrez, G., et al. Intensive medical nutrition therapy alone or with added metformin to prevent gestational diabetes mellitus among high-risk mexican women: a randomized clinical trial. Nutrients 2022, 14, doi:https://doi.org/10.3390/nu14010062.	Wrong population
268. Pfieffer, M.L. Polycystic ovary syndrome: Diagnosis and management. Nurse Practitioner 2019, 44, 30-36, doi:10.1097/01.NPR.0000553398.50729.c0.	Wrong study design
269. Pkhaladze, L.; Russo, M.; Unfer, V.; Nordio, M.; Basciani, S.; Khomasuridze, A. Treatment of lean PCOS teenagers: a follow-up comparison between Myo-Inositol and oral contraceptives. European review for medical and pharmacological sciences 2021, 25, 7476-7485, doi:https://dx.doi.org/10.26355/eurrev_202112_27447.	Wrong comparator
270. Poojari, P.; Padgaonkar, A.; Paramanya, A.; Ali, A. Compendium of polycystic ovarian syndrome and its relevance in glycation and diabetes. Journal of Experimental and Clinical Medicine (Turkey) 2022, 39, 256-268, doi:https://dx.doi.org/10.52142/omujecm.39.1.49.	Wrong study design
271. Pourghasem, S.; Bazarganipour, F.; Taghavi, S.A.; Kutenaee, M.A. The effectiveness of inositol and metformin on infertile polycystic ovary syndrome women with resistant to letrozole. Archives of gynecology and obstetrics 2019, https://doi.org/10.1007/s00404-019-05064-5, doi:https://doi.org/10.1007/s00404-019-05064-5.	Wrong outcome
272. Powell, A. Choosing the Right Oral Contraceptive Pill for Teens. Pediatric clinics of North America 2017, 64, 343-358, doi:https://dx.doi.org/10.1016/j.pcl.2016.11.005.	Wrong study design
273. Practice Committee of the American Society for Reproductive Medicine. Electronic address, A.a.o.; Practice Committee of the American Society for Reproductive, M.; Penzias A, B.K.B.S.C.C.F.T.F.G.G.S.G.C.H.K.L.B.A.M.J.O.R.P. Role of metformin for ovulation induction in infertile patients with polycystic ovary syndrome (PCOS): a guideline. Fertility and sterility 2017, 108, 426-441, doi:https://dx.doi.org/10.1016/j.fertnstert.2017.06.026.	Wrong comparator
274. Pradas, I.; Rovira-Llopis, S.; Naudi, A.; Banuls, C.; Rocha, M.; Hernandez-Mijares, A.; Pamplona, R.; Victor, V.M.; Jove, M. Metformin induces lipid changes on sphingolipid species and oxidized lipids in polycystic ovary syndrome women. Scientific reports 2019, 9, 16033, doi:https://dx.doi.org/10.1038/s41598-019-52263-w.	Wrong study design
275. Pundir, J.; Psaroudakis, D.; Savnur, P.; Bhide, P.; Sabatini, L.; Teede, H.; Coomarasamy, A.; Khan, K.; Thangaratinam, S. Inositol treatment of anovulation in women with polycystic	Wrong intervention

Supplementary File 1

ovary syndrome: a meta-analysis of randomised trials. Human reproduction (Oxford, England) 2017, 32, i448.	
276. Pundir, J.; Psaroudakis, D.; Savnur, P.; Bhide, P.; Sabatini, L.; Teede, H.; Coomarasamy, A.; Thangaratinam, S. Inositol treatment of anovulation in women with polycystic ovary syndrome: a meta-analysis of randomised trials. BJOG: An International Journal of Obstetrics & Gynaecology 2018, 125, 299-308, doi:10.1111/1471-0528.14754.	Wrong intervention
277. Rajasekaran, K.; Malhotra, N. Randomised control trial comparing the effects of myoinositol to metformin on ART outcome in women with PCOS undergoing In-vitro fertilisation (IVF) cycle. Human reproduction. Conference: 36th annual meeting of the european human reproduction and embryology. ESHRE. Virtual meeting 2020, 35 Suppl 1, i396.	Wrong publication type
278. Rajasekaran, K.; Malhotra, N.; Mahey, R.; Khadgawat, R.; Kalaivani, M. Myoinositol versus metformin pretreatment in GnRH-antagonist cycle for women with PCOS undergoing IVF: a double-blinded randomized controlled study. Gynecological endocrinology : the official journal of the International Society of Gynecological Endocrinology 2022, 38, 140-147, doi:https://dx.doi.org/10.1080/09513590.2021.1981282.	Wrong comparator
279. Rani, N.; Kumar, P.; Mishra, A.; Sankuratri, B.; Sethi, S.; Gelada, K.; Tiwari, H. Efficacy of spironolactone in adult acne in polycystic ovary syndrome patients an original research. Journal of Pharmacy and Bioallied Sciences 2021, 13, S1659-S1663, doi:https://dx.doi.org/10.4103/jpbs.jpbs_391_21.	Wrong study design
280. Raperport, C.; Chronopoulou, E.; Homburg, R. Effects of metformin treatment on pregnancy outcomes in patients with polycystic ovary syndrome. Expert review of endocrinology & metabolism 2021, 16, 37-47, doi:https://dx.doi.org/10.1080/17446651.2021.1889366.	Wrong study design
281. Rapisarda, A.M.C.; Brescia, R.; Sapia, F.; Valenti, G.; Sarpietro, G.; Di Gregorio, L.M.; Gatta, A.N.D.; La Rosa, V.L.; Sergiampietri, C.; Corte, L.D., et al. Combined oral contraceptive in adolescent and young adult women: Current evidence and future perspectives. Current Women's Health Reviews 2019, 15, 109-118, doi:http://dx.doi.org/10.2174/1573404814666180914162053.	Wrong study design
282. Rashid, A.; Ganie, M.A.; Wani, I.A.; Bhat, G.A.; Shaheen, F.; Wani, I.A.; Shrivastava, M.; Shah, Z.A. Differential Impact of Insulin Sensitizers vs. Anti-Androgen on Serum Leptin Levels in Vitamin D Replete PCOS Women: A Six Month Open Labeled Randomized Study. Hormone and metabolic research = Hormon- und Stoffwechselforschung = Hormones et metabolisme 2020, 52, 89-94, doi:https://dx.doi.org/10.1055/a-1084-5441.	Full text not obtainable
283. Rashid, R.; Mir, S.A.; Kareem, O.; Ali, T.; Ara, R.; Malik, A.; Amin, F.; Bader, G.N. Polycystic ovarian syndrome-current pharmacotherapy and clinical implications. Taiwanese journal of obstetrics & gynecology 2022, 61, 40-50, doi:https://dx.doi.org/10.1016/j.tjog.2021.11.009.	Wrong study design
284. Rastegar, F.; Rezaee, Z.; Saedi, N.; Memari, R.; Tajpour, M. Comparison of Effect of Metformin Versus Combination of Folic Acid/Myo-inositol in Infertile Women with Poly Cystic Ovary Syndrome Undergoing in Vitro Fertilization: A Randomized Clinical Trial. Biomedical Research and Therapy 2021, 8, 4734, doi:https://doi.org/10.15419/bmrat.v8i12.710.	Wrong comparator
285. Rezk, M.; Shaheen, A.-E.; Saif El-Nasr, I. Clomiphene citrate combined with metformin versus letrozole for induction of ovulation in clomiphene-resistant polycystic ovary syndrome: a randomized clinical trial. Gynecological endocrinology : the official journal of the International Society of Gynecological Endocrinology 2018, 34, 298-300, doi:https://dx.doi.org/10.1080/09513590.2017.1395838.	Wrong intervention
286. Rodriguez-Gutierrez, R.; Montes-Villarreal, J.; Rodriguez-Velver, K.V.; Gonzalez-Velazquez, C.; Salcido-Montenegro, A.; Elizondo-Plazas, A.; Gonzalez-Gonzalez, J.G. Metformin Use and Vitamin B12 Deficiency: Untangling the Association. The American journal of the medical sciences 2017, 354, 165-171, doi:https://dx.doi.org/10.1016/j.amjms.2017.04.010.	Wrong study design
287. Rogowicz-Frontczak, A.; Majchrzak, A.; Zozulińska-Ziółkiewicz, D. Insulin resistance in endocrine disorders -- treatment options. Polish Journal of Endocrinology / Endokrynologia Polska 2017, 68, 334-350, doi:10.5603/EP.2017.0026.<br>Romanski, P.; Stanic, A.K. Practical Approach to the PCOS Patient. Current Obstetrics and Gynecology Reports 2017, 6, 11, doi:https://doi.org/10.1007/s13669-017-0190-6.	Wrong study design
288. Roy, S.B.; Roy, S.B. A Study of the Effect of Metformin Versus Myo-Inositol in the Management of PCOS; A Randomised Controlled Trial. Journal of the Indian Medical Association 2020, 118, 40.	Full text not obtainable
289. Ruan, X.; Gu, M.; Mueck, A.O. Pcos patients need anti-androgenic pretreatment before pregnancy. International journal of gynaecology and obstetrics 2018, 143, 809, doi:https://doi.org/10.1002/ijgo.12583.	Wrong publication type

290. Ruan, X.; Kubba, A.; Aguilar, A.; Mueck, A.O. Use of cyproterone acetate/ethinylestradiol in polycystic ovary syndrome: rationale and practical aspects. European journal of contraception & reproductive health care 2017, 22, 183-190, doi:10.1080/13625187.2017.1317735.	Wrong study design
291. Ruan, X.; Li, M.; Mueck, A.O. Why does Polycystic Ovary Syndrome (PCOS) Need Long-term Management? Current pharmaceutical design 2018, 24, 4685-4692, doi:https://dx.doi.org/10.2174/1381612825666190130104922.	Wrong study design
292. Ryssdal, M.; Vanky, E.; Stokkeland, L.M.T.; Jarmund, A.H.; Steinkjer, B.; Lovvik, T.S.; Madssen, T.S.; Iversen, A.C.; Giskeodegard, G.F. Y-012. Metformin changes serum cytokines in pregnant women with polycystic ovary syndrome. Y-012. Metformin changes serum cytokines in pregnant women with polycystic ovary syndrome 2021, 25, e21, doi:https://doi.org/10.1016/j.preghy.2021.07.017.	Wrong publication type
293. Sadeeqa, S.; Mustafa, T.; Latif, S. Polycystic ovarian syndrome-related depression in adolescent girls: A Review. Journal of Pharmacy and Bioallied Sciences 2018, 10, 55-59, doi:http://dx.doi.org/10.4103/JPBS.JPBS_1_18.	Wrong study design
294. Sadeghpoor, S.; Bolandghamat, B.; Sharajabad, F.A. The possibility and management sterateghies of pregnancy in women with polycystic ovary syndrome: A review article. Journal of Reproduction and Infertility 2017, 18, 231.	Wrong study design
295. Salehpour, S.; Nazari, L. New treatment in PCOS. International Journal of Reproductive BioMedicine 2017, 15, 1.	Wrong study design
296. Sam, S.; Ehrmann, D.A. Metformin therapy for the reproductive and metabolic consequences of polycystic ovary syndrome. Diabetologia 2017, 60, 1656-1661, doi:https://dx.doi.org/10.1007/s00125-017-4306-3.	Wrong study design
297. Sathyapalan, T.; Javed, Z.; Kilpatrick, E.S.; Coady, A.-M.; Atkin, S.L. Endocannabinoid receptor blockade increases vascular endothelial growth factor and inflammatory markers in obese women with polycystic ovary syndrome. Clinical endocrinology 2017, 86, 384-387, doi:https://dx.doi.org/10.1111/cen.13239.	Wrong comparator
298. Scheen, A.J.; Philips, J.C.; Kridelka, F. [Role of metformin in gynaecology and obstetrics]. Comment je traite ... Place de la metformine en gynecologie-obstetrique. 2018, 73, 597-602.	Wrong language
299. Scioscia, M.; Fascilla, F.; Bettocchi, S. Re: Inositol treatment of anovulation in women with polycystic ovary syndrome: a meta-analysis of randomised trials.	Wrong publication type
300. Pundir J, Psaroudakis D, Savnur P, et al. Inositol treatment of anovulation in women with polycystic ovary syndrome: a meta-analysis of randomised trials. BJOG 2018;125:299-308. Wiley-Blackwell: Malden, Massachusetts, 2018; Vol. 125, pp 385-385.	Wrong intervention
301. Scioscia, M.; Fascilla, F.; Bettocchi, S. Re: Inositol treatment of anovulation in women with polycystic ovary syndrome: a meta-analysis of randomised trials. BJOG : an international journal of obstetrics and gynaecology 2018, 125, 385, doi:https://dx.doi.org/10.1111/1471-0528.14810.	Wrong publication type
302. Seyam, E.; Hefzy, E. Long-term effects of combined simvastatin and metformin treatment on the clinical abnormalities and ovulation dysfunction in single young women with polycystic ovary syndrome. Gynecological endocrinology : the official journal of the International Society of Gynecological Endocrinology 2018, 34, 1073-1080, doi:https://dx.doi.org/10.1080/09513590.2018.1490405.	Wrong population
303. Shahnazi, M.; Farshbafkhalili, A.; Ghahremaninasab, P. Comparing the effects of combined low-dose oral contraceptives and vitex agnus on the improvement of symptoms polycystic ovarian syndrome: a triple-blind, randomized, controlled clinical trial. Journal of reproduction and infertility. Conference: 3rd international congress of the iranian society of embryology and reproductive biology, ISERB 2017, 18 Suppl 2, 209.	Full text not obtainable
304. Shahnazi, M.; Farshbafkhalili, A.; Ghahremaninasab, P. Comparing the effects of combined low-dose oral contraceptives and vitex agnus on the improvement of symptoms polycystic ovarian syndrome: A triple-blind, randomized, controlled clinical trial. Journal of Reproduction and Infertility 2017, 18, 209-210.	Wrong publication type
305. Shahriar, S.; Bahrami, S.; Sohran, F. Reviewing the effects of metformin on ovulation of women diagnosed with polycystic ovary syndrome (PCOS). Journal of Reproduction and Infertility 2018, 19, 119-120.	Wrong study design
306. Sharma, A.; Welt, C.K. Practical Approach to Hyperandrogenism in Women. The Medical clinics of North America 2021, 105, 1099-1116, doi:https://dx.doi.org/10.1016/j.mcna.2021.06.008.	Wrong study design
307. Sharma, S.; Mathur, D.K.; Paliwal, V.; Bhargava, P. Efficacy of Metformin in the Treatment of Acne in Women with Polycystic Ovarian Syndrome: A Newer Approach to Acne Therapy. Journal of Clinical & Aesthetic Dermatology 2019, 12, 34-38.	Wrong study design

308. Shen, W.; Jin, B.; Han, Y.; Wang, H.; Jiang, H.; Zhu, L.; Han, M.; Zhang, J.; Zhang, Y. The Effects of Salvia miltiorrhiza on Reproduction and Metabolism in Women with Polycystic Ovary Syndrome: A Systematic Review and Meta-Analysis. Evidence-based Complementary & Alternative Medicine (eCAM) 2021, 10.1155/2021/9971403, 1-12, doi:10.1155/2021/9971403.	Wrong intervention
309. Shokrpour, M.; Foroozanfard, F.; Afshar Ebrahimi, F.; Vahedpoor, Z.; Aghadavod, E.; Ghaderi, A.; Asemi, Z. Comparison of myo-inositol and metformin on glycemic control, lipid profiles, and gene expression related to insulin and lipid metabolism in women with polycystic ovary syndrome: a randomized controlled clinical trial. Gynecological Endocrinology 2019, 35, 406, doi:https://doi.org/10.1080/09513590.2018.1540570.	Wrong comparator
310. Showell, M.G.; Mackenzie-Proctor, R.; Jordan, V.; Hodgson, R.; Farquhar, C. Inositol for subfertile women with polycystic ovary syndrome. The Cochrane database of systematic reviews 2018, 12, CD012378, doi:https://dx.doi.org/10.1002/14651858.CD012378.pub2.	Wrong outcome
311. Shuai, W.; Tang, Z.; Gu, W.; Tong, X.; Cao, J. Impact of metformin on low-grade chronic inflammatory mediators in women with polycystic ovary syndrome: A meta-analysis. Latin American Journal of Pharmacy 2020, 39, 1388-1399.	Full text not obtainable
312. Siamashvili, M.; Davis, S.N. Update on the effects of GLP-1 receptor agonists for the treatment of polycystic ovary syndrome. Expert review of clinical pharmacology 2021, 14, 1081-1089, doi:https://dx.doi.org/10.1080/17512433.2021.1933433.	Wrong study design
313. Silva-Bermudez, L.S.; Toloza, F.J.K.; Perez-Matos, M.C.; de Souza, R.J.; Banfield, L.; Vargas-Villanueva, A.; Mendivil, C.O. Effects of oral contraceptives on metabolic parameters in adult premenopausal women: A meta-analysis. Endocrine Connections 2020, 9, 978-998, doi:http://dx.doi.org/10.1530/EC-20-0423.	Wrong comparator
314. Sohrevardi, S.M.; Heydari, B.; Azarpazhooh, M.R.; Teymourzadeh, M.; Simental-Mendia, L.E.; Atkin, S.L.; Sahebkar, A.; Karimi-Zarchi, M. Therapeutic Effect of Curcumin in Women with Polycystic Ovary Syndrome Receiving Metformin: A Randomized Controlled Trial. Advances in experimental medicine and biology 2021, 1308, 109-117, doi:https://dx.doi.org/10.1007/978-3-030-64872-5_9.	Wrong comparator
315. Soldat-Stankovic, V.; Pejicic, S.P.; Stankovic, S.; Jovanic, J.; Bjekic-Macut, J.; Livadas, S.; Ognjanovic, S.; Mastorakos, G.; Micic, D.; Macut, D. THE EFFECT OF MYOINOSITOL AND METFORMIN ON CARDIOVASCULAR RISK FACTORS IN WOMEN WITH POLYCYSTIC OVARY SYNDROME: a RANDOMIZED CONTROLLED TRIAL. Acta endocrinologica 2021, 17, 241, doi:https://doi.org/10.4183/aeb.2021.241.	Wrong comparator
316. Soldat-Stankovic, V.; Popovic-Pejicic, S.; Stankovic, S.; Prtina, A.; Malesevic, G.; Bjekic-Macut, J.; Livadas, S.; Ognjanovic, S.; Mastorakos, G.; Micic, D., et al. The effect of metformin and myoinositol on metabolic outcomes in women with polycystic ovary syndrome: role of body mass and adiponectin in a randomized controlled trial. Journal of endocrinological investigation 2022, 45, 583-595, doi:https://dx.doi.org/10.1007/s40618-021-01691-5.	Wrong comparator
317. Soliman, A.; De Sanctis, V.; Alaaraj, N.; Hamed, N. The clinical application of metformin in children and adolescents: A short update. Acta bio-medica : Atenei Parmensis 2020, 91, e2020086, doi:https://dx.doi.org/10.23750/abm.v91i3.10127.	Wrong study design
318. Song, S.Y.; Yang, J.B.; Song, M.S.; Oh, H.Y.; Lee, G.W.; Lee, M.; Ko, Y.B.; Lee, K.H.; Chang, H.K.; Kwak, S.M., et al. Effect of pretreatment with combined oral contraceptives on outcomes of assisted reproductive technology for women with polycystic ovary syndrome: a meta-analysis. Archives of Gynecology & Obstetrics 2019, 300, 737-750, doi:10.1007/s00404-019-05210-z.	Wrong outcome
319. Song, Y.; Wang, H.; Huang, H.; Zhu, Z. Comparison of the efficacy between NAC and metformin in treating PCOS patients: a meta-analysis. Gynecological endocrinology : the official journal of the International Society of Gynecological Endocrinology 2020, 36, 204-210, doi:https://dx.doi.org/10.1080/09513590.2019.1689553.	Wrong comparator
320. Song, Y.; Wang, H.; Zhu, Z.; Huang, H. Effects of Metformin and Exercise in Polycystic Ovary Syndrome: Systematic Review and Meta-Analysis. Hormone and metabolic research = Hormon- und Stoffwechselforschung = Hormones et metabolisme 2021, 53, 738-745, doi:https://dx.doi.org/10.1055/a-1666-8979.	Full text not obtainable
321. Sova, H.; Unkila-Kallio, L.; Tiitinen, A.; Hippelainen, M.; Perheentupa, A.; Tinkanen, H.; Puukka, K.; Bloigu, R.; Piltonen, T.; Tapanainen, J., et al. Decrease in serum AMH levels during prepregnancy metformin therapy associates with improved pregnancy and live-birth rates in women with PCOS: a multicentre, double-blind, placebo-controlled RCT. Human reproduction (Oxford, England) 2019, 34, i145.	Wrong publication type
322. Sova, H.; Unkila-Kallio, L.; Tiitinen, A.; Hippelainen, M.; Perheentupa, A.; Tinkanen, H.; Puukka, K.; Bloigu, R.; Piltonen, T.; Tapanainen, J., et al. Decrease in serum AMH levels	Wrong publication type

Supplementary File 1

	during prepregnancy metformin therapy associates with improved pregnancy and live-birth rates in women with PCOS: a multicentre, double-blind, placebo-controlled RCT. Human reproduction. Conference: 35th annual meeting of the european society of human reproduction and embryology. ESHRE. Vienna, austria 2019, 34 Suppl 1.	
323.	Stefanaki, C.; Bacopoulou, F.; Kandaraki, E.; Boschiero, D.; Diamandi-Kandarakis, E. Lean Women on Metformin and Oral Contraceptives for Polycystic Ovary Syndrome Demonstrate a Dehydrated Osteosarcopenic Phenotype: A Pilot Study. Nutrients 2019, 11, 2055, doi:10.3390/nu11092055.	Wrong study design
324.	Stener-Victorin, E.; Zhang, H.; Li, R.; Friden, C.; Li, D.; Wang, W.; Wang, H.; Chang, C.; Li, S.; Huo, Z., et al. Acupuncture or metformin to improve insulin resistance in women with polycystic ovary syndrome: study protocol of a combined multinational cross sectional case-control study and a randomised controlled trial. BMJ open 2019, 9, e024733, doi:https://dx.doi.org/10.1136/bmjopen-2018-024733.	Wrong study design
325.	Stewart, C.E.; Sohrabji, F.; Agarwal, A.A.A.A.A.A.B.B.B.B.B.B.B.-B.B.B.B.B.B.B.C.C.C.C.C.C.C.D.D.D. Gonadal hormones and stroke risk: PCOS as a case study. Frontiers in Neuroendocrinology 2020, 58, doi:https://dx.doi.org/10.1016/j.yfrne.2020.100853.	Wrong study design
326.	Street, M.E.; Cirillo, F.; Catellani, C.; Dauriz, M.; Lazzeroni, P.; Sartori, C.; Moghetti, P. Current treatment for polycystic ovary syndrome: focus on adolescence. Minerva pediatrica 2020, 72, 288-311, doi:https://dx.doi.org/10.23736/S0026-4946.20.05861-2.	Wrong study design
327.	Stridsklev, S.; Salvesen, O.; Salvesen, K.A.; Carlsen, S.M.; Husoy, M.A.; Vanky, E. Uterine artery doppler in pregnancy: women with PCOS compared to healthy controls. Endocrine reviews 2017, 38.	Wrong publication type
328.	Sung, C.T.; Chao, T.; Lee, A.; Foulad, D.P.; Choi, F.; Juhasz, M.; Dobry, A.; Mesinkovska, N.A. Oral Metformin for Treating Dermatological Diseases: A Systematic Review. Journal of drugs in dermatology : JDD 2020, 19, 713-720, doi:https://dx.doi.org/10.36849/JDD.2020.4874.	Wrong comparator
329.	Syed, S.Z.; Akram, F.; Aftab Hassan, S.M. Comparison of efficacy of metformin versus Pioglitazone on ovulation in patients of polycystic ovarian syndrome. Pakistan Journal of Medical and Health Sciences 2018, 12, 1240-1242.	Wrong outcome
330.	Tagliaferri, V.; Romualdi, D.; Immediata, V.; De Cicco, S.; Di Florio, C.; Lanzone, A.; Guido, M. Metformin vs myoinositol: which is better in obese polycystic ovary syndrome patients? A randomized controlled crossover study. Clinical endocrinology 2017, 86, 725-730, doi:https://dx.doi.org/10.1111/cen.13304.	Wrong comparator
331.	Talaat, B.; Ammar, I.M.M. The added value of cinnamon to metformin in controlling symptoms of polycystic ovary syndrome, a randomized controlled trial. Middle East Fertility Society Journal 2018, 23, 440-445, doi:http://dx.doi.org/10.1016/j.mefs.2018.03.005.	Wrong comparator
332.	Tan, J.; Zhou, G.J.; Wang, Q.Y.; Liu, T.T.; Cao, Q.; Huang, W. [Effect of metformin and rosiglitazone in non-obese polycystic ovary syndrome women with insulin resistance]. Zhonghua fu chan ke za zhi 2021, 56, 467-473, doi:https://dx.doi.org/10.3760/cma.j.cn112141-20210424-00224.	Wrong language
333.	Tang, L.; Ye, J.; Shi, Y.; Zhu, X. Association between CD16++ monocytes in peripheral blood and clinical features and short-term therapeutic effects of polycystic ovary syndrome. International Journal of Gynecology & Obstetrics 2019, 145, 12-17, doi:10.1002/ijgo.12779.	Wrong study design
334.	Tao, T.; Wu, P.; Wang, Y.; Liu, W. Comparison of glycemic control and β-cell function in new onset T2DM patients with PCOS of metformin and saxagliptin monotherapy or combination treatment. BMC endocrine disorders 2018, 18, 1-1, doi:10.1186/s12902-018-0243-5.	Wrong population
335.	Tauqir, S.; Israr, M.; Rauf, B.; Malik, M.O.; Habib, S.H.; Shah, F.A.; Usman, M.; Raza, M.A.; Shah, I.; Badshah, H., et al. Acetyl-L-Carnitine Ameliorates Metabolic and Endocrine Alterations in Women with PCOS: A Double-Blind Randomized Clinical Trial. Advances in therapy 2021, 38, 3842-3856, doi:https://dx.doi.org/10.1007/s12325-021-01789-5.	Wrong comparator
336.	Tay, C.T.; Joham, A.E.; Hiam, D.S.; Gadalla, M.A.; Pundir, J.; Thangaratinam, S.; Teede, H.J.; Moran, L.J. Pharmacological and surgical treatment of nonreproductive outcomes in polycystic ovary syndrome: An overview of systematic reviews. Clinical endocrinology 2018, 89, 535-553, doi:https://dx.doi.org/10.1111/cen.13753.	Wrong study design
337.	Tehrani, F.R.; Amiri, M. Polycystic ovary syndrome in adolescents: Challenges in diagnosis and treatment. International Journal of Endocrinology and Metabolism 2019, 17, e91554, doi:http://dx.doi.org/10.5812/ijem.91554.	Wrong study design
338.	Tennilä, J.; Jääskeläinen, J.; Utriainen, P.; Voutilainen, R.; Häkkinen, M.; Auriola, S.; Morin-Papunen, L.; Liimatta, J. PCOS Features and Steroid Profiles Among Young Adult Women	Wrong study design

Supplementary File 1

with a History of Premature Adrenarche. Journal of Clinical Endocrinology & Metabolism 2021, 106, e3335-e3345, doi:10.1210/clinem/dgab385.	
339. Toosy, S.; Sodi, R.; Pappachan, J.M. Lean polycystic ovary syndrome (PCOS): an evidence-based practical approach. Journal of Diabetes and Metabolic Disorders 2018, 17, 277-285, doi:http://dx.doi.org/10.1007/s40200-018-0371-5.	Wrong study design
340. Trouva, A.; Alvarsson, M.; Calissendorff, J.; Asvold, B.O.; Vanky, E.; Hirschberg, A.L. Thyroid Status During Pregnancy in Women With Polycystic Ovary Syndrome and the Effect of Metformin. Frontiers in endocrinology 2022, 13, 772801, doi:https://dx.doi.org/10.3389/fendo.2022.772801.	Wrong outcome
341. Tso, L.O.; Costello, M.F.; Albuquerque, L.E.T.; Andriolo, R.B.; Macedo, C.R. Metformin treatment before and during IVF or ICSI in women with polycystic ovary syndrome. The Cochrane database of systematic reviews 2020, 12, CD006105, doi:https://dx.doi.org/10.1002/14651858.CD006105.pub4.	Wrong population
342. Tzotzas, T.; Karras, S.N.; Katsiki, N. Glucagon-Like Peptide-1 (GLP-1) Receptor Agonists in the Treatment of Obese Women with Polycystic Ovary Syndrome. Current vascular pharmacology 2017, 15, 218-229, doi:https://dx.doi.org/10.2174/1570161114666161221115324.	Wrong study design
343. Udesen, P.B.; Glintborg, D.; Sorensen, A.E.; Svendsen, R.; Nielsen, N.L.S.; Wissing, M.L.M.; Andersen, M.S.; Englund, A.L.M.; Dalgaard, L.T. Metformin decreases mir-122, mir-223 and mir-29a in women with polycystic ovary syndrome. Endocrine Connections 2020, 9, 1075, doi:https://doi.org/10.1530/EC-20-0195.	Wrong outcome
344. Underdal, M.O.; Salvesen, Ø.; Henriksen, A.H.; Andersen, M.; Vanky, E. Impaired Respiratory Function in Women With PCOS Compared With Matched Controls From a Population-Based Study. Journal of Clinical Endocrinology & Metabolism 2019, 10.1210/clinem/dgz053, N.PAG-N.PAG, doi:10.1210/clinem/dgz053.	Wrong study design
345. Underdal, M.O.; Stridsklev, S.; Andresen, M.S.; Vanky, E. Metabolic health in women with PCOS-5-11 years' followup after metformin or placebo in pregnancy. Endocrine reviews 2017, 38.	Full text not obtainable
346. Underdal, M.O.; Stridsklev, S.; Oppen, I.H.; Høgetveit, K.; Andersen, M.S.; Vanky, E. Does Metformin Treatment During Pregnancy Modify the Future Metabolic Profile in Women With PCOS? Journal of Clinical Endocrinology & Metabolism 2018, 10.1210/jc.2018-00485, N.PAG-N.PAG, doi:10.1210/jc.2018-00485.	Wrong population
347. Vatopoulou, A.; Tziomalos, K. Management of obesity in adolescents with polycystic ovary syndrome. Expert opinion on pharmacotherapy 2020, 21, 207-211, doi:https://dx.doi.org/10.1080/14656566.2019.1701655.	Wrong study design
348. Vedtofte, L.; Foghsgaard, S.; Zierau, L.; VilsbØLl, T.; Knop, F.K. 1186-P: Lean Women with Polycystic Ovary Syndrome and Insulin Resistance Have Normal Incretin Effect, which Is Unaffected by Metformin Therapy. Diabetes 2019, 68, N.PAG-N.PAG, doi:10.2337/db19-1186-P.	Wrong publication type
349. Venter, A. Obesity, Oligomenorrhoea and PCOS in Adolescence. Obstetrics and Gynaecology Forum 2018, 28, 27-30.	Wrong study design
350. Vine, D.; Proctor, E.; Weaver, O.; Ghosh, M.; Maximova, K.; Proctor, S. A Pilot Trial: fish Oil and Metformin Effects on ApoB-Remnants and Triglycerides in Women with Polycystic Ovary Syndrome. Journal of the Endocrine Society 2021, 5, doi:https://doi.org/10.1210/jendso/bvab114.	Wrong comparator
351. Walker, K.; Decherney, A.H.; Saunders, R. Menstrual Dysfunction in PCOS. Clinical Obstetrics & Gynecology 2021, 64, 119-125, doi:10.1097/GRF.0000000000000596.	Wrong study design
352. Wang, J.; Zhu, L.; Hu, K.; Tang, Y.; Zeng, X.; Liu, J.; Xu, J. Effects of metformin treatment on serum levels of C-reactive protein and interleukin-6 in women with polycystic ovary syndrome: a meta-analysis: A PRISMA-compliant article. Medicine 2017, 96, e8183-e8183, doi:10.1097/MD.0000000000008183.	Wrong comparator
353. Wang, L.; Liang, R.; Tang, Q.; Zhu, L. An Overview of Systematic Reviews of Using Chinese Medicine to Treat Polycystic Ovary Syndrome. Evidence-based Complementary and Alternative Medicine 2021, 2021, 9935536, doi:http://dx.doi.org/10.1155/2021/9935536	Wrong intervention
354. Wang, R.; Kim, B.V.; van Wely, M.; Johnson, N.P.; Costello, M.F.; Zhang, H.; Ng, E.H.Y.; Legro, R.S.; Bhattacharya, S.; Norman, R.J., et al. Treatment strategies for women with WHO group II anovulation: systematic review and network meta-analysis. BMJ (Clinical research ed.) 2017, 356, j138, doi:https://dx.doi.org/10.1136/bmj.j138.	Wrong population
355. Wang, R.; Li, W.; Bordewijk, E.M.; Legro, R.S.; Zhang, H.; Wu, X.; Gao, J.; Morin-Papunen, L.; Homburg, R.; Konig, T.E., et al. First-line ovulation induction for polycystic ovary	Wrong outcome

	syndrome: an individual participant data meta-analysis. Human reproduction update 2019, 25, 717-732, doi:https://dx.doi.org/10.1093/humupd/dmz029.	
356.	Wang, Y.-W.; He, S.-J.; Feng, X.; Cheng, J.; Luo, Y.-T.; Tian, L.; Huang, Q. Metformin: a review of its potential indications. Drug design, development and therapy 2017, 11, 2421-2429, doi:https://dx.doi.org/10.2147/DDDT.S141675.	Wrong study design
357.	Wawrzkiewicz-Jalowiecka, A.; Kowalczyk, K.; Trybek, P.; Jarosz, T.; Radosz, P.; Setlak, M.; Madej, P. In Search of New Therapeutics-Molecular Aspects of the PCOS Pathophysiology: Genetics, Hormones, Metabolism and Beyond. International journal of molecular sciences 2020, 21, doi:https://dx.doi.org/10.3390/ijms21197054.	Wrong study design
358.	Wen, Y.; Ma, H.L.; Wu, X.K. Acupuncture and clomiphene interventions in PCOS conversely affect the insulin resistance profiles in early pregnancy subjects: a secondary analysis of a randomized controlled trial. Journal of obstetrics and gynaecology research 2017, 43, 160, doi:https://doi.org/10.1111/jog.13394.	Wrong publication type
359.	Wenjing, L.; Hongbo, H.; Guofang, Z.; Zhanzhong, M.; Jing, L.; Fanxiang, L.; Li, W.; Hu, H.; Zou, G.; Ma, Z., et al. Therapeutic effects of puerarin on polycystic ovary syndrome: A randomized trial in Chinese women. Medicine 2021, 100, 1-8, doi:10.1097/MD.0000000000026049.	Wrong comparator
360.	Witchel, S.F.; Oberfield, S.E.; Peña, A.S. Polycystic Ovary Syndrome: Pathophysiology, Presentation, and Treatment With Emphasis on Adolescent Girls. Journal of the Endocrine Society 2019, 3, 1545-1573, doi:10.1210/js.2019-00078.	Wrong study design
361.	Wiweko, B.; Susanto, C. The Effect of Metformin and Cinnamon on Serum Anti-Mullerian Hormone in Women Having PCOS: a Double-Blind, Randomized, Controlled Trial. Journal of Human Reproductive Sciences 2017, 10, 31, doi:https://doi.org/10.4103/jhrs.JHRS9016.	Wrong comparator
362.	Wojciechowska, A.; Osowski, A.; Jozwik, M.; Gorecki, R.; Rynkiewicz, A.; Wojtkiewicz, J. Inositols' Importance in the Improvement of the Endocrine-Metabolic Profile in PCOS. International journal of molecular sciences 2019, 20, doi:https://dx.doi.org/10.3390/ijms20225787.	Wrong study design
363.	Woodward, A.; Broom, D.; Harrop, D.; Lahart, I.; Carter, A.; Dalton, C.; Metwally, M.; Klonizakis, M. The effects of physical exercise on cardiometabolic outcomes in women with polycystic ovary syndrome not taking the oral contraceptive pill: a systematic review and meta-analysis. Journal of Diabetes and Metabolic Disorders 2019, 18, 597-612, doi:http://dx.doi.org/10.1007/s40200-019-00425-y.	Wrong intervention
364.	Wu, Y.; Tu, M.; Huang, Y.; Liu, Y.; Zhang, D. Association of Metformin With Pregnancy Outcomes in Women With Polycystic Ovarian Syndrome Undergoing In Vitro Fertilization: A Systematic Review and Meta-analysis. JAMA network open 2020, 3, e2011995-e2011995, doi:10.1001/jamanetworkopen.2020.11995.	Wrong outcome
365.	Xie, L.; Zhang, D.; Ma, H.; He, H.; Xia, Q.; Shen, W.; Chang, H.; Deng, Y.; Wu, Q.; Cong, J., et al. The Effect of Berberine on Reproduction and Metabolism in Women with Polycystic Ovary Syndrome: A Systematic Review and Meta-Analysis of Randomized Control Trials. Evidence-based Complementary & Alternative Medicine (eCAM) 2019, 10.1155/2019/7918631, 1-15, doi:10.1155/2019/7918631.	Wrong intervention
366.	Xu, J.; Zuo, Y. [Efficacy of acupuncture as adjunctive treatment on infertility patients with polycystic ovary syndrome]. Zhongguo zhen jiu = Chinese acupuncture & moxibustion 2018, 38, 358-361, doi:https://dx.doi.org/10.13703/j.0255-2930.2018.04.004.	Wrong language
367.	Xu, Q.; Xie, Q. Long-term effects of prenatal exposure to metformin on the health of children based on follow-up studies of randomized controlled trials: a systematic review and meta-analysis. Springer Nature: , <Blank>, 2019; Vol. 299, pp 1295-1303.	Wrong outcome
368.	Xu, Z.; Meng, L.; Pan, C.; Chen, X.; Huang, X.; Yang, H. Does oral contraceptives pretreatment affect the pregnancy outcome in polycystic ovary syndrome women undergoing ART with GnRH agonist protocol? Gynecological endocrinology : the official journal of the International Society of Gynecological Endocrinology 2019, 35, 124-127, doi:https://dx.doi.org/10.1080/09513590.2018.1500535.	Wrong study design
369.	Yanbo, L.; Yupei, S.; Jiping, X.; Linlin, C.; Guang, Z.; Liu, Y.; Shao, Y.; Xie, J.; Chen, L.; Zhu, G. The efficacy and safety of metformin combined with simvastatin in the treatment of polycystic ovary syndrome: A meta-analysis and systematic review. Medicine 2021, 100, 1-8, doi:10.1097/MD.0000000000026622.	Wrong intervention
370.	Yang, D.; Zhao, M.; Tan, J. [Effect of polycystic ovary syndrome treated with the periodic therapy of acupuncture]. Zhongguo zhen jiu = Chinese acupuncture & moxibustion 2017, 37, 825-829, doi:https://dx.doi.org/10.13703/j.0255-2930.2017.08.007.	Wrong language
371.	Yang, J.; Liu, Y.; Huang, J.; Xu, J.; You, X.; Lin, Q.; Zhang, J.; Dun, J.; Huang, S. [Acupuncture and Chinese medicine of artificial cycle therapy for insulin resistance of	Wrong language

Supplementary File 1

	polycystic ovary syndrome with phlegm damp type and its mechanism]. Zhongguo zhen jiu = Chinese acupuncture & moxibustion 2017, 37, 1163-1168, doi:https://dx.doi.org/10.13703/j.0255-2930.2017.11.007.	
372.	Yao, K.; Bian, C.; Zhao, X. Association of polycystic ovary syndrome with metabolic syndrome and gestational diabetes: Aggravated complication of pregnancy (Review). Experimental and Therapeutic Medicine 2017, 14, 1271-1276, doi:http://dx.doi.org/10.3892/etm.2017.4642.	Wrong study design
373.	Yen, H.; Chang, Y.-T.; Yee, F.-J.; Huang, Y.-C. Metformin Therapy for Acne in Patients with Polycystic Ovary Syndrome: A Systematic Review and Meta-analysis. American journal of clinical dermatology 2021, 22, 11-23, doi:10.1007/s40257-020-00565-5.	Wrong outcome
374.	Young, C.C.; Monge, M. Polycystic Ovary Syndrome in Primary Care: It Takes a Village. Journal for Nurse Practitioners 2019, 15, 694-695, doi:10.1016/j.nurpra.2019.05.008.	Wrong study design
375.	Yousuf, S.D.; Ganie, M.A.; Jeelani, S.; Mudassar, S.; Shah, Z.A.; Zargar, M.A.; Amin, S.; Wani, I.A.; Rashid, F. Effect of six-month use of oral contraceptive pills on plasminogen activator inhibitor-1 & factor VIII among women with polycystic ovary syndrome: An observational pilot study. The Indian journal of medical research 2018, 148, S151-S155, doi:https://dx.doi.org/10.4103/ijmr.IJMR_1899_17.	Wrong study design
376.	Zeng, L.; Yang, K. Effectiveness of myoinositol for polycystic ovary syndrome: a systematic review and meta-analysis. Endocrine 2018, 59, 30-38, doi:https://dx.doi.org/10.1007/s12020-017-1442-y.	Wrong intervention
377.	Zhang, J.; Si, Q.; Li, J. Therapeutic effects of metformin and clomiphene in combination with lifestyle intervention on infertility in women with obese polycystic ovary syndrome. Pakistan journal of medical sciences 2017, 33, 8, doi:https://doi.org/10.12669/pjms.331.11764.	Wrong intervention
378.	Zhang, J.; Su, M.; Xu, L.; Yang, Z.; Yin, W.; Nie, Y.; Qiao, X.; Cheng, R.; Ma, Y. [Efficacy and metabolic safety of long-term treatment with ethinyl oestradiol/cyproterone and desogestrel/ethinyl oestradiol tablets in women with polycystic ovary syndrome]. Nan fang yi ke da xue xue bao = Journal of Southern Medical University 2018, 38, 917-922, doi:https://dx.doi.org/10.3969/j.issn.1673-4254.2018.08.03.	Wrong language
379.	Zhang, S.-W.; Zhou, J.; Gober, H.-J.; Leung, W.T.; Wang, L. Effect and mechanism of berberine against polycystic ovary syndrome. Biomedicine & pharmacotherapy = Biomedecine & pharmacotherapie 2021, 138, 111468, doi:https://dx.doi.org/10.1016/j.biopha.2021.111468.	Wrong study design
380.	Zhang, Y.; Guo, X.; Ma, S.; Ma, H.; Li, H.; Wang, Y.; Qin, Z.; Wu, X.; Han, Y.; Han, Y. The Treatment with Complementary and Alternative Traditional Chinese Medicine for Menstrual Disorders with Polycystic Ovary Syndrome. Evidence-based Complementary & Alternative Medicine (eCAM) 2021, 10.1155/2021/6678398, 1-19, doi:10.1155/2021/6678398.	Wrong study design
381.	Zhao, J.; Liu, X.; Zhang, W. The Effect of Metformin Therapy for Preventing Gestational Diabetes Mellitus in Women with Polycystic Ovary Syndrome: A Meta-Analysis. Experimental and clinical endocrinology & diabetes : official journal, German Society of Endocrinology [and] German Diabetes Association 2020, 128, 199-205, doi:https://dx.doi.org/10.1055/a-0603-3394.	Full text not obtainable
382.	Zhao, Y.X.; Wang, L.J.; Gong, F.Y.; Pan, H.; Miao, H.; Duan, L.; Yang, H.B.; Zhu, H.J. [Effects of orlistat and metformin on metabolism and gonadal function in overweight or obese patients with polycystic ovary syndrome]. Zhonghua nei ke za zhi 2021, 60, 1165-1168, doi:https://dx.doi.org/10.3760/cma.j.cn112138-20210302-00171.	Wrong language
383.	Zhou, K.; Zhang, J.; Xu, L.; Lim, C.E.D. Chinese herbal medicine for subfertile women with polycystic ovarian syndrome. The Cochrane database of systematic reviews 2021, 6, CD007535, doi:https://dx.doi.org/10.1002/14651858.CD007535.pub4.	Wrong intervention
384.	Zimmerman, L.D.; Setton, R.; Pereira, N.; Rosenwaks, Z. Contemporary Management of Polycystic Ovarian Syndrome. Clinical obstetrics and gynecology 2019, 62, 271-281, doi:https://dx.doi.org/10.1097/GRF.0000000000000449.	Wrong study design

From updated search:

	Citation	Reason
1.	Zhang, M. 2023. Effects of Orlistat combined with an anti-androgenic oral contraceptive pill on cardiovascular risk factors in overweight or obese PCOS patients.	Full text not available
2.	Unknown 2022. A Clinical Trial to Evaluate the Efficacy, Tolerability, and Safety of a Fixed Dose Combination of Spironolactone, Pioglitazone & Metformin (SPIOMET) in Polycystic Ovary Syndrome (PCOS)	Wrong study design

Supplementary File 1

3.	Chen, Z. 2022. A Randomized Cohort Study: Is It Worth the Time to Receive Antiandrogenic Pretreatment Before Ovulation Induction for Women With Polycystic Ovary Syndrome?.	Wrong intervention
4.	Hughes, L. 2022. THE EFFECT OF CLOMIPHENE, METFORMIN, OR BOTH ON ANTI-MÜLLERIAN HORMONE (AMH) LEVELS: a NEW LOOK AT THE PREGNANCY IN POLYCYSTIC OVARIAN SYNDROME TRIAL (PPCOS I)	Wrong study design
5.	Bassols, J. 2023. Effects of half-dose spiomet treatment in girls with early puberty and accelerated bone maturation: a multicenter, randomized, placebo-controlled study protocol.	Wrong study design
6.	Sabbadin, C. 2023. Long-Lasting Effects of Spironolactone after its Withdrawal in Patients with Hyperandrogenic Skin Disorders.	Wrong study design
7.	Winnett, C. 2022. LOW-DOSE FLUTAMIDE ALTERS ABDOMINAL ADIPOGENIC FUNCTION IN NORMAL-WEIGHT POLYCYSTIC OVARY SYNDROME WOMEN (PCOS); A DOUBLE-BLIND, PLACEBO-CONTROLLED, RANDOMIZED CLINICAL TRIAL.	Wrong study design
8.	Long, T. 2022. Effects of Low-Dose Spironolactone Combined with Metformin or Either Drug Alone on Insulin Resistance in Patients with Polycystic Ovary Syndrome: A Pilot Study.	Wrong intervention

Supplementary File 2

Supplementary Table S1: Risk of bias assessments of included studies

Study ID			Selection bias		Performance bias			Detection bias			Attrition bias		Reporting bias	Confounding		Other bias			Overall RoB score
	Clearly focused PICO?	Specified inclusion/ exclusion criteria?	Random sequence generation?	Centralised/ concealed allocation	Participants blinded?	Investigators blinded?	Groups treated the same?	Outcome assessors blinded?	All outcomes measured in standard, valid way?	Outcomes assessed objectively and independently?	Drop-outs reported?	ITT analysis	Free of selective reporting?	Groups similar at baseline?	Controlled for confounding if present	Funding/ COI likely to influence researchers?	Sufficiently powered?	Adequate statistical analysis?	
Alpañés 2017	Yes	Yes	Yes	No	No	No	Partial	NR	Partial	Partial	Yes	Yes	Yes	Partial	No	No	Yes	Partial	High
Amiri 2014	Yes	Yes	Yes	Yes	Yes	No	NR	No	Yes	NR	Yes	Yes	NR	Yes	N/A	No	Partial	Yes	High*
Diri 2017	Yes	Yes	No	No	Partial	Partial	Partial	Yes	Partial	No	NR	No	NR	Partial	No	Partial	NR	Yes	High
Dumesic 2023	Yes	Yes	Yes	Yes	Yes	Yes	Yes	Yes	Yes	Yes	Yes	No	Yes	Partial	No	No	Partial	Yes	Moderate
Falsetti 1997<br>Falsetti 1999	Yes	Yes	NR	NR	No	NR	Partial	NR	Yes	Yes	Yes	No	NR	Yes	Partial	NR	Partial	Partial	High
Gambineri 2004<br>Gambineri 2006	Yes	Yes	Yes	Yes	Yes	NR	Yes	Yes	Yes	Yes	Yes	No	NR	Yes	Yes	No	Yes	Yes	Low
Ganie 2004	Yes	Yes	Yes	Yes	NR	NR	Yes	NR	Yes	Yes	Yes	Yes	NR	Yes	N/A	NR	NR	Yes	Moderate
Ganie 2013	Yes	Yes	Yes	Yes	NR	NR	Yes	NR	Yes	NR	Yes	Yes	NR	Yes	N/A	NR	NR	Yes	Low
Hagag 2014	Yes	Yes	Yes	NR	NR	NR	Yes	NR	Yes	Yes	Yes	No	NR	Yes	Yes	No	Yes	Yes	Moderate
Ibanez 2004	Yes	Yes	Yes	NR	No	No	Yes	NR	Yes	NR	Yes	Yes	NR	NR	NR	NR	NR	Yes	Moderate
Ibanez 2017<br>Diaz 2018<br>Malpique 2019<br>Ibanez 2020<br>De Zegher 2021	Yes	Yes	Yes	Yes	NR	Yes	Yes	No	Yes	Yes	Yes	NR	Yes	Yes	N/A	No	NR	Yes	Moderate
Mazza 2014	Yes	Yes	Yes	NR	NR	NR	Yes	NR	Yes	NR	Yes	Yes	NR	Partial	N/A	No	NR	Yes	Low
Mehrabian 2016	Yes	Yes	Yes	Yes	No	Yes	Yes	NR	Yes	Yes	Yes	Yes	NR	Yes	No	No	NR	Yes	Low
Meyer 2007<br>Burchall 2017	Yes	Yes	Yes	NR	NR	Yes	Yes	Yes	Yes	Yes	Yes	No	Yes	Yes	Yes	Yes	Yes	Yes	Moderate
Moretti 2018	Yes	Yes	Yes	Yes	Yes	Yes	Yes	Yes	Yes	Yes	Yes	NR	Partial	No	Yes	Yes	Yes	Yes	Moderate
Spritzer 2000	Yes	Yes	NR	NR	NR	NR	Yes	NR	Yes	Yes	Yes	Yes	NR	Yes	N/A	NR	NR	Yes	Moderate
Tartagni 2000	Yes	No	Yes	NR	No	NR	Yes	NR	Yes	Yes	Yes	Yes	Yes	Yes	NR	NR	NR	Yes	High
Tartagni 2004	Yes	No	Yes	NR	NR	NR	Yes	NR	Yes	Yes	NR	Yes	Yes	Yes	NR	NR	NR	Yes	High
Tartagni 2014	Partial	Partial	Yes	NR	NR	NR	Yes	NR	Yes	Yes	Yes	Yes	No	Yes	NR	No	NR	Yes	High
Vieira 2012	Yes	Yes	Yes	No	No	No	Yes	No	Yes	Yes	Yes	Yes	Yes	Yes	NR	No	Yes	Yes	Moderate

*High RoB due to questionable units reported, and uncertainty in the BMI of the sample population. **COI,** conflict of interest; **ITT**, intention to treat; **N/A**, not applicable; **NR**, not reported; **RoB**, risk of bias.

Supplementary File 2

Supplementary Table S2: Results of meta-analysis for anti-androgens (AA) + lifestyle (LS) vs placebo + lifestyle - adults

Outcome	Time-point	RCTs	N	Statistical method	Effect size	P value	Favours	Certainty
BMI	6–12 months	2	89	MD (IV, random, 95% CI)	-3.08 kg/m^2 [-8.67, 2.50]	0.28	None	⊕◯◯◯ VERY LOW
Hirsutism	6–12 months	2	89	MD (IV, random, 95% CI)	-0.93 [-3.37, 1.51]	0.45	None	⊕◯◯◯ VERY LOW
SHBG	6–12 months	2	89	MD (IV, random, 95% CI)	9.72 nmol/l [-0.71, 20.14]	0.07	None	⊕◯◯◯ VERY LOW
Weight	12 months	1	36	MD (IV, random, 95% CI)	-17.00 kg [-25.37, -8.63]	**<0.0001**	AA + LS	⊕⊕◯◯ LOW
FAI	12 months	1	36	MD (IV, random, 95% CI)	-0.80 [-2.34, 0.74]	0.31	None	⊕⊕◯◯ LOW
Testosterone	12 months	1	36	MD (IV, random, 95% CI)	0.05 ng/ml [-0.05, 0.15]	0.34	None	⊕⊕◯◯ LOW
DHEAS	12 months	1	36	MD (IV, random, 95% CI)	-2.34 µg/ml [-4.06, -0.62]	**0.008**	AA + LS	⊕⊕◯◯ LOW
Androstenedione	12 months	1	36	MD (IV, random, 95% CI)	-18.00 ng/ml [-74.86. 38.86]	0.53	None	⊕⊕◯◯ LOW
Fasting insulin	12 months	1	36	MD (IV, random, 95% CI)	-4.00 µU/ml [-6.98, -1.02]	**0.009**	AA + LS	⊕⊕◯◯ LOW
HOMA-IR	6 months	1	20	MD (IV, random, 95% CI)	1.60 [-1.64, 4.84]	0.33	None	⊕⊕◯◯ LOW
Fasting glucose	12 months	1	36	MD (IV, random, 95% CI)	0.00 mg/ml [-5.24, 5.24]	1.00	None	⊕⊕◯◯ LOW
QUICKI	12 months	1	36	MD (IV, random, 95% CI)	0.04 [0.02, 0.06]	**<0.00001**	AA + LS	⊕⊕◯◯ LOW
Insulin sensitivity index	12 months	1	36	MD (IV, random, 95% CI)	5.10 [2.32, 7.88]	**0.0003**	AA + LS	⊕⊕◯◯ LOW
HDL	12 months	1	36	MD (IV, random, 95% CI)	5.00 mg/dl [-1.54, 11.54]	0.13	None	⊕⊕◯◯ LOW
LDL	12 months	1	36	MD (IV, random, 95% CI)	-21.00 mg/dl [-40.93, -1.07]	**0.04**	AA + LS	⊕⊕◯◯ LOW
Triglycerides	12 months	1	36	MD (IV, random, 95% CI)	-50.00 mg/dl [-77.60, -22.40]	**0.0004**	AA + LS	⊕⊕◯◯ LOW
Frequency of menstruation	12 months	1	36	MD (IV, random, 95% CI)	-0.80 [-1.54, -0.06]	**0.03**	AA + LS	⊕⊕◯◯ LOW

AA, anti-androgen; **BMI**, body mass index; **CI**, confidence interval; **DHEAS**, dehydroepiandrosterone sulphate; **FAI**, free androgen index; **HDL**, high-density lipoprotein; **HOMA-IR**, homeostatic model assessment of insulin resistance; **IV**, inverse variance; **LDL**, low density lipoprotein; **LS**, lifestyle; **MD**, mean difference; **QUICKI**, quantitative insulin sensitivity check index; **SHBG**, sex-hormone binding globulin; **WHR**, waist-hip ratio.

Supplementary File 2

Supplementary Figure S1: Forest plots for meta-analysis for pooled outcomes of BMI (A), hirsutism (B), SHBG (C) for anti-androgen + lifestyle vs placebo + lifestyle – adults

(A) BMI

	Anti-androgen + LS			Placebo + LS				Mean Difference
Study or Subgroup	Mean [kg/m^2]	SD [kg/m^2]	Total	Mean [kg/m^2]	SD [kg/m^2]	Total	Weight	IV, Random, 95% CI
Amiri 2014 (High risk)	28.9	4	27	29.2	3.6	26	51.2%	-0.30 [-2.35, 1.75]
Gambineri 2006 (Low risk)	29	3	17	35	5	19	48.8%	-6.00 [-8.66, -3.34]
**Total (95% CI)**			**44**			**45**	**100.0%**	**-3.08 [-8.67, 2.50]**

Heterogeneity: Tau² = 14.78; Chi² = 11.07, df = 1 (P = 0.0009); I² = 91%
Test for overall effect: Z = 1.08 (P = 0.28)

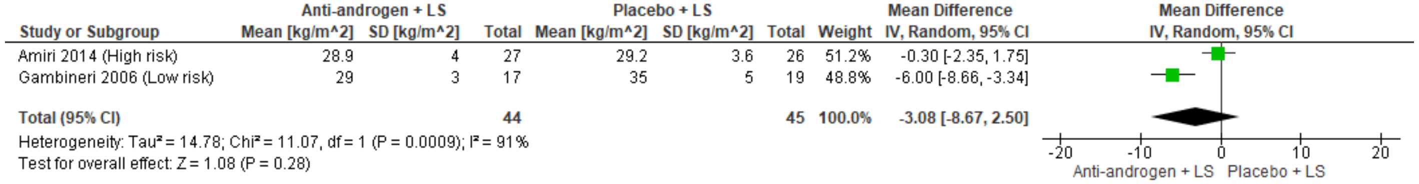

(B) Hirsutism

	Anti-androgen + LS			Placebo + LS				Mean Difference
Study or Subgroup	Mean [FG score]	SD [FG score]	Total	Mean [FG score]	SD [FG score]	Total	Weight	IV, Random, 95% CI
Amiri 2014 (High risk)	5	2.5	27	4.8	2.4	26	54.8%	0.20 [-1.12, 1.52]
Gambineri 2006 (Low risk)	5.7	1.7	17	8	4.1	19	45.2%	-2.30 [-4.31, -0.29]
**Total (95% CI)**			**44**			**45**	**100.0%**	**-0.93 [-3.37, 1.51]**

Heterogeneity: Tau² = 2.37; Chi² = 4.15, df = 1 (P = 0.04); I² = 76%
Test for overall effect: Z = 0.75 (P = 0.45)

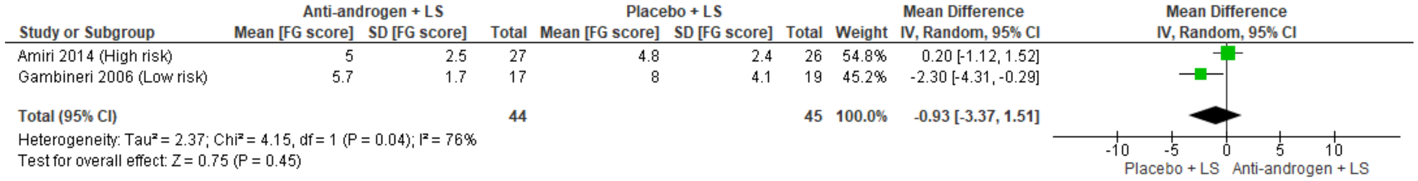

(C) SHBG

	Anti-androgen + LS			Placebo + LS				Mean Difference
Study or Subgroup	Mean [nmol/l]	SD [nmol/l]	Total	Mean [nmol/l]	SD [nmol/l]	Total	Weight	IV, Random, 95% CI
Amiri 2014 (High risk)	41.08	39	27	24.14	11.3	26	35.1%	16.94 [1.60, 32.28]
Gambineri 2006 (Low risk)	28.4	11.3	17	22.6	17.9	19	64.9%	5.80 [-3.88, 15.48]
**Total (95% CI)**			**44**			**45**	**100.0%**	**9.72 [-0.71, 20.14]**

Heterogeneity: Tau² = 19.24; Chi² = 1.45, df = 1 (P = 0.23); I² = 31%
Test for overall effect: Z = 1.83 (P = 0.07)

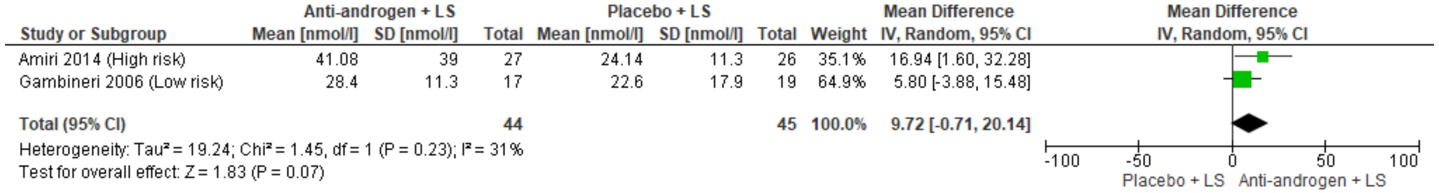

Supplementary File 2

Supplementary Table S3: Results of meta-analyses for anti-androgens (daily) vs anti-androgens (every 3 days) – adults

Outcome	Time-point	RCTs	N	Statistical method	Effect size	P value	Favours	Certainty
Hirsutism	10-12 months	2	80	MD (IV, random, 95% CI)	-3.48 [-4.58, -2.39]	**<0.00001**	Daily AA	⊕◯◯◯ VERY LOW
SHBG	10-12 months	2	80	MD (IV, random, 95% CI)	0.29 nmol/l [-2.18, 2.76]	0.82	None	⊕◯◯◯ VERY LOW
Androstenedione	10-12 months	2	80	MD (IV, random, 95% CI)	-0.30 ng/ml [-0.50, -0.10]	**0.004**	Daily AA	⊕◯◯◯ VERY LOW
Testosterone	10-12 months	2	80	MD (IV, random, 95% CI)	-0.25 ng/ml [-0.73, 0.23]	0.30	None	⊕◯◯◯ VERY LOW
DHEAS	10-12 months	2	80	MD (IV, random, 95% CI)	0.19 µg/ml [-0.79, 1.17]	0.71	None	⊕◯◯◯ VERY LOW
Body mass index	10 months	1	16	MD (IV, random, 95% CI)	0.30 kg/m^2 [-3.67, 4.27]	0.88	None	⊕◯◯◯ VERY LOW
Fasting insulin	12 months	1	64	MD (IV, random, 95% CI)	0.70 µU/ml [-0.17, 1.57]	0.11	None	⊕◯◯◯ VERY LOW
Dry skin	12 months	1	64	OR (IV, random, 95% CI)	6.60 [2.21, 19.73]	**0.0007**	Daily AA	⊕◯◯◯ VERY LOW
Decreased libido	12 months	1	64	OR (IV, random, 95% CI)	1.62 [0.41, 6.38]	0.49	None	⊕◯◯◯ VERY LOW
Headache	12 months	1	64	OR (IV, random, 95% CI)	0.23 [0.02, 2.14]	0.19	None	⊕◯◯◯ VERY LOW

AA, anti-androgen; **CI**, confidence interval; **DHEAS**, dehydroepiandrosterone sulphate; **IV**, inverse variance; **OR**, odds ratio; **RCT**, randomised controlled trial; **SHBG**, sex-hormone binding globulin.

Supplementary File 2

Supplementary Figure S2: Forest plots for meta-analysis for pooled outcomes of hirsutism (A), SHBG (B), androstenedione (C), testosterone (D), DHEAS (E), for anti-androgens (daily) vs anti-androgens (every 3 days) – adults

(A) Hirsutism

Study or Subgroup	Daily anti-androgen Mean [FG score]	SD [FG score]	Total	Every 3d anti-androgen Mean [FG score]	SD [FG score]	Total	Weight	Mean Difference IV, Random, 95% CI
Falsetti 1999 (High risk)	7.1	2.5	32	10.9	2.6	32	75.6%	-3.80 [-5.05, -2.55]
Tartagni 2004 (High risk)	10.4	2.7	8	12.9	1.7	8	24.4%	-2.50 [-4.71, -0.29]
**Total (95% CI)**			**40**			**40**	**100.0%**	**-3.48 [-4.58, -2.39]**

Heterogeneity: Tau² = 0.01; Chi² = 1.01, df = 1 (P = 0.32); I² = 1%
Test for overall effect: Z = 6.24 (P < 0.00001)

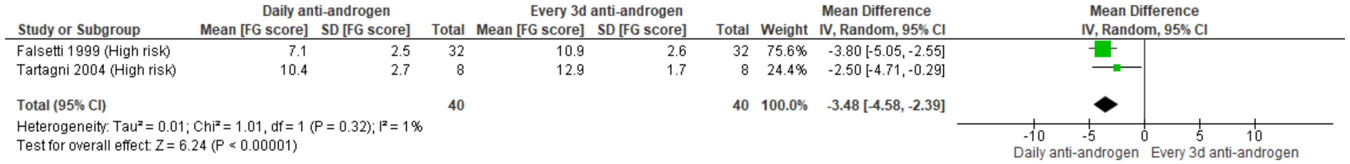

(B) SHBG

Study or Subgroup	Daily anti-androgen Mean [nmol/l]	SD [nmol/l]	Total	Every 3d anti-androgen Mean [nmol/l]	SD [nmol/l]	Total	Weight	Mean Difference IV, Random, 95% CI
Falsetti 1999 (High risk)	21	4.6	32	20.7	5.6	32	96.6%	0.30 [-2.21, 2.81]
Tartagni 2004 (High risk)	20	14.74	8	20	12.63	8	3.4%	0.00 [-13.45, 13.45]
**Total (95% CI)**			**40**			**40**	**100.0%**	**0.29 [-2.18, 2.76]**

Heterogeneity: Tau² = 0.00; Chi² = 0.00, df = 1 (P = 0.97); I² = 0%
Test for overall effect: Z = 0.23 (P = 0.82)

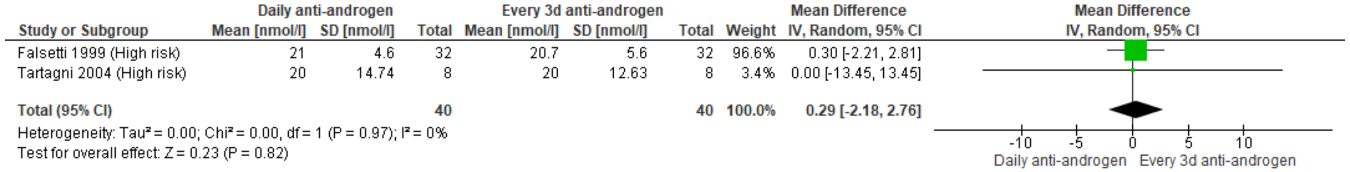

(C) Androstenedione

Study or Subgroup	Daily anti-androgen Mean [ng/ml]	SD [ng/ml]	Total	Every 3d anti-androgen Mean [ng/ml]	SD [ng/ml]	Total	Weight	Mean Difference IV, Random, 95% CI
Falsetti 1999 (High risk)	3.4	0.5	32	3.7	0.3	32	98.2%	-0.30 [-0.50, -0.10]
Tartagni 2004 (High risk)	3.7	1.8	8	3.8	1.2	8	1.8%	-0.10 [-1.60, 1.40]
**Total (95% CI)**			**40**			**40**	**100.0%**	**-0.30 [-0.50, -0.10]**

Heterogeneity: Tau² = 0.00; Chi² = 0.07, df = 1 (P = 0.80); I² = 0%
Test for overall effect: Z = 2.90 (P = 0.004)

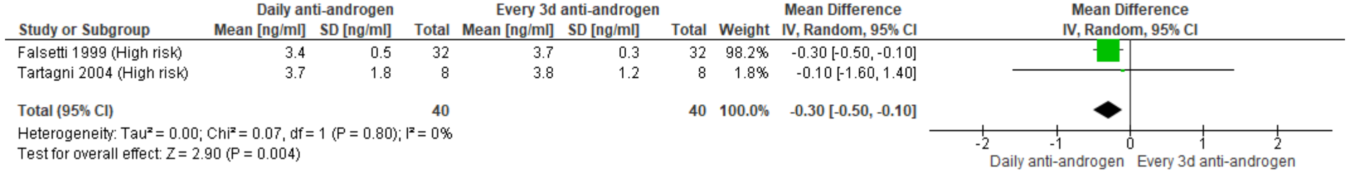

(D) Testosterone

Study or Subgroup	Daily anti-androgen Mean [ng/ml]	SD [ng/ml]	Total	Every 3d anti-androgen Mean [ng/ml]	SD [ng/ml]	Total	Weight	Mean Difference IV, Random, 95% CI
Falsetti 1999 (High risk)	0.9	0.2	32	1.4	0.2	32	49.5%	-0.50 [-0.60, -0.40]
Tartagni 2004 (High risk)	0.76	0.03	8	0.77	0.03	8	50.5%	-0.01 [-0.04, 0.02]
**Total (95% CI)**			**40**			**40**	**100.0%**	**-0.25 [-0.73, 0.23]**

Heterogeneity: Tau² = 0.12; Chi² = 88.11, df = 1 (P < 0.00001); I² = 99%
Test for overall effect: Z = 1.03 (P = 0.30)

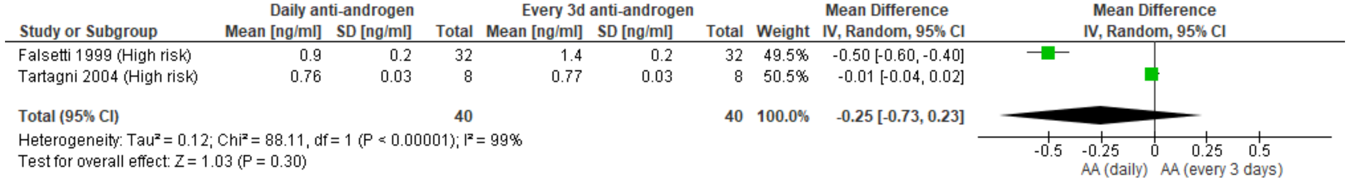

(E) DHEAS

Supplementary File 2

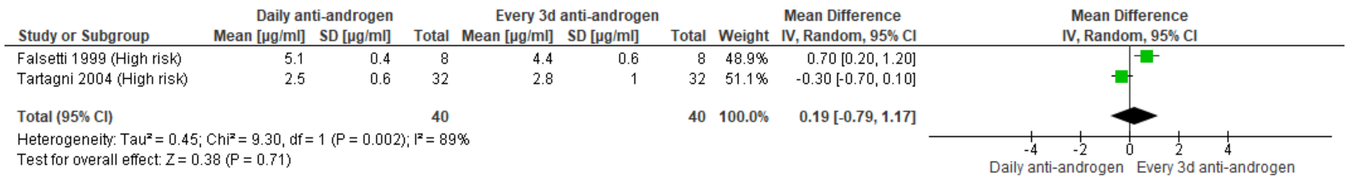

Supplementary Table S4: Results of meta-analysis for anti-androgen + lifestyle vs metformin + lifestyle - adults

Outcome	Time-point	RCTs	N	Statistical method	Effect size	P value	Favours	Certainty
Weight	6-12 months	2	144	MD (IV, random, 95% CI)	-5.64 kg [-19.33, 8.05]	0.42	None	⊕⊕◯◯ LOW
BMI	6-12 months	4	265	MD (IV, random, 95% CI)	-0.79 kg/m^2 [-2.45, 0.87]	0.35	None	⊕◯◯◯ VERY LOW
WHR	6 months	2	176	MD (IV, random, 95% CI)	0.02 [-0.00, 0.04]	0.11	None	⊕⊕⊕◯ MODERATE
Frequency of menstruation	6-12 months	2	176	MD (IV, random, 95% CI)	0.79 cycles/year [0.05, 1.53]	**0.04**	AA + LS	⊕⊕⊕◯ MODERATE
Hirsutism	6-12 months	4	265	MD (IV, random, 95% CI)	-1.59 [-3.06, -0.12]	**0.03**	AA + LS	⊕◯◯◯ VERY LOW
SHBG	6-12 months	2	89	MD (IV, random, 95% CI)	7.70 nmol/l [0.75, 14.66]	**0.03**	AA + LS	⊕◯◯◯ VERY LOW
Testosterone	6-12 months	3	213	MD (IV, random, 95% CI)	0.03 nmol/l [-0.21, 0.27]	0.81	None	⊕⊕⊕◯ MODERATE
DHEAS	6-12 months	2	106	MD (IV, random, 95% CI)	-1.02 µmol/L [-3.27, 1.24]	0.38	None	⊕◯◯◯ VERY LOW
Fasting insulin	6-12 months	3	213	MD (IV, random, 95% CI)	-2.11 µU/ml [-3.97, -0.26]	**0.03**	AA + LS	⊕⊕⊕◯ MODERATE
Fasting glucose	6-12 months	3	228	MD (IV, random, 95% CI)	0.44 mg/dl [-2.14, 3.02]	0.74	None	⊕⊕◯◯ LOW
Fasting glucose-insulin ratio	6 months	2	176	MD (IV, random, 95% CI)	-1.12 [-1.44, -0.79]	**<0.00001**	AA + LS	⊕⊕⊕◯ MODERATE
QUICKI	6 months	2	144	MD (IV, random, 95% CI)	0.01 [-0.02, 0.04]	0.35	None	⊕⊕⊕◯ MODERATE
FAI	12 months	1	37	MD (IV, random, 95% CI)	-0.60 [-1.99, 0.79]	0.40	None	⊕⊕◯◯ LOW
Androstenedione	12 months	1	37	MD (IV, random, 95% CI)	-39.00 ng/dl [-123.43, -0.25]	0.37	None	⊕⊕◯◯ LOW
OGTT 2-hr glucose	6 months	1	52	MD (IV, random, 95% CI)	-9.54 mg/dl [-23.70, 4.62]	0.19	None	⊕◯◯◯ VERY LOW
HOMA-IR	6 months	1	69	MD (IV, random, 95% CI)	2.72 [-0.28, 5.72]	0.08	None	⊕⊕◯◯ LOW
Total cholesterol	6 months	1	52	MD (IV, random, 95% CI)	7.20 mmol/l [-13.06, 27.46]	0.49	None	⊕◯◯◯ VERY LOW

Supplementary File 2

HDL	12 months	1	37	MD (IV, random, 95% CI)	0.21 mmol/l [0.05, 0.37]	**0.01**	AA + LS	⊕⊕◯◯ LOW
LDL	12 months	1	37	MD (IV, random, 95% CI)	-0.29 mmol/l [-0.83, 0.25]	0.29	None	⊕⊕◯◯ LOW
Triglycerides	12 months	1	37	MD (IV, random, 95% CI)	-0.23 mmol/l [-0.51, 0.05]	0.10	None	⊕⊕◯◯ LOW

AA, anti-androgen; **BMI**, body mass index; **CI**, confidence interval; **DHEAS**, dehydroepiandrosterone sulphate; **FAI**, free androgen index; **HDL**, high-density lipoprotein; **HOMA-IR**, homeostatic model assessment of insulin resistance; **IV**, inverse variance; **LDL**, low density lipoprotein; **LS**, lifestyle; **MD**, mean difference; **OGTT**, oral glucose tolerance test; **QUICKI**, quantitative insulin sensitivity check index; **SHBG**, sex-hormone binding globulin; **WHR**, waist-hip ratio.

Supplementary Figure S3: Forest plots for meta-analysis for pooled outcomes of weight (A), BMI (B), WHR (C), frequency of menstruation (D), hirsutism (E), SHBG (F), testosterone (G), DHEAS (H), fasting insulin (I), fasting glucose (J), fasting glucose: insulin ratio (K), QUICKI (L), for anti-androgens + lifestyle vs metformin + lifestyle – adults

(A) Weight

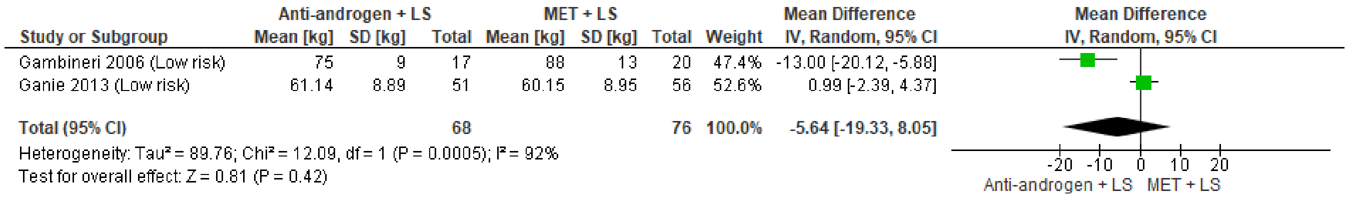

(B) BMI

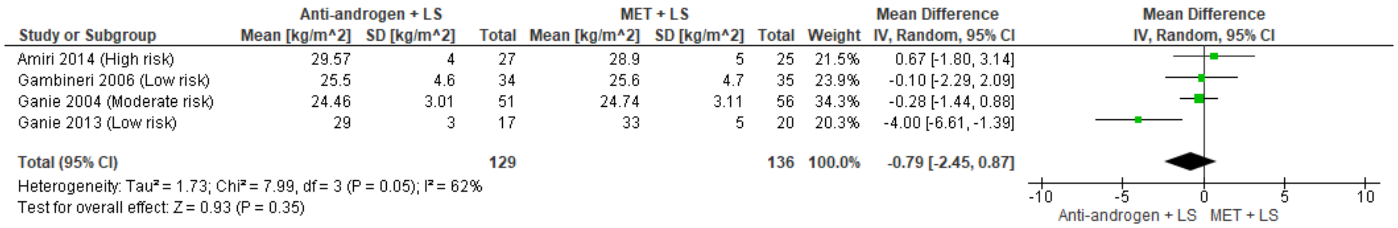

(C) WHR

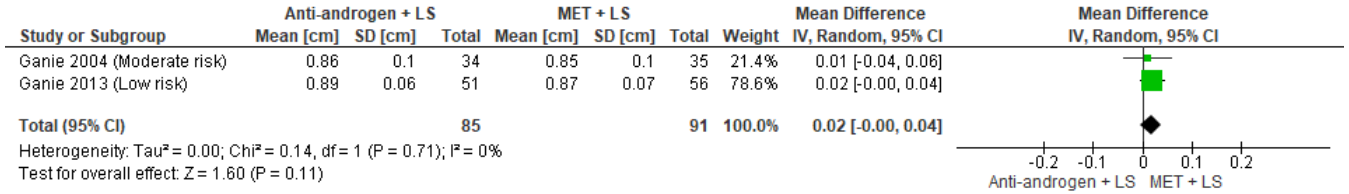

Supplementary File 2

(D) Frequency of menstruation (cycles/year)

Study or Subgroup	Anti-androgen + LS Mean [cycles/y]	Anti-androgen + LS SD [cycles/y]	Anti-androgen + LS Total	MET + LS Mean [cycles/y]	MET + LS SD [cycles/y]	MET + LS Total	Weight	Mean Difference IV, Random, 95% CI
Ganie 2004 (Moderate risk)	10.2	1.9	34	9.1	2	35	59.4%	1.10 [0.18, 2.02]
Ganie 2013 (Low risk)	10.35	2.8	51	10.02	3.16	56	40.6%	0.33 [-0.80, 1.46]
**Total (95% CI)**			**85**			**91**	**100.0%**	**0.79 [0.05, 1.53]**
Heterogeneity: Tau² = 0.02; Chi² = 1.07, df = 1 (P = 0.30); I² = 7%								
Test for overall effect: Z = 2.08 (P = 0.04)								

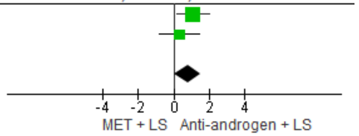

(E) Hirsutism

Study or Subgroup	Anti-androgen + LS Mean [FG score]	Anti-androgen + LS SD [FG score]	Anti-androgen + LS Total	MET + LS Mean [FG score]	MET + LS SD [FG score]	MET + LS Total	Weight	Mean Difference IV, Random, 95% CI
Amiri 2014 (High risk)	5	2.5	27	7.08	3.8	25	24.0%	-2.08 [-3.84, -0.32]
Gambineri 2006 (Low risk)	5.7	1.7	17	10.4	6.6	20	14.5%	-4.70 [-7.70, -1.70]
Ganie 2004 (Moderate risk)	8.7	1.9	34	10	3.3	35	28.8%	-1.30 [-2.57, -0.03]
Ganie 2013 (Low risk)	9.56	2.29	51	9.67	2.19	56	32.6%	-0.11 [-0.96, 0.74]
**Total (95% CI)**			**129**			**136**	**100.0%**	**-1.59 [-3.06, -0.12]**
Heterogeneity: Tau² = 1.54; Chi² = 11.56, df = 3 (P = 0.009); I² = 74%								
Test for overall effect: Z = 2.12 (P = 0.03)								

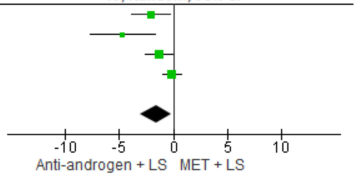

(F) SHBG

Study or Subgroup	Anti-androgen + LS Mean [nmol/l]	Anti-androgen + LS SD [nmol/l]	Anti-androgen + LS Total	MET + LS Mean [nmol/l]	MET + LS SD [nmol/l]	MET + LS Total	Weight	Mean Difference IV, Random, 95% CI
Amiri 2014 (High risk)	41.08	39	27	26.9	18.9	25	17.8%	14.18 [-2.29, 30.65]
Gambineri 2006 (Low risk)	28.4	11.3	17	22.1	12.5	20	82.2%	6.30 [-1.37, 13.97]
**Total (95% CI)**			**44**			**45**	**100.0%**	**7.70 [0.75, 14.66]**
Heterogeneity: Tau² = 0.00; Chi² = 0.72, df = 1 (P = 0.40); I² = 0%								
Test for overall effect: Z = 2.17 (P = 0.03)								

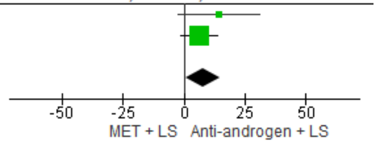

(G) Testosterone

Study or Subgroup	Anti-androgen + LS Mean [nmol/l]	Anti-androgen + LS SD [nmol/l]	Anti-androgen + LS Total	MET + LS Mean [nmol/l]	MET + LS SD [nmol/l]	MET + LS Total	Weight	Mean Difference IV, Random, 95% CI
Gambineri 2006 (Low risk)	1.73	0.59	17	1.73	0.94	20	23.5%	0.00 [-0.50, 0.50]
Ganie 2004 (Moderate risk)	1.94	1	34	1.7	0.86	35	30.0%	0.24 [-0.20, 0.68]
Ganie 2013 (Low risk)	1.8	1.11	51	1.89	0.69	56	46.5%	-0.09 [-0.44, 0.26]
**Total (95% CI)**			**102**			**111**	**100.0%**	**0.03 [-0.21, 0.27]**
Heterogeneity: Tau² = 0.00; Chi² = 1.33, df = 2 (P = 0.51); I² = 0%								
Test for overall effect: Z = 0.25 (P = 0.81)								

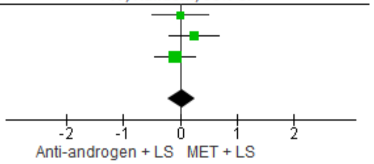

(H) DHEAS

Supplementary File 2

Study or Subgroup	Anti-androgen + LS Mean [µmol/l]	Anti-androgen + LS SD [µmol/l]	Anti-androgen + LS Total	MET + LS Mean [µmol/l]	MET + LS SD [µmol/l]	MET + LS Total	Weight	Mean Difference IV, Random, 95% CI
Gambineri 2006 (Low risk)	4.071	1.9	17	6.2422	1.63	20	49.8%	-2.17 [-3.32, -1.02]
Ganie 2004 (Moderate risk)	6.23	2	34	6.1	2.7	35	50.2%	0.13 [-0.99, 1.25]
**Total (95% CI)**			**51**			**55**	**100.0%**	**-1.02 [-3.27, 1.24]**

Heterogeneity: $Tau^2 = 2.31$; $Chi^2 = 7.89$, df = 1 (P = 0.005); $I^2 = 87\%$
Test for overall effect: Z = 0.88 (P = 0.38)

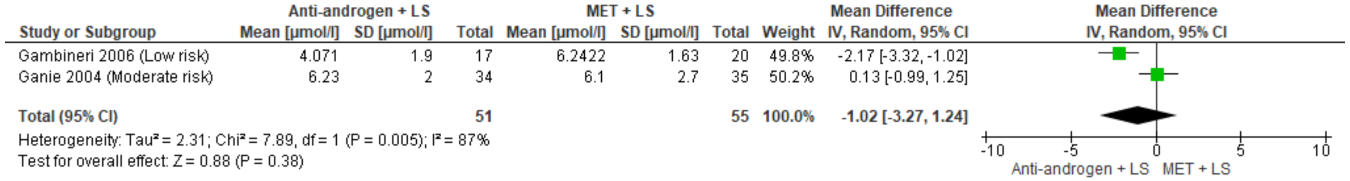

Supplementary File 2

(I) Fasting insulin

Study or Subgroup	Anti-androgen + LS Mean [µU/ml]	Anti-androgen + LS SD [µU/ml]	Anti-androgen + LS Total	MET + LS Mean [µU/ml]	MET + LS SD [µU/ml]	MET + LS Total	Weight	Mean Difference IV, Random, 95% CI
Gambineri 2006 (Low risk)	7	5	17	10	5	20	32.8%	-3.00 [-6.23, 0.23]
Ganie 2004 (Moderate risk)	10.37	5.7	34	14.28	17.5	35	9.2%	-3.91 [-10.02, 2.20]
Ganie 2013 (Low risk)	9.18	5.56	51	10.51	7.22	56	58.0%	-1.33 [-3.76, 1.10]
**Total (95% CI)**			**102**			**111**	**100.0%**	**-2.11 [-3.97, -0.26]**

Heterogeneity: Tau² = 0.00; Chi² = 1.02, df = 2 (P = 0.60); I² = 0%
Test for overall effect: Z = 2.24 (P = 0.03)

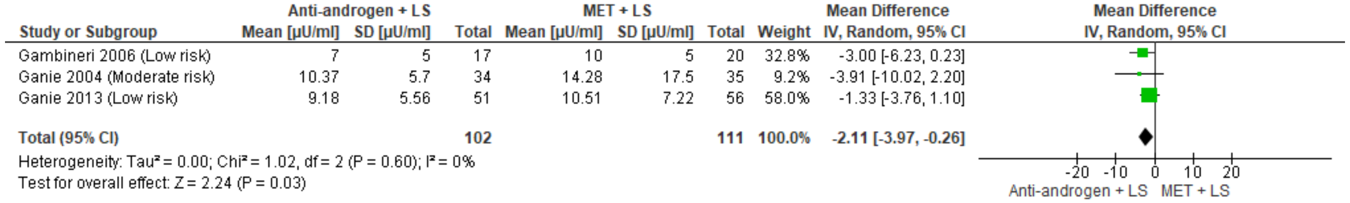

(J) Fasting glucose

Study or Subgroup	Anti-androgen + LS Mean [mg/dl]	Anti-androgen + LS SD [mg/dl]	Anti-androgen + LS Total	MET + LS Mean [mg/dl]	MET + LS SD [mg/dl]	MET + LS Total	Weight	Mean Difference IV, Random, 95% CI
Amiri 2014 (High risk)	83.7	7.2	27	81.9	8.1	25	38.2%	1.80 [-2.38, 5.98]
Ganie 2004 (Moderate risk)	93.8	12.4	34	95.9	12.9	35	18.7%	-2.10 [-8.07, 3.87]
Ganie 2013 (Low risk)	88.78	8.84	51	88.44	11.83	56	43.1%	0.34 [-3.60, 4.28]
**Total (95% CI)**			**112**			**116**	**100.0%**	**0.44 [-2.14, 3.02]**

Heterogeneity: Tau² = 0.00; Chi² = 1.10, df = 2 (P = 0.58); I² = 0%
Test for overall effect: Z = 0.33 (P = 0.74)

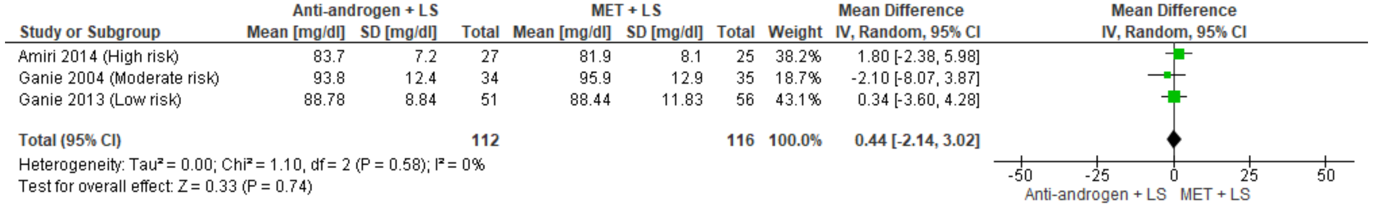

(K) Fasting glucose-insulin ratio

Study or Subgroup	Anti-androgen + LS Mean [mg/dl]	Anti-androgen + LS SD [mg/dl]	Anti-androgen + LS Total	MET + LS Mean [mg/dl]	MET + LS SD [mg/dl]	MET + LS Total	Weight	Mean Difference IV, Random, 95% CI
Ganie 2004 (Moderate risk)	9.7	0.69	34	10.83	0.69	35	99.0%	-1.13 [-1.46, -0.80]
Ganie 2013 (Low risk)	11.85	8.61	51	11.85	8.61	56	1.0%	0.00 [-3.27, 3.27]
**Total (95% CI)**			**85**			**91**	**100.0%**	**-1.12 [-1.44, -0.79]**

Heterogeneity: Tau² = 0.00; Chi² = 0.46, df = 1 (P = 0.50); I² = 0%
Test for overall effect: Z = 6.77 (P < 0.00001)

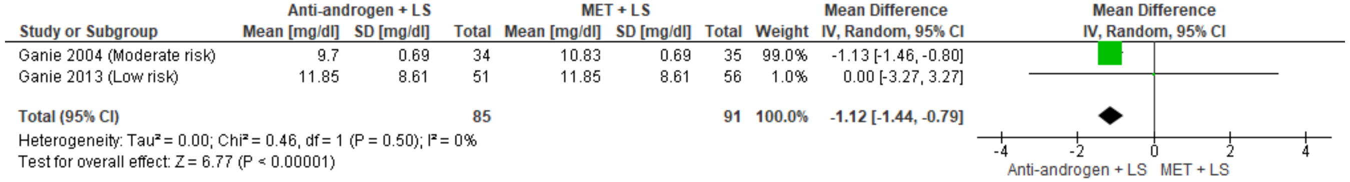

(L) QUICKI

Study or Subgroup	Anti-androgen + LS Mean [mg/dl]	Anti-androgen + LS SD [mg/dl]	Anti-androgen + LS Total	MET + LS Mean [mg/dl]	MET + LS SD [mg/dl]	MET + LS Total	Weight	Mean Difference IV, Random, 95% CI
Gambineri 2006 (Low risk)	0.38	0.03	17	0.35	0.03	20	46.4%	0.03 [0.01, 0.05]
Ganie 2013 (Low risk)	0.35	0.03	51	0.35	0.03	56	53.6%	0.00 [-0.01, 0.01]
**Total (95% CI)**			**68**			**76**	**100.0%**	**0.01 [-0.02, 0.04]**

Heterogeneity: Tau² = 0.00; Chi² = 6.84, df = 1 (P = 0.009); I² = 85%
Test for overall effect: Z = 0.93 (P = 0.35)

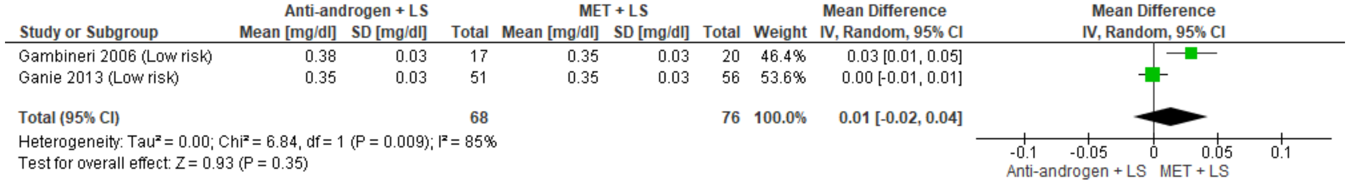

Supplementary File 2

Supplementary File 2

Supplementary Table S5: Results of meta-analyses for anti-androgens + metformin + lifestyle vs metformin + lifestyle - adults

Outcome	Time-point	RCTs	N	Statistical method	Effect size	P value	Favours	Certainty
Weight	6-12 months	3	210	MD (IV, random, 95% CI)	-0.92 kg [-7.30, 5.45]	0.78	None	⊕⊕◯◯ LOW
BMI	6-12 months	4	262	MD (IV, random, 95% CI)	-0.20 kg/m^2 [-1.43, 1.02]	0.74	None	⊕⊕◯◯ LOW
WHR	6 months	2	170	MD (IV, random, 95% CI)	-0.00 [-0.06, 0.06]	0.93	None	⊕◯◯◯ VERY LOW
Hirsutism	6-12 months	4	262	MD (IV, random, 95% CI)	-0.58 [-1.86, 0.69]	0.37	None	⊕⊕◯◯ LOW
SHBG	6-12 months	3	144	MD (IV, random, 95% CI)	-0.59 nmol/l [-4.52, 3.34]	0.77	None	⊕⊕◯◯ LOW
Testosterone	6-12 months	4	262	MD (IV, random, 95% CI)	-0.29 nmol/l [-0.52, -0.06]	**0.01**	AA + MET + LS	⊕◯◯◯ VERY LOW
DHEAS	6-12 months	2	92	MD (IV, random, 95% CI)	-0.24 μg/ml [-0.92, 0.45]	0.50	None	⊕⊕⊕◯ MODERATE
Fasting insulin	6-12 months	3	210	MD (IV, random, 95% CI)	-1.22 μU/ml [-2.87, 0.43]	0.15	None	⊕⊕⊕⊕ HIGH
Fasting glucose	6-12 months	4	262	MD (IV, random, 95% CI)	-2.93 mg/dl [-5.78, -0.09]	**0.04**	AA + MET + LS	⊕⊕◯◯ LOW
HDL	6-12 months	2	92	MD (IV, random, 95% CI)	1.75 mg/dl [-7.72, 11.23]	0.72	None	⊕⊕◯◯ LOW
LDL	6-12 months	2	92	MD (IV, random, 95% CI)	-8.24 mg/dl [-20.34, 3.86]	0.18	None	⊕⊕⊕◯ MODERATE
Triglycerides	6-12 months	2	92	MD (IV, random, 95% CI)	-5.48 mg/dl [-24.06, 13.10]	0.56	None	⊕⊕⊕◯ MODERATE
HOMA-IR	6 months	2	170	MD (IV, random, 95% CI)	-0.22 [-0.67, 0.23]	0.34	None	⊕⊕⊕⊕ HIGH
Frequency of menstruation	6 months	1	118	MD (IV, random, 95% CI)	0.84 cycles/year [-0.31, 1.99]	0.15	None	⊕⊕⊕⊕ HIGH
FAI	12 months	1	52	MD (IV, random, 95% CI)	-2.10 [-4.47, 0.27]	0.08	None	⊕⊕⊕◯ MODERATE
Androstenedione	12 months	1	40	MD (IV, random, 95% CI)	-5.00 ng/dl [-96.42, 86.42]	0.91	None	⊕⊕◯◯ LOW
Fasting glucose-insulin ratio	6 months	1	118	MD (IV, random, 95% CI)	1.74 [-1.45, 4.93]	0.28	None	⊕⊕⊕⊕ HIGH
QUICKI	12 months	1	40	MD (IV, random, 95% CI)	0.01 [-0.01, 0.03]	0.29	None	⊕⊕◯◯ LOW
OGTT	6 months	1	52	MD (IV, random, 95% CI)	-4.88 mg/dl [-20.32, 10.56]	0.54	None	⊕⊕◯◯ LOW
Insulin sensitivity index	12 months	1	40	MD (IV, random, 95% CI)	6.90 [2.96, 10.84]	**0.0006**	AA + MET + LS	⊕⊕◯◯ LOW
Total cholesterol	6 months	1	52	MD (IV, random, 95% CI)	0.30 mg/dl [-16.76, 17.36]	0.97	None	⊕⊕⊕◯ MODERATE

Supplementary File 2

AA, anti-androgen; **BMI**, body mass index; **CI**, confidence interval; **DHEAS**, dehydroepiandrosterone sulphate; **FAI**, free androgen index; **HDL**, high-density lipoprotein; **HOMA-IR**, homeostatic model assessment of insulin resistance; **IV**, inverse variance; **LDL**, low density lipoprotein; **LS**, lifestyle; **MD**, mean difference; **OGTT**, oral glucose tolerance test; **QUICKI**, quantitative insulin sensitivity check index; **SHBG**, sex-hormone binding globulin; **WHR**, waist-hip ratio.

Supplementary File 2

Supplementary Figure S4: Forest plots for meta-analysis for pooled outcomes of weight (A), BMI (B), WHR (C), hirsutism (D), SHBG (E), testosterone (F), DHEAS (G), fasting insulin (H), fasting glucose (I), HDL (J), LDL (K), triglycerides (L) and HOMA-IR (M), for anti-androgen + metformin + lifestyle vs metformin + lifestyle – adults.

.

(A) Weight

	Anti-androgen + MET + LS			MET + LS				Mean Difference
**Study or Subgroup**	**Mean [kg]**	**SD [kg]**	**Total**	**Mean [kg]**	**SD [kg]**	**Total**	**Weight**	**IV, Random, 95% CI**
Gambineri 2006 (Low risk)	79	14	20	88	13	20	26.8%	-9.00 [-17.37, -0.63]
Ganie 2013 (Low risk)	60.03	7.53	62	60.15	8.95	56	44.8%	-0.12 [-3.12, 2.88]
Mazza 2014 (Low risk)	77.7	14.8	26	72.3	13.9	26	28.5%	5.40 [-2.40, 13.20]
**Total (95% CI)**			**108**			**102**	**100.0%**	**-0.92 [-7.30, 5.45]**

Heterogeneity: Tau² = 21.32; Chi² = 6.21, df = 2 (P = 0.04); I² = 68%
Test for overall effect: Z = 0.28 (P = 0.78)

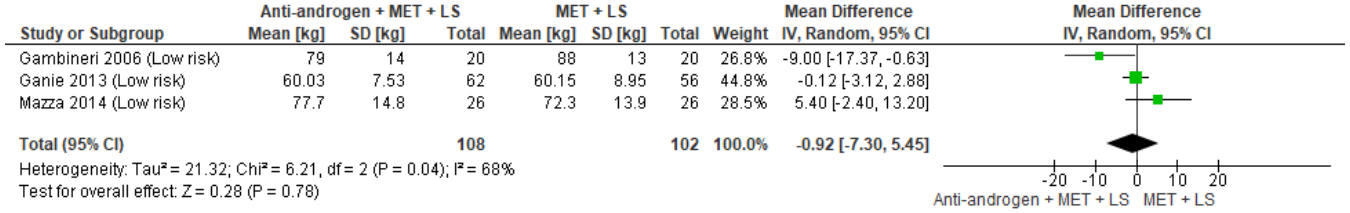

(B) BMI

	Anti-androgen + MET + LS			MET + LS				Mean Difference
**Study or Subgroup**	**Mean [kg/m^2]**	**SD [kg/m^2]**	**Total**	**Mean [kg/m^2]**	**SD [kg/m^2]**	**Total**	**Weight**	**IV, Random, 95% CI**
Amiri 2014 (High risk)	29.3	2.6	27	28.9	5	25	22.8%	0.40 [-1.79, 2.59]
Gambineri 2006 (Low risk)	29.5	5.4	26	27.7	4.7	26	16.1%	1.80 [-0.95, 4.55]
Ganie 2013 (Low risk)	24.07	3.36	62	24.74	3.11	56	47.9%	-0.67 [-1.84, 0.50]
Mazza 2014 (Low risk)	31	5	20	33	5	20	13.2%	-2.00 [-5.10, 1.10]
**Total (95% CI)**			**135**			**127**	**100.0%**	**-0.20 [-1.43, 1.02]**

Heterogeneity: Tau² = 0.46; Chi² = 4.17, df = 3 (P = 0.24); I² = 28%
Test for overall effect: Z = 0.33 (P = 0.74)

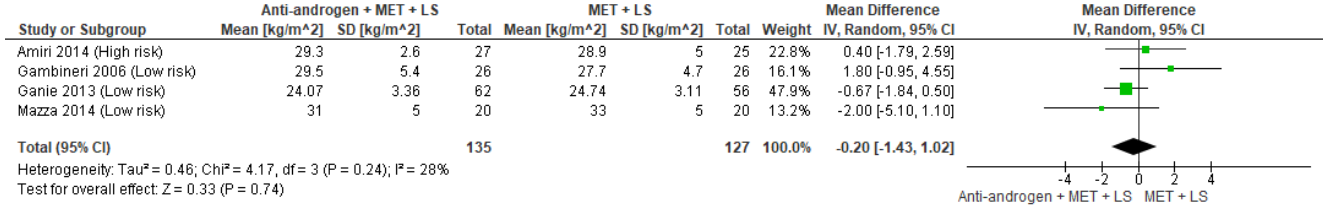

(C) WHR

	Anti-androgen + MET + LS			MET + LS				Mean Difference
**Study or Subgroup**	**Mean [cm]**	**SD [cm]**	**Total**	**Mean [cm]**	**SD [cm]**	**Total**	**Weight**	**IV, Random, 95% CI**
Amiri 2014 (High risk)	0.83	0.04	27	0.8	0.1	25	45.9%	0.03 [-0.01, 0.07]
Ganie 2013 (Low risk)	0.84	0.07	62	0.87	0.07	56	54.1%	-0.03 [-0.06, -0.00]
**Total (95% CI)**			**89**			**81**	**100.0%**	**-0.00 [-0.06, 0.06]**

Heterogeneity: Tau² = 0.00; Chi² = 5.75, df = 1 (P = 0.02); I² = 83%
Test for overall effect: Z = 0.08 (P = 0.93)

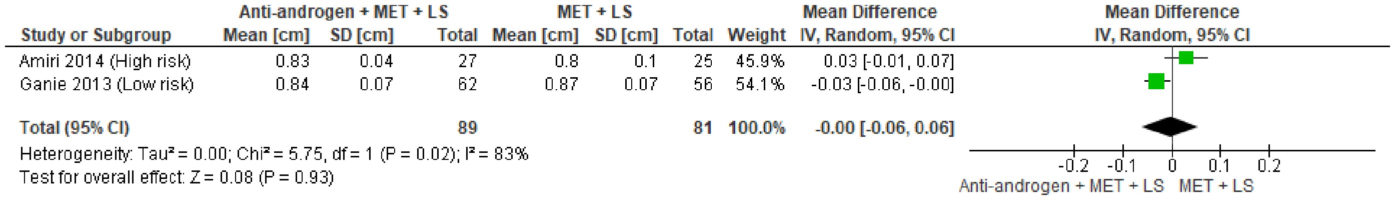

(D) Hirsutism

	Anti-androgen + MET + LS			MET + LS				Mean Difference
**Study or Subgroup**	**Mean [FG score]**	**SD [FG score]**	**Total**	**Mean [FG score]**	**SD [FG score]**	**Total**	**Weight**	**IV, Random, 95% CI**
Amiri 2014 (High risk)	7.52	3.8	27	7.08	3.8	25	23.4%	0.44 [-1.63, 2.51]
Gambineri 2006 (Low risk)	6.5	3.9	20	10.4	6.6	20	11.6%	-3.90 [-7.26, -0.54]
Ganie 2013 (Low risk)	9.09	2.29	62	9.67	2.19	56	48.6%	-0.58 [-1.39, 0.23]
Mazza 2014 (Low risk)	11	5	26	10.7	4.9	26	16.4%	0.30 [-2.39, 2.99]
**Total (95% CI)**			**135**			**127**	**100.0%**	**-0.58 [-1.86, 0.69]**

Heterogeneity: Tau² = 0.70; Chi² = 5.09, df = 3 (P = 0.17); I² = 41%
Test for overall effect: Z = 0.90 (P = 0.37)

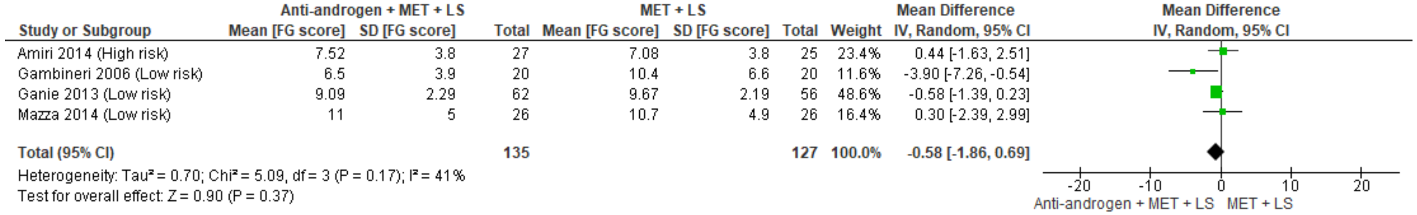

Supplementary File 2

(E) SHBG

Study or Subgroup	Anti-androgen + MET + LS Mean [nmol/l]	SD [nmol/l]	Total	MET + LS Mean [nmol/l]	SD [nmol/l]	Total	Weight	Mean Difference IV, Random, 95% CI
Amiri 2014 (High risk)	22.64	9.7	27	26.9	18.9	25	20.6%	-4.26 [-12.52, 4.00]
Gambineri 2006 (Low risk)	26.4	12.9	20	22.1	12.5	20	22.4%	4.30 [-3.57, 12.17]
Mazza 2014 (Low risk)	24.3	8.5	26	25.5	8	26	57.0%	-1.20 [-5.69, 3.29]
**Total (95% CI)**			**73**			**71**	**100.0%**	**-0.59 [-4.53, 3.34]**

Heterogeneity: Tau² = 1.84; Chi² = 2.31, df = 2 (P = 0.32); I² = 13%
Test for overall effect: Z = 0.30 (P = 0.77)

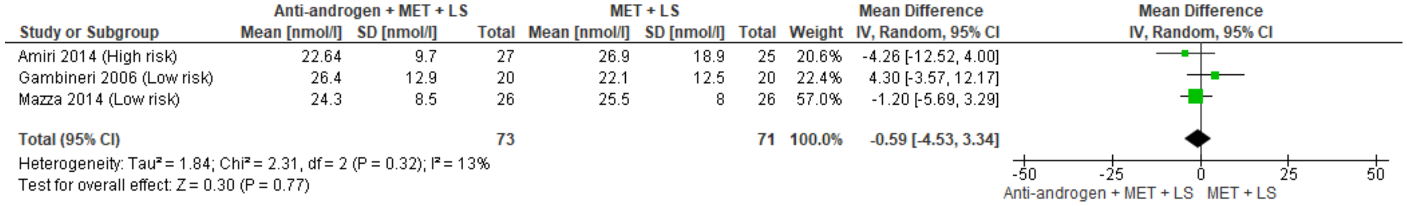

(F) Testosterone

Study or Subgroup	Anti-androgen + MET + LS Mean [nmol/l]	SD [nmol/l]	Total	MET + LS Mean [nmol/l]	SD [nmol/l]	Total	Weight	Mean Difference IV, Random, 95% CI
Amiri 2014 (High risk)	0.6	0.06	27	0.7	0.4	25	35.7%	-0.10 [-0.26, 0.06]
Gambineri 2006 (Low risk)	1.49	0.69	20	1.73	0.94	20	13.5%	-0.24 [-0.75, 0.27]
Ganie 2013 (Low risk)	1.58	0.74	62	1.89	0.69	56	27.7%	-0.31 [-0.57, -0.05]
Mazza 2014 (Low risk)	1.79	0.47	26	2.38	0.69	26	23.1%	-0.59 [-0.91, -0.27]
**Total (95% CI)**			**135**			**127**	**100.0%**	**-0.29 [-0.52, -0.06]**

Heterogeneity: Tau² = 0.03; Chi² = 7.78, df = 3 (P = 0.05); I² = 61%
Test for overall effect: Z = 2.51 (P = 0.01)

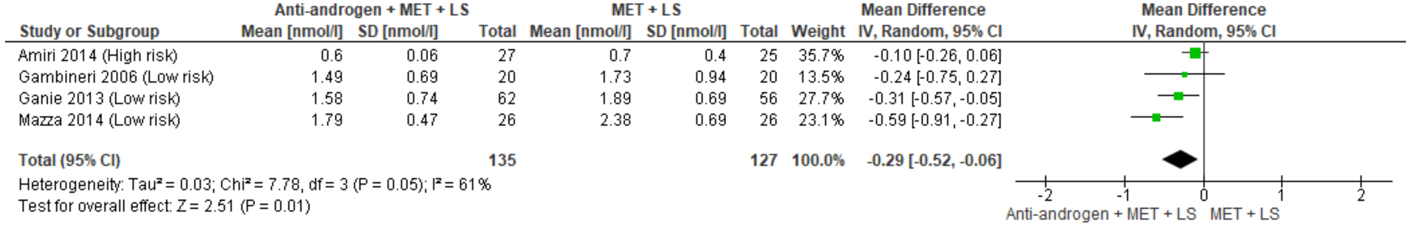

(G) DHEAS

Study or Subgroup	Anti-androgen + MET + LS Mean [µg/ml]	SD [µg/ml]	Total	MET + LS Mean [µg/ml]	SD [µg/ml]	Total	Weight	Mean Difference IV, Random, 95% CI
Gambineri 2006 (Low risk)	1.7	1.2	20	2.3	0.6	20	47.9%	-0.60 [-1.19, -0.01]
Mazza 2014 (Low risk)	2.2	0.9	26	2.1	1	26	52.1%	0.10 [-0.42, 0.62]
**Total (95% CI)**			**46**			**46**	**100.0%**	**-0.24 [-0.92, 0.45]**

Heterogeneity: Tau² = 0.17; Chi² = 3.07, df = 1 (P = 0.08); I² = 67%
Test for overall effect: Z = 0.67 (P = 0.50)

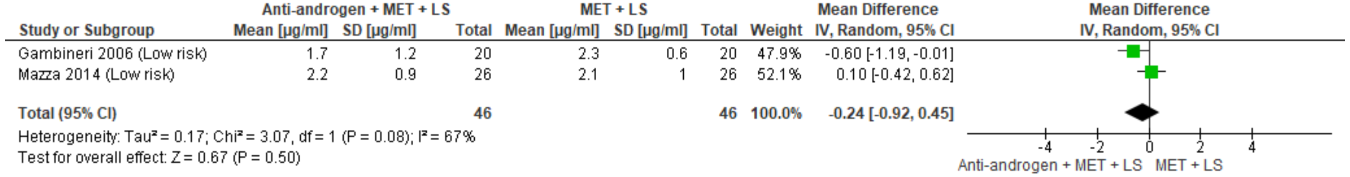

(H) Fasting insulin

Study or Subgroup	Anti-androgen + MET + LS Mean [µU/ml]	SD [µU/ml]	Total	MET + LS Mean [µU/ml]	SD [µU/ml]	Total	Weight	Mean Difference IV, Random, 95% CI
Gambineri 2006 (Low risk)	8	4	20	10	5	20	34.5%	-2.00 [-4.81, 0.81]
Ganie 2013 (Low risk)	8.9	6.35	62	10.51	7.22	56	44.7%	-1.61 [-4.07, 0.85]
Mazza 2014 (Low risk)	15.3	7.6	26	14.4	5.5	26	20.9%	0.90 [-2.71, 4.51]
**Total (95% CI)**			**108**			**102**	**100.0%**	**-1.22 [-2.87, 0.43]**

Heterogeneity: Tau² = 0.00; Chi² = 1.72, df = 2 (P = 0.42); I² = 0%
Test for overall effect: Z = 1.45 (P = 0.15)

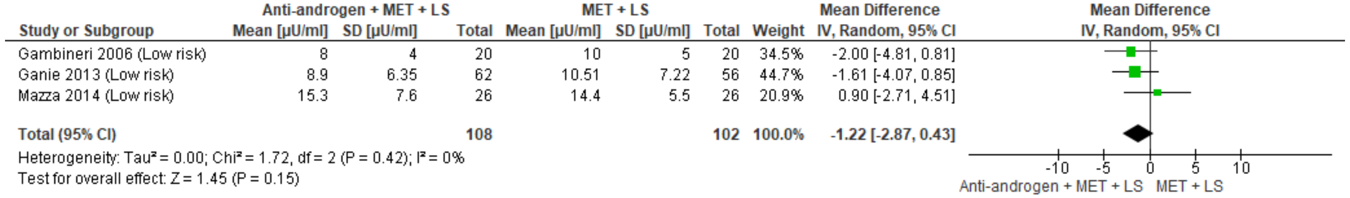

(I) Fasting glucose

Study or Subgroup	Anti-androgen + MET + LS Mean [mg/dl]	SD [mg/dl]	Total	MET + LS Mean [mg/dl]	SD [mg/dl]	Total	Weight	Mean Difference IV, Random, 95% CI
Amiri 2014 (High risk)	79.26	8.3	27	81.9	8.1	25	25.7%	-2.64 [-7.10, 1.82]
Gambineri 2006 (Low risk)	83	9	20	91	9	20	19.0%	-8.00 [-13.58, -2.42]
Ganie 2013 (Low risk)	86.14	11.79	62	88.44	11.83	56	27.1%	-2.30 [-6.57, 1.97]
Mazza 2014 (Low risk)	83.9	8.8	26	84.3	6.2	26	28.2%	-0.40 [-4.54, 3.74]
**Total (95% CI)**			**135**			**127**	**100.0%**	**-2.93 [-5.78, -0.09]**

Heterogeneity: Tau² = 3.03; Chi² = 4.69, df = 3 (P = 0.20); I² = 36%
Test for overall effect: Z = 2.02 (P = 0.04)

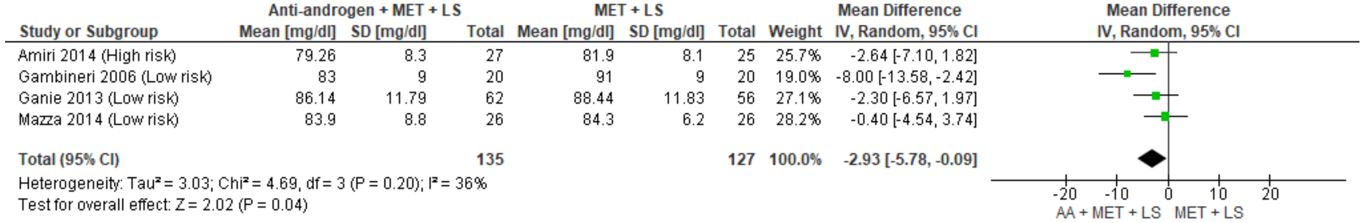

Supplementary File 2

(J) HDL

Study or Subgroup	Anti-androgen + MET + LS Mean [mg/dl]	SD [mg/dl]	Total	MET + LS Mean [mg/dl]	SD [mg/dl]	Total	Weight	Mean Difference IV, Random, 95% CI
Gambineri 2006 (Low risk)	57	17	20	50	10	20	45.9%	7.00 [-1.64, 15.64]
Mazza 2014 (Low risk)	49.8	14.2	26	52.5	10.2	26	54.1%	-2.70 [-9.42, 4.02]
**Total (95% CI)**			**46**			**46**	**100.0%**	**1.75 [-7.72, 11.23]**

Heterogeneity: Tau² = 31.44; Chi² = 3.02, df = 1 (P = 0.08); I² = 67%
Test for overall effect: Z = 0.36 (P = 0.72)

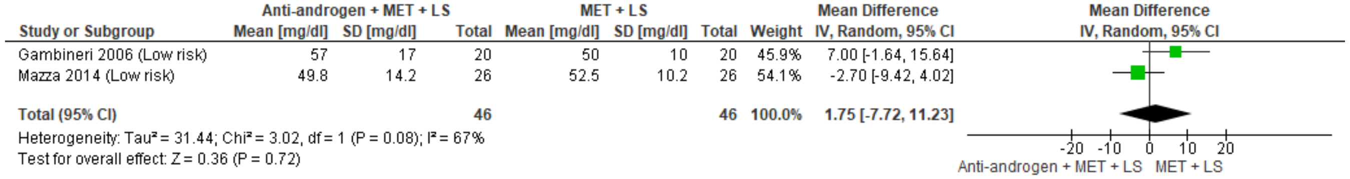

(K) LDL

Study or Subgroup	Anti-androgen + MET + LS Mean [mg/dl]	SD [mg/dl]	Total	MET + LS Mean [mg/dl]	SD [mg/dl]	Total	Weight	Mean Difference IV, Random, 95% CI
Gambineri 2006 (Low risk)	91	48	20	99	37	20	20.7%	-8.00 [-34.56, 18.56]
Mazza 2014 (Low risk)	99.8	25.2	26	108.1	24.8	26	79.3%	-8.30 [-21.89, 5.29]
**Total (95% CI)**			**46**			**46**	**100.0%**	**-8.24 [-20.34, 3.86]**

Heterogeneity: Tau² = 0.00; Chi² = 0.00, df = 1 (P = 0.98); I² = 0%
Test for overall effect: Z = 1.33 (P = 0.18)

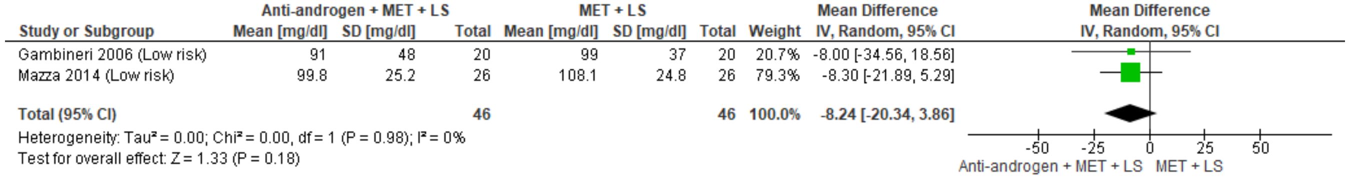

(L) Triglycerides

Study or Subgroup	Anti-androgen + MET + LS Mean [mg/dl]	SD [mg/dl]	Total	MET + LS Mean [mg/dl]	SD [mg/dl]	Total	Weight	Mean Difference IV, Random, 95% CI
Gambineri 2006 (Low risk)	82	39	20	83	52	20	42.5%	-1.00 [-29.49, 27.49]
Mazza 2014 (Low risk)	90.8	43.3	26	99.6	46.8	26	57.5%	-8.80 [-33.31, 15.71]
**Total (95% CI)**			**46**			**46**	**100.0%**	**-5.48 [-24.06, 13.10]**

Heterogeneity: Tau² = 0.00; Chi² = 0.17, df = 1 (P = 0.68); I² = 0%
Test for overall effect: Z = 0.58 (P = 0.56)

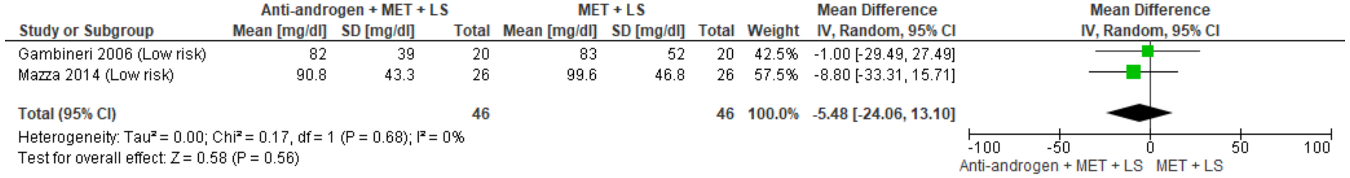

(M) HOMA-IR

Study or Subgroup	Anti-androgen + MET + LS Mean	SD	Total	MET + LS Mean	SD	Total	Weight	Mean Difference IV, Random, 95% CI
Ganie 2013 (Low risk)	1.96	1.47	62	2.31	1.5	56	71.2%	-0.35 [-0.89, 0.19]
Mazza 2014 (Low risk)	3	1.9	26	2.9	1.1	26	28.8%	0.10 [-0.74, 0.94]
**Total (95% CI)**			**88**			**82**	**100.0%**	**-0.22 [-0.67, 0.23]**

Heterogeneity: Tau² = 0.00; Chi² = 0.78, df = 1 (P = 0.38); I² = 0%
Test for overall effect: Z = 0.95 (P = 0.34)

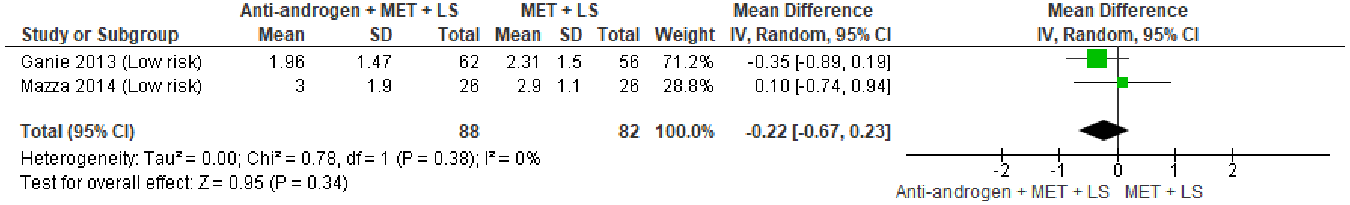

Supplementary File 2

Supplementary Table S6: Results of meta-analyses for anti-androgens + lifestyle vs anti-androgens + metformin + lifestyle - adults

Outcome	Time-point	RCTs	N	Statistical method	Effect size	P value	Favours	Certainty
BMI	6-12 months	3	204	MD (IV, random, 95% CI)	-0.05 kg/m^2 [-1.21, 1.11]	0.94	None	⊕⊕◯◯ LOW
Hirsutism	6-12 months	3	204	MD (IV, random, 95% CI)	-0.84 [-2.71, 1.03]	0.38	None	⊕◯◯◯ VERY LOW
SHBG	6-12 months	2	91	MD (IV, random, 95% CI)	8.88 nmol/l [-7.01, 24.78]	0.27	None	⊕◯◯◯ VERY LOW
Testosterone	6-12 months	3	204	MD (IV, random, 95% CI)	0.05 nmol/l [-0.15, 0.24]	0.64	None	⊕⊕◯◯ LOW
Fasting insulin	6-12 months	2	150	MD (IV, random, 95% CI)	-0.18 µIU/ml [-1.94, 1.59]	0.84	None	⊕⊕⊕⊕ HIGH
Fasting glucose	6 months	3	204	MD (IV, random, 95% CI)	3.81 mg/dl [1.35, 6.28]	**0.002**	AA + LS	⊕⊕◯◯ LOW
QUICKI	6-12 months	2	150	MD (IV, random, 95% CI)	0.00 [-0.03, 0.03]	0.79	None	⊕⊕◯◯ LOW
Weight	6 months	1	113	MD (IV, random, 95% CI)	1.11 kg [-1.97, 4.19]	0.48	None	⊕⊕⊕◯ MODERATE
WHR	6 months	1	113	MD (IV, random, 95% CI)	0.05 [0.03, 0.07]	**<0.0001**	AA + LS	⊕⊕⊕⊕ HIGH
Total cholesterol	6 months	1	54	MD (IV, random, 95% CI)	-2.24 mg/dl [-26.00, 21.52]	0.85	None	⊕◯◯◯ VERY LOW
FAI	12 months	1	37	MD (IV, random, 95% CI)	0.50 [-0.57, 1.57]	0.36	None	⊕⊕◯◯ LOW
DHEAS	12 months	1	37	MD (IV, random, 95% CI)	-0.20 µg/ml [-0.82, 0.42]	0.53	None	⊕⊕◯◯ LOW
Androstenedione	12 months	1	37	MD (IV, random, 95% CI)	-34.00 ng/ml [-98.19, 30.19]	0.30	None	⊕⊕◯◯ LOW
Triglycerides	12 months	1	37	MD (IV, random, 95% CI)	-19.00 mg/dl [-38.11, 0.11]	0.05	AA + LS	⊕⊕◯◯ LOW
HDL	12 months	1	37	MD (IV, random, 95% CI)	1.00 mg/dl [-7.59, 9.59]	0.82	None	⊕⊕◯◯ LOW
HOMA-IR	6 months	1	113	MD (IV, random, 95% CI)	0.60 [-0.04, 1.24]	0.06	None	⊕⊕⊕⊕ HIGH

AA, anti-androgen; **BMI**, body mass index; **CI**, confidence interval; **DHEAS**, dehydroepiandrosterone sulphate; **FAI**, free androgen index; **HDL**, high-density lipoprotein; **HOMA-IR**, homeostatic model assessment of insulin resistance; **IV**, inverse variance; **LS**, lifestyle; **MD**, mean difference; **OGTT**, oral glucose tolerance test; **QUICKI**, quantitative insulin sensitivity check index; **SHBG**, sex-hormone binding globulin; **WHR**, waist-hip ratio.

Supplementary File 2

Supplementary Figure S5: Forest plots for meta-analysis for pooled outcomes of BMI (A) hirsutism (B), SHBG (C), testosterone (D), fasting insulin (E), fasting glucose (F), QUICKI (G) for anti-androgens + lifestyle vs anti-androgens + metformin + lifestyle – adults.

(A) BMI

	Anti-androgen + LS			Anti-androgen + MET + LS				Mean Difference
Study or Subgroup	Mean [kg/m^2]	SD [kg/m^2]	Total	Mean [kg/m^2]	SD [kg/m^2]	Total	Weight	IV, Random, 95% CI
Amiri 2014 (High risk)	29.57	4	27	29.3	2.6	27	30.5%	0.27 [-1.53, 2.07]
Gambineri 2006 (Low risk)	29	3	17	31	5	20	16.8%	-2.00 [-4.61, 0.61]
Ganie 2013 (Low risk)	24.46	3.01	51	24.07	3.36	62	52.7%	0.39 [-0.79, 1.57]
**Total (95% CI)**			**95**			**109**	**100.0%**	**-0.05 [-1.21, 1.11]**

Heterogeneity: Tau² = 0.30; Chi² = 2.74, df = 2 (P = 0.25); I² = 27%
Test for overall effect: Z = 0.08 (P = 0.94)

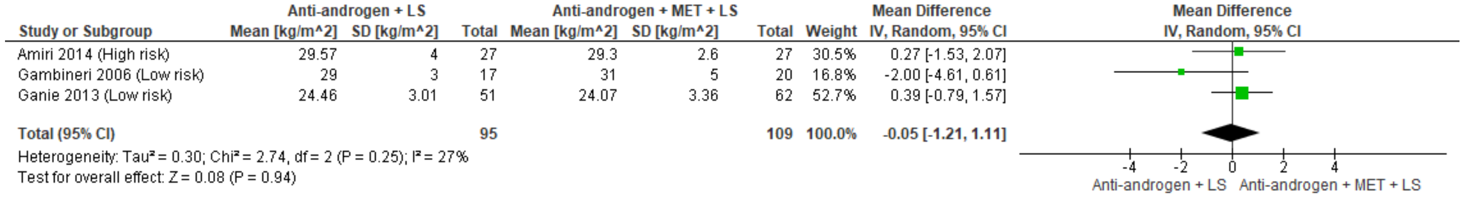

(B) Hirsutism

	Anti-androgen + LS			Anti-androgen + MET + LS				Mean Difference
Study or Subgroup	Mean [FG score]	SD [FG score]	Total	Mean [FG score]	SD [FG score]	Total	Weight	IV, Random, 95% CI
Amiri 2014 (High risk)	5	2.5	27	7.52	3.8	27	31.3%	-2.52 [-4.24, -0.80]
Gambineri 2006 (Low risk)	5.7	1.7	17	6.5	3.9	20	29.6%	-0.80 [-2.69, 1.09]
Ganie 2013 (Low risk)	9.56	2.29	51	9.09	2.29	62	39.1%	0.47 [-0.38, 1.32]
**Total (95% CI)**			**95**			**109**	**100.0%**	**-0.84 [-2.71, 1.03]**

Heterogeneity: Tau² = 2.14; Chi² = 9.80, df = 2 (P = 0.007); I² = 80%
Test for overall effect: Z = 0.88 (P = 0.38)

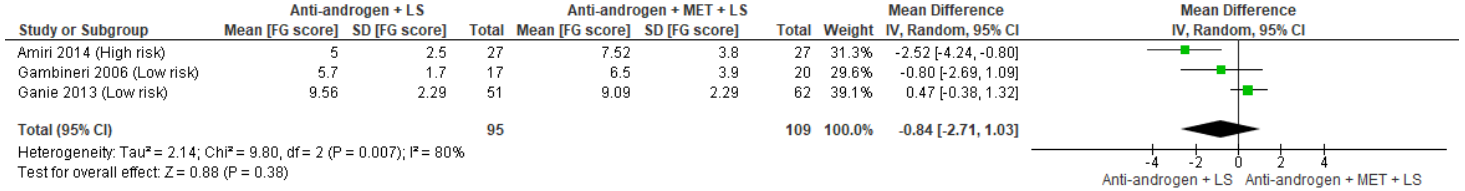

(C) SHBG

	Anti-androgen + LS			Anti-androgen + MET + LS				Mean Difference
Study or Subgroup	Mean [nmol/l]	SD [nmol/l]	Total	Mean [nmol/l]	SD [nmol/l]	Total	Weight	IV, Random, 95% CI
Amiri 2014 (High risk)	41.08	39	27	22.64	9.7	27	41.9%	18.44 [3.28, 33.60]
Gambineri 2006 (Low risk)	28.4	11.3	17	26.4	12.9	20	58.1%	2.00 [-5.80, 9.80]
**Total (95% CI)**			**44**			**47**	**100.0%**	**8.88 [-7.01, 24.78]**

Heterogeneity: Tau² = 97.31; Chi² = 3.57, df = 1 (P = 0.06); I² = 72%
Test for overall effect: Z = 1.10 (P = 0.27)

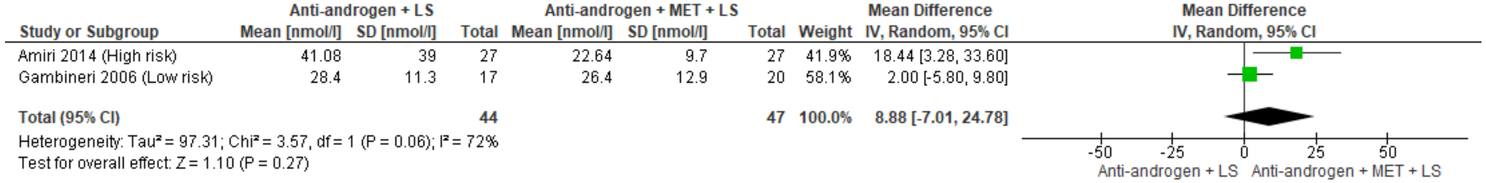

(D) Testosterone

	Anti-androgen + LS			Anti-androgen + MET + LS				Mean Difference
Study or Subgroup	Mean [nmol/l]	SD [nmol/l]	Total	Mean [nmol/l]	SD [nmol/l]	Total	Weight	IV, Random, 95% CI
Amiri 2014 (High risk)	0.55	0.2	27	0.6	0.06	27	63.6%	-0.05 [-0.13, 0.03]
Gambineri 2006 (Low risk)	1.7	0.6	17	1.5	0.7	20	16.0%	0.20 [-0.22, 0.62]
Ganie 2013 (Low risk)	1.8	1.11	51	1.58	0.74	62	20.4%	0.22 [-0.14, 0.58]
**Total (95% CI)**			**95**			**109**	**100.0%**	**0.05 [-0.15, 0.24]**

Heterogeneity: Tau² = 0.01; Chi² = 3.30, df = 2 (P = 0.19); I² = 39%
Test for overall effect: Z = 0.47 (P = 0.64)

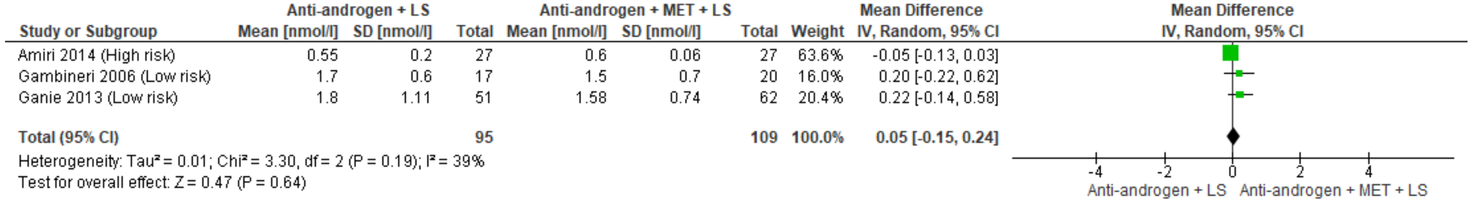

(E) Fasting insulin

	Anti-androgen + LS			Anti-androgen + MET + LS				Mean Difference
Study or Subgroup	Mean [µIU/ml]	SD [µIU/ml]	Total	Mean [µIU/ml]	SD [µIU/ml]	Total	Weight	IV, Random, 95% CI
Gambineri 2006 (Low risk)	7	5	17	8	4	20	35.6%	-1.00 [-3.95, 1.95]
Ganie 2013 (Low risk)	9.18	5.56	51	8.9	6.35	62	64.4%	0.28 [-1.92, 2.48]
**Total (95% CI)**			**68**			**82**	**100.0%**	**-0.18 [-1.94, 1.59]**

Heterogeneity: Tau² = 0.00; Chi² = 0.46, df = 1 (P = 0.50); I² = 0%
Test for overall effect: Z = 0.20 (P = 0.84)

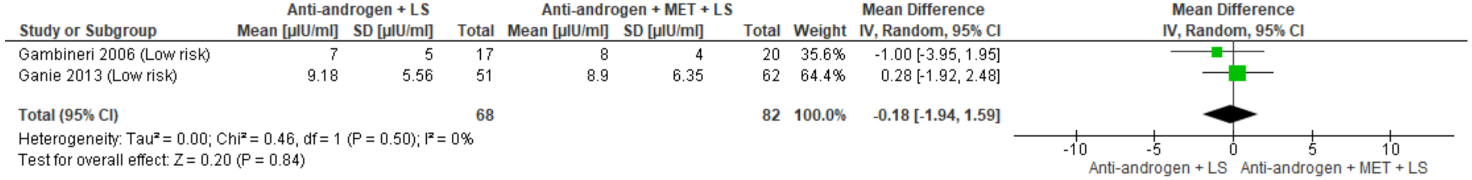

Supplementary File 2

(F) Fasting glucose

Study or Subgroup	Anti-androgen + LS Mean [mg/dl]	SD [mg/dl]	Total	Anti-androgen + MET + LS Mean [mg/dl]	SD [mg/dl]	Total	Weight	Mean Difference IV, Random, 95% CI
Amiri 2014 (High risk)	83.7	7.2	27	79.26	8.3	27	35.3%	4.44 [0.30, 8.58]
Gambineri 2006 (Low risk)	88	7	17	83	9	20	22.8%	5.00 [-0.16, 10.16]
Ganie 2013 (Low risk)	88.78	8.84	51	86.14	11.79	62	41.9%	2.64 [-1.17, 6.45]
**Total (95% CI)**			**95**			**109**	**100.0%**	**3.81 [1.35, 6.28]**

Heterogeneity: Tau² = 0.00; Chi² = 0.66, df = 2 (P = 0.72); I² = 0%
Test for overall effect: Z = 3.03 (P = 0.002)

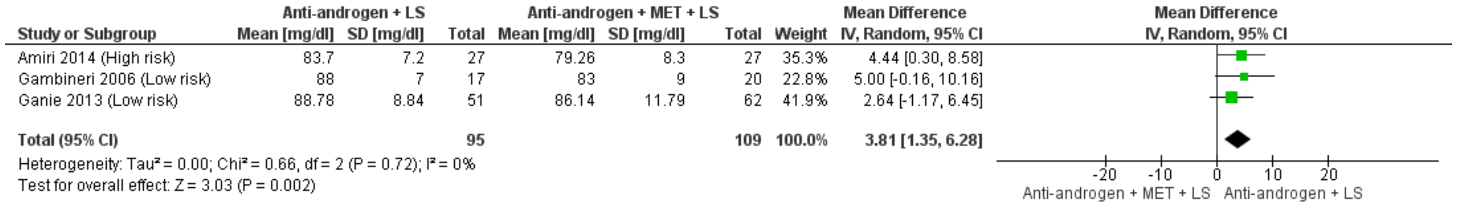

(G) QUICKI

Study or Subgroup	Anti-androgen + LS Mean	SD	Total	Anti-androgen + MET + LS Mean	SD	Total	Weight	Mean Difference IV, Random, 95% CI
Gambineri 2006 (Low risk)	0.38	0.03	17	0.36	0.03	20	46.3%	0.02 [0.00, 0.04]
Ganie 2013 (Low risk)	0.35	0.03	51	0.36	0.03	62	53.7%	-0.01 [-0.02, 0.00]
**Total (95% CI)**			**68**			**82**	**100.0%**	**0.00 [-0.03, 0.03]**

Heterogeneity: Tau² = 0.00; Chi² = 6.92, df = 1 (P = 0.009); I² = 86%
Test for overall effect: Z = 0.26 (P = 0.79)

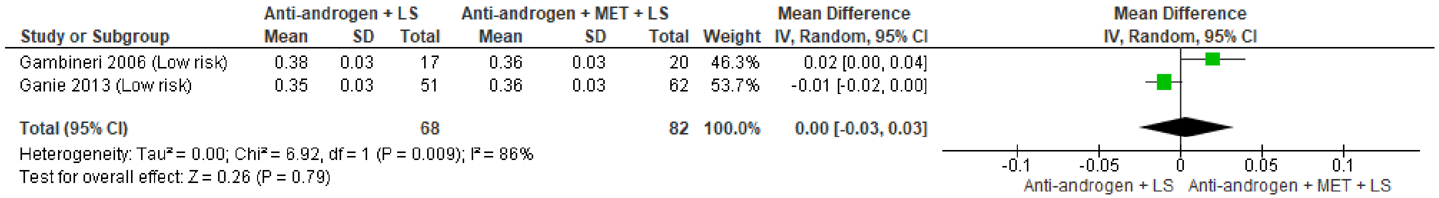

Supplementary File 2

Supplementary Table S7: Results of meta-analyses for anti-androgens + COCP vs COCP +/- placebo - adults

Outcome	Time-point	RCTs	N	Statistical method	Effect size	P value	Favours	Certainty
Weight	12 months	2	93	MD (IV, random, 95% CI)	5.57 kg [-0.67, 11.81]	0.08	None	⊕◯◯◯ VERY LOW
BMI	6-12 months	3	130	MD (IV, random, 95% CI)	1.72 kg/m^2 [-0.29, 3.74]	0.09	None	⊕⊕⊕◯ MODERATE
Testosterone	6-12 months	2	78	MD (IV, random, 95% CI)	0.10 nmol/l [-0.60, 0.79]	0.79	None	⊕◯◯◯ VERY LOW
SHBG	6-12 months	2	59	MD (IV, random, 95% CI)	-38.37 nmol/l [-118.45, 41.72]	0.35	None	⊕◯◯◯ VERY LOW
Total cholesterol	12 months	2	93	MD (IV, random, 95% CI)	20.81 mg/dl [7.81, 33.82]	**0.002**	AA + COCP	⊕⊕◯◯ LOW
HDL	12 months	2	93	MD (IV, random, 95% CI)	1.44 mg/dl [-5.55, 8.42]	0.69	None	⊕⊕◯◯ LOW
LDL	12 months	2	93	MD (IV, random, 95% CI)	15.12 mg/dl [3.20, 27.04]	**0.01**	AA + COCP	⊕⊕◯◯ LOW
Triglycerides	6-12 months	2	93	MD (IV, random, 95% CI)	41.34 mg/dl [20.26, 62.42]	**0.0001**	AA + COCP	⊕⊕◯◯ LOW
Breast tenderness	12 months	2	149	OR (IV, random, 95% CI)	0.73 [0.11, 5.00]	0.75	None	⊕◯◯◯ VERY LOW
Nausea	12 months	2	149	OR (IV, random, 95% CI)	OR: 0.67 [0.12, 3.63]	0.64	None	⊕◯◯◯ VERY LOW
ALT	12 months	1	52	MD (IV, random, 95% CI)	1.10 [-2.84, 5.04]	0.58	None	⊕⊕◯◯ LOW
AST	12 months	1	52	MD (IV, random, 95% CI)	-0.60 [-3.27, 2.07]	0.66	None	⊕⊕◯◯ LOW
Hirsutism	12 months	1	97	N/A	AA + OCP: mean: -57%, SD: 2.4%; OCP: mean: -68%, SD: 5.2%	NS	None	⊕⊕◯◯ LOW
Acne	12 months	1	97	N/A	AA + OCP: mean: -78% SD: 13.6%; OCP: -68%, SD: 26%	NS	None	⊕⊕◯◯ LOW
Fasting insulin	12 months	1	41	MD (IV, random, 95% CI)	3.50 µIU/ml [0.20, 6.80]	**0.04**	AA + COCP	⊕⊕◯◯ LOW
Fasting glucose	12 months	1	52	MD (IV, random, 95% CI)	2.60 [-1.74, 6.94]	0.24	None	⊕⊕◯◯ LOW
HOMA-IR	12 months	1	41	MD (IV, random, 95% CI)	0.70 [0.02, 1.38]	**0.04**	AA + COCP	⊕⊕◯◯ LOW
Free testosterone	6 months	1	18	MD (IV, random, 95% CI)	-0.10 pg/ml [-0.47, 0.27]	0.60	None	⊕◯◯◯ VERY LOW
FAI	12 months	1	41	MD (IV, random, 95% CI)	0.50 [0.01, 0.99]	**0.04**	AA + COCP	⊕⊕◯◯ LOW
DHEAS	6 months	1	18	MD (IV, random, 95% CI)	-0.40 µg/ml [-1.23, 0.43]	0.34	None	⊕◯◯◯ VERY LOW
Androstenedione	6 months	1	18	MD (IV, random, 95% CI)	0.10 ng/ml [-0.92, 1.12]	0.85	None	⊕◯◯◯ VERY LOW
LDL/HDL ratio	12 months	1	97	N/A	AA + OCP: Mean: -5.1%, SD: 71.3%; OCP: -5.8%, SD: 35.5%	NS	None	⊕⊕◯◯ LOW

Supplementary File 2

CRP	12 months	1	41	MD (IV, random, 95% CI)	2.40 mg/l [-1.79, 6.59]	0.26	None	⊕⊕◯◯ LOW
Menorrhagia	12 months	1	52	OR (IV, random, 95% CI)	2.67 [0.10, 68.70]	0.55	None	⊕⊕◯◯ LOW
Premenstrual pelvic pain	12 months	1	97	N/A	AA + OCP: N events: 0, total: 72; OCP + P: N events: 0, total: 25	NS	None	⊕⊕◯◯ LOW
Hyper-cholesterolaemia	12 months	1	52	OR (IV, random, 95% CI)	1.40 [0.47, 4.20]	0.55	None	⊕⊕◯◯ LOW
Hyper-triglyceridaemia	12 months	1	52	OR (IV, random, 95% CI)	2.39 [0.42, 13.64]	0.33	None	⊕⊕◯◯ LOW
Dysmenorrhea	12 months	1	52	OR (IV, random, 95% CI)	0.27 [0.01, 7.07]	0.44	None	⊕⊕◯◯ LOW
Menstrual spotting	12 months	1	52	OR (IV, random, 95% CI)	1.67 [0.42, 6.58]	0.47	None	⊕⊕◯◯ LOW
Minor depressive state/ mood reduction	12 months	1	52	OR (IV, random, 95% CI)	2.67 [0.10, 68.70]	0.55	None	⊕⊕◯◯ LOW

AA, anti-androgen; **ALT**, alanine transaminase; **AST**, aspartate transferase; **BMI**, body mass index; **CI**, confidence interval; **COCP**, combined oral contraceptive pill; **CRP**, C-reactive protein; **DHEAS**, dehydroepiandrosterone sulphate; **FAI**, free androgen index; **HDL**, high-density lipoprotein; **HOMA-IR**, homeostatic model assessment of insulin resistance; **IV**, inverse variance; **LS**, lifestyle; **MD**, mean difference; **OR**, odds ratio; **P**, placebo; **SHBG**, sex-hormone binding globulin; **WHR**, waist-hip ratio.

Supplementary File 2

Supplementary Figure S6: Forest plots for meta-analysis for pooled outcomes of weight (A), BMI (B), testosterone (C), SHBG (D), total cholesterol (E), HDL (F), LDL (G), triglycerides (H), breast tenderness (I) and nausea (J), for anti-androgens + COCP vs COCP +/- placebo – adults.

(A) Weight

	Anti-androgen + COCP			COCP +/- placebo				Mean Difference
Study or Subgroup	Mean [kg]	SD [kg]	Total	Mean [kg]	SD [kg]	Total	Weight	IV, Random, 95% CI
Moretti 2018 (Moderate risk)	71.1	17.64	28	66.3	16.56	24	45.0%	4.80 [-4.51, 14.11]
Vieira 2012 (Moderate risk)	68.2	16.7	20	62	9.7	21	55.0%	6.20 [-2.21, 14.61]
**Total (95% CI)**			**48**			**45**	**100.0%**	**5.57 [-0.67, 11.81]**

Heterogeneity: Tau² = 0.00; Chi² = 0.05, df = 1 (P = 0.83); I² = 0%
Test for overall effect: Z = 1.75 (P = 0.08)

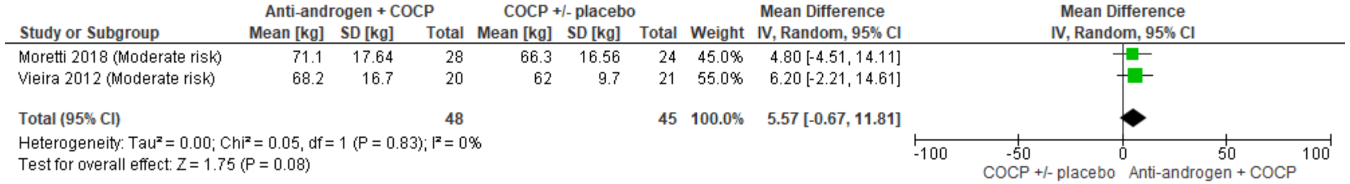

(B) BMI

	Anti-androgen + COCP			COCP +/- placebo				Mean Difference
Study or Subgroup	Mean [kg/m^2]	SD [kg/m^2]	Total	Mean [kg/m^2]	SD [kg/m^2]	Total	Weight	IV, Random, 95% CI
Burchall 2017 (Moderate risk)	35.61	5.39	16	35.99	7.96	21	21.9%	-0.38 [-4.69, 3.93]
Moretti 2018 (Moderate risk)	26.7	6.8	28	24.5	5.8	24	34.6%	2.20 [-1.22, 5.62]
Vieira 2012 (Moderate risk)	26.2	5.7	20	23.8	4.1	21	43.6%	2.40 [-0.65, 5.45]
**Total (95% CI)**			**64**			**66**	**100.0%**	**1.72 [-0.29, 3.74]**

Heterogeneity: Tau² = 0.00; Chi² = 1.18, df = 2 (P = 0.55); I² = 0%
Test for overall effect: Z = 1.68 (P = 0.09)

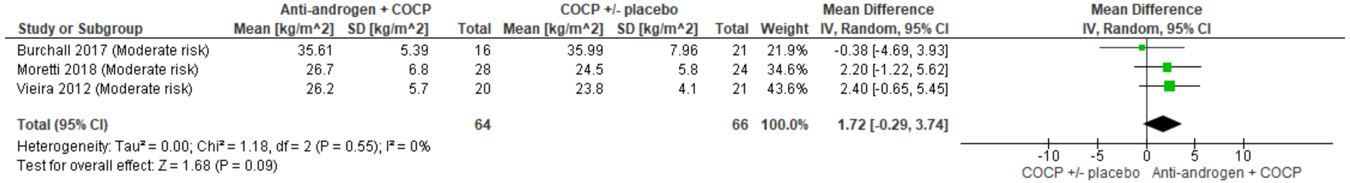

(C) Testosterone

	Anti-androgen + COCP			COCP +/- placebo				Mean Difference
Study or Subgroup	Mean [nmol/l]	SD [nmol/l]	Total	Mean [nmol/l]	SD [nmol/l]	Total	Weight	IV, Random, 95% CI
Burchall 2017 (Moderate risk)	2.11	1.21	16	1.6	0.62	21	42.4%	0.51 [-0.14, 1.16]
Vieira 2012 (Moderate risk)	1.27	0.58	20	1.48	0.55	21	57.6%	-0.21 [-0.56, 0.14]
**Total (95% CI)**			**36**			**42**	**100.0%**	**0.10 [-0.60, 0.79]**

Heterogeneity: Tau² = 0.19; Chi² = 3.68, df = 1 (P = 0.06); I² = 73%
Test for overall effect: Z = 0.27 (P = 0.79)

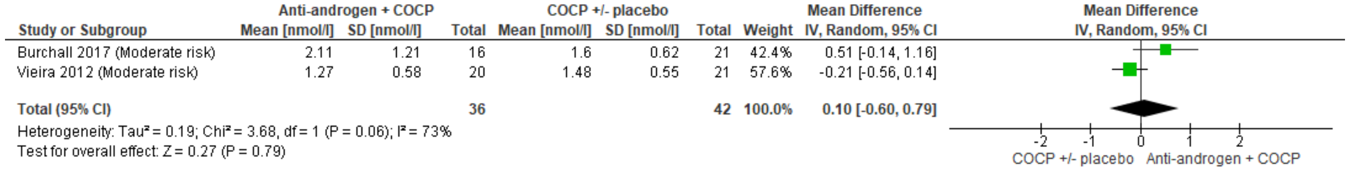

(D) SHBG

	Anti-androgen + COCP			COCP +/- placebo				Mean Difference
Study or Subgroup	Mean [nmol/l]	SD [nmol/l]	Total	Mean [nmol/l]	SD [nmol/l]	Total	Weight	IV, Random, 95% CI
Tartagni 2000 (Moderate risk)	98.95	22.1	9	101.05	10.53	9	55.9%	-2.10 [-18.09, 13.89]
Vieira 2012 (Moderate risk)	140.5	82.4	20	224.9	105.6	21	44.1%	-84.40 [-142.23, -26.57]
**Total (95% CI)**			**29**			**30**	**100.0%**	**-38.37 [-118.45, 41.72]**

Heterogeneity: Tau² = 2918.10; Chi² = 7.23, df = 1 (P = 0.007); I² = 86%
Test for overall effect: Z = 0.94 (P = 0.35)

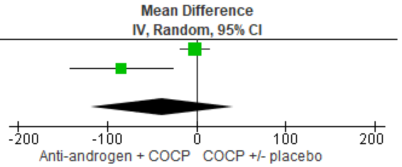

(E) Total cholesterol

	Anti-androgen + COCP			COCP +/- placebo				Mean Difference
Study or Subgroup	Mean [mg/dl]	SD [mg/dl]	Total	Mean [mg/dl]	SD [mg/dl]	Total	Weight	IV, Random, 95% CI
Moretti 2018 (Moderate risk)	206.3	30.7	28	188.7	33.5	24	54.7%	17.60 [0.02, 35.18]
Vieira 2012 (Moderate risk)	214.7	29.8	20	190	33.3	21	45.3%	24.70 [5.38, 44.02]
**Total (95% CI)**			**48**			**45**	**100.0%**	**20.81 [7.81, 33.82]**

Heterogeneity: Tau² = 0.00; Chi² = 0.28, df = 1 (P = 0.59); I² = 0%
Test for overall effect: Z = 3.14 (P = 0.002)

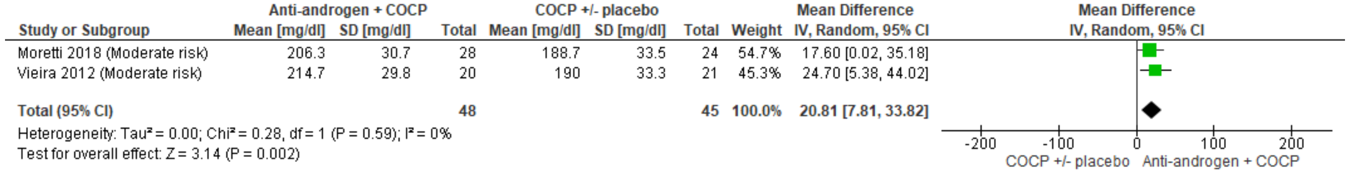

(F) HDL

Supplementary File 2

Study or Subgroup	Anti-androgen + COCP Mean [mg/dl]	SD [mg/dl]	Total	COCP +/- placebo Mean [mg/dl]	SD [mg/dl]	Total	Weight	Mean Difference IV, Random, 95% CI
Moretti 2018 (Moderate risk)	71.1	12	28	74.1	20.4	24	39.2%	-3.00 [-12.29, 6.29]
Vieira 2012 (Moderate risk)	63.4	11.4	20	59.1	9.7	21	60.8%	4.30 [-2.19, 10.79]
**Total (95% CI)**			**48**			**45**	**100.0%**	**1.44 [-5.55, 8.42]**

Heterogeneity: Tau² = 9.91; Chi² = 1.59, df = 1 (P = 0.21); I² = 37%
Test for overall effect: Z = 0.40 (P = 0.69)

Mean Difference
IV, Random, 95% CI

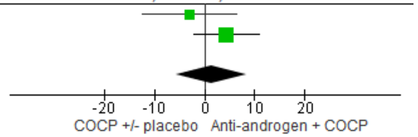

(G) LDL

Study or Subgroup	Anti-androgen + COCP Mean [mg/dl]	SD [mg/dl]	Total	COCP +/- placebo Mean [mg/dl]	SD [mg/dl]	Total	Weight	Mean Difference IV, Random, 95% CI
Moretti 2018 (Moderate risk)	111.6	24.9	28	97.4	35.9	24	48.8%	14.20 [-2.87, 31.27]
Vieira 2012 (Moderate risk)	120.4	27.1	20	104.4	27.3	21	51.2%	16.00 [-0.66, 32.66]
**Total (95% CI)**			**48**			**45**	**100.0%**	**15.12 [3.20, 27.04]**

Heterogeneity: Tau² = 0.00; Chi² = 0.02, df = 1 (P = 0.88); I² = 0%
Test for overall effect: Z = 2.49 (P = 0.01)

Mean Difference
IV, Random, 95% CI

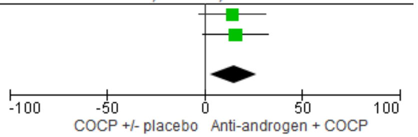

(H) Triglycerides

Study or Subgroup	Anti-androgen + COCP Mean [mg/dl]	SD [mg/dl]	Total	COCP +/- placebo Mean [mg/dl]	SD [mg/dl]	Total	Weight	Mean Difference IV, Random, 95% CI
Moretti 2018 (Moderate risk)	133.6	54.8	28	90.6	27.1	24	83.9%	43.00 [19.99, 66.01]
Vieira 2012 (Moderate risk)	165.3	100.4	20	132.6	67.1	21	16.1%	32.70 [-19.83, 85.23]
**Total (95% CI)**			**48**			**45**	**100.0%**	**41.34 [20.26, 62.42]**

Heterogeneity: Tau² = 0.00; Chi² = 0.12, df = 1 (P = 0.72); I² = 0%
Test for overall effect: Z = 3.84 (P = 0.0001)

Mean Difference
IV, Random, 95% CI

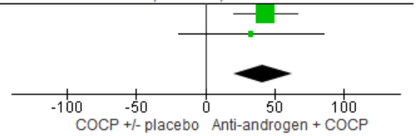

(I) Breast tenderness

Study or Subgroup	Anti-androgen + COCP Events	Total	COCP +/- placebo Events	Total	Weight	Odds Ratio IV, Random, 95% CI
Hagag 2014 (Moderate risk)	2	72	0	25	39.2%	1.81 [0.08, 38.96]
Moretti 2018 (Moderate risk)	1	28	2	24	60.8%	0.41 [0.03, 4.80]
**Total (95% CI)**		**100**		**49**	**100.0%**	**0.73 [0.11, 5.00]**
Total events	3		2			

Heterogeneity: Tau² = 0.00; Chi² = 0.55, df = 1 (P = 0.46); I² = 0%
Test for overall effect: Z = 0.32 (P = 0.75)

Odds Ratio
IV, Random, 95% CI

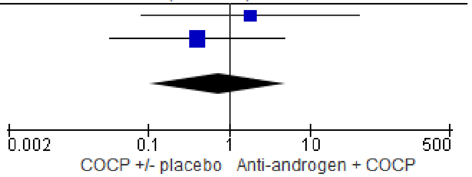

(J) Nausea

Study or Subgroup	Anti-androgen + COCP Events	Total	COCP +/- placebo Events	Total	Weight	Odds Ratio IV, Random, 95% CI
Hagag 2014 (Moderate risk)	3	72	1	25	53.2%	1.04 [0.10, 10.52]
Moretti 2018 (Moderate risk)	1	28	2	24	46.8%	0.41 [0.03, 4.80]
**Total (95% CI)**		**100**		**49**	**100.0%**	**0.67 [0.12, 3.63]**
Total events	4		3			

Heterogeneity: Tau² = 0.00; Chi² = 0.30, df = 1 (P = 0.59); I² = 0%
Test for overall effect: Z = 0.46 (P = 0.64)

Odds Ratio
IV, Random, 95% CI

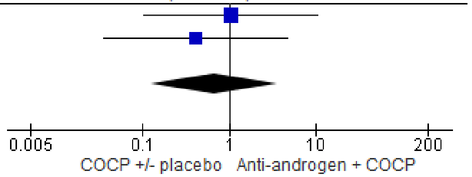

Supplementary File 2

Supplementary Table S8: Results of meta-analyses for anti-androgens + COCP vs metformin - adults

Outcome	Time-point	RCTs	N	Statistical method	Effect size	P value	Favours	Certainty
BMI	6 months	2	107	MD (IV, random, 95% CI)	-0.07 kg/m^2 [-1.81, 1.67]	0.93	None	⊕⊕⊕◯ MODERATE

Supplementary Figure S7: Forest plots for meta-analysis for pooled outcome of BMI for anti-androgens + COCP vs metformin – adults.

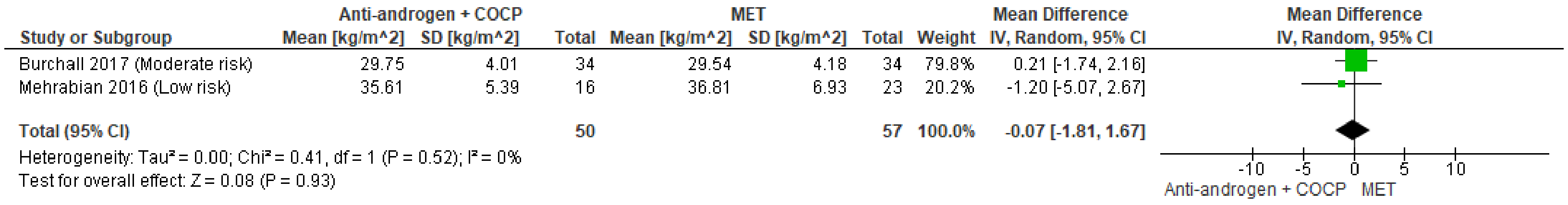

Supplementary File 3

GRADE assessment – Anti-androgen vs placebo - ADOLESCENTS

**Anti-androgen vs placebo – ADOLESCENTS (6 months)**											
	Quality assessment					No of participants					
**No. studies**	**Design**	**Risk of bias**	**Inconsistency**	**Indirectness**	**Imprecision**	**AA**	**PLAC**	**Effect, random, MD [95% CI]**	**Favours**	**Certainty**	**Importance**
Outcome: Body mass index											
1	RCT	Very serious[1]	Not applicable	Not applicable	Very Serious[2]	7	7	MD: 0.50 kg/m^2 [-3.64, 4.64]	None	⊕◯◯◯ VERY LOW	CRITICAL
Outcome: Hirsutism											
1	RCT	Very serious[1]	Not applicable	Not applicable	Very Serious[2]	7	7	MD: -4.00 [-6.81, -1.19]	Anti-androgen	⊕◯◯◯ VERY LOW	CRITICAL
Outcome: SHBG											
1	RCT	Very serious[1]	Not applicable	Not applicable	Very Serious[2]	7	7	MD: 0.00 µg/ml [-1.27, 1.27]	None	⊕◯◯◯ VERY LOW	IMPORTANT
Outcome: Testosterone											
1	RCT	Very serious[1]	Not applicable	Not applicable	Very Serious[2]	7	7	MD: 0.30 ng/dl [-2.27, 2.87]	None	⊕◯◯◯ VERY LOW	IMPORTANT
Outcome: DHEAS											
1	RCT	Very serious[1]	Not applicable	Not applicable	Very Serious[2]	7	7	MD: 0.60 µmol/l [0.07, 1.13]	Anti-androgen	⊕◯◯◯ VERY LOW	IMPORTANT
Outcome: Androstenedione											
1	RCT	Very serious[1]	Not applicable	Not applicable	Very Serious[2]	7	7	MD: -0.10 ng/ml [-1.58, 1.38]	None	⊕◯◯◯ VERY LOW	IMPORTANT

[1] Downgraded twice as the single study is high RoB
[2] Downgraded twice due to the very small sample size/participants.

Supplementary File 3

GRADE assessment – Anti-androgen vs placebo - ADULTS

Anti-androgen vs placebo – ADULTS (6 months)											
	Quality assessment					No of participants					
**No. studies**	**Design**	**Risk of bias**	**Inconsistency**	**Indirectness**	**Imprecision**	**AA**	**PLAC**	**Effect, random, MD [95% CI]**	**Favours**	**Certainty**	**Importance**
Outcome: Body weight											
1	RCT	Serious[1]	Not applicable	Not applicable	Very Serious[2]	5	6	MD: -6.50 kg [-17.11, 4.11]	None	⊕◯◯◯ VERY LOW	CRITICAL
Outcome: Body mass index											
1	RCT	Serious[1]	Not applicable	Not applicable	Very Serious[2]	5	6	MD: -0.80 kg/m^2 [-3.64, 2.04]	None	⊕◯◯◯ VERY LOW	CRITICAL
Outcome: Total Testosterone											
1	RCT	Serious[1]	Not applicable	Not applicable	Very Serious[2]	5	6	MD: 12.30 ng/dl [-22.13, 46.73]	None	⊕◯◯◯ VERY LOW	IMPORTANT
Outcome: Free Testosterone											
1	RCT	Serious[1]	Not applicable	Not applicable	Very Serious[2]	5	6	MD: 0.50 pg/ml [-2.58, 3.58]	None	⊕◯◯◯ VERY LOW	IMPORTANT
Outcome: DHEAS											
1	RCT	Serious[1]	Not applicable	Not applicable	Very Serious[2]	5	6	MD: 0.04 umol/l [-1.20, 1.27]	None	⊕◯◯◯ VERY LOW	IMPORTANT
Outcome: Androstenedione											
1	RCT	Serious[1]	Not applicable	Not applicable	Very Serious[2]	5	6	MD: 38.90 mg/dl [-84.25, 162.05]	None	⊕◯◯◯ VERY LOW	IMPORTANT
Outcome: Dihydrotestosterone											
1	RCT	Serious[1]	Not applicable	Not applicable	Very Serious[2]	5	6	MD: -0.50 ng/dl [-8.05, 7.05]	None	⊕◯◯◯ VERY LOW	IMPORTANT
Outcome: Fasting glucose											
1	RCT	Serious[1]	Not applicable	Not applicable	Very Serious[2]	5	6	MD: 0.19 mmol/l [-0.16, 0.54]	None	⊕◯◯◯ VERY LOW	IMPORTANT
Outcome: Fasting insulin											
1	RCT	Serious[1]	Not applicable	Not applicable	Very Serious[2]	5	6	MD: -3.00 uUI/ml [-6.39, 0.39]	None	⊕◯◯◯ VERY LOW	IMPORTANT
Outcome: Total cholesterol											
1	RCT	Serious[1]	Not applicable	Not applicable	Very Serious[2]	5	6	MD: 12.70 mg/dl [-15.68, 41.08]	None	⊕◯◯◯ VERY LOW	IMPORTANT

[1] Downgraded once as the single study is moderate RoB
[2] Downgraded twice due to the very small sample size/participants.

Supplementary File 3

GRADE assessment – Anti-androgen + lifestyle vs placebo + lifestyle - ADULTS

**Anti-androgen + LS vs Placebo + LS – ADULTS (6 months to 12 months)**											
	Quality assessment					No of participants					
**No. studies**	**Design**	**Risk of bias**	**Inconsistency**	**Indirectness**	**Imprecision**	**AA + LS**	**PLAC + LS**	**Effect, random**	**Favours**	**Certainty**	**Importance**
Outcome: BMI											
2[1]	RCT	No serious	Serious inconsistency[3]	Serious[6]	Serious imprecision[4]	44	45	MD: -3.08 kg/m^2 [-8.67, 2.50]	No difference	⊕◯◯◯ VERY LOW	CRITICAL
Outcome: Hirsutism											
2[1]	RCT	No serious	Serious inconsistency[3]	Serious[6]	Serious imprecision[4]	44	45	MD: -0.93 [-3.37, 1.51]	No difference	⊕◯◯◯ VERY LOW	CRITICAL
Outcome: Weight											
1[5]	RCT	No serious	Not applicable	Not applicable	Very serious imprecision[4]	17	19	MD: -17.00 kg [-25.37, -8.63]	AA + LS	⊕⊕◯◯ LOW	CRITICAL
Outcome: Frequency of menstruation											
1[5]	RCT	No serious	Not applicable	Not applicable	Very serious imprecision[4]	17	19	MD: -0.80 [-1.54, -0.06]	AA + LS	⊕⊕◯◯ LOW	CRITICAL
Outcome: SHBG											
2[1]	RCT	No serious	No serious inconsistency	Serious[6]	Serious imprecision[4]	44	45	MD: 9.72 nmol/l [-0.71, 20.14]	No difference	⊕◯◯◯ VERY LOW	IMPORTANT
Outcome: FAI											
1[5]	RCT	No serious	Not applicable	Not applicable	Very serious imprecision[4]	17	19	MD: -0.80 [-2.34, 0.74]	No difference	⊕⊕◯◯ LOW	IMPORTANT
Testosterone											
1[5]	RCT	No serious	Not applicable	Not applicable	Very serious imprecision[4]	17	19	MD: 0.05 ng/ml [-0.05, 0.15]	No difference	⊕⊕◯◯ LOW	IMPORTANT
Outcome: DHEAS											
1[5]	RCT	No serious	Not applicable	Not applicable	Very serious imprecision[4]	17	19	MD: -2.34 µg/ml [-4.06, -0.62]	AA + LS	⊕⊕◯◯ LOW	IMPORTANT
Outcome: Androstenedione											
1[5]	RCT	No serious	Not applicable	Not applicable	Very serious imprecision[4]	17	19	MD: -18.00 ng/ml [-74.86. 38.86]	No difference	⊕⊕◯◯ LOW	IMPORTANT
Outcome: Fasting insulin											
1[5]	RCT	No serious	Not applicable	Not applicable	Very serious imprecision[4]	17	19	MD: -4.00 µU/ml [-6.98, -1.02]	AA + LS	⊕⊕◯◯ LOW	IMPORTANT
Outcome: HOMA											
1[5]	RCT	No serious	Not applicable	Not applicable	Very serious imprecision[4]	17	19	MD: 1.60 [-1.64, 4.84]	No difference	⊕⊕◯◯ LOW	IMPORTANT
Outcome: Fasting glucose											
1[5]	RCT	No serious	Not applicable	Not applicable	Very serious imprecision[4]	17	19	MD: 0.00 mg/ml [-5.24, 5.24]	No difference	⊕⊕◯◯ LOW	IMPORTANT

Supplementary File 3

Outcome: QUICKI											
1[5]	RCT	No serious	Not applicable	Not applicable	Very serious imprecision[4]	17	19	MD: 0.04 [0.02, 0.06]	AA +LS	⊕⊕◯◯ LOW	IMPORTANT
Outcome: Insulin sensitivity index											
1[5]	RCT	No serious	Not applicable	Not applicable	Very serious imprecision[4]	17	19	MD: 5.10 [2.32, 7.88]	AA +LS	⊕⊕◯◯ LOW	IMPORTANT
Outcome: HDL											
1[5]	RCT	No serious	Not applicable	Not applicable	Very serious imprecision[4]	17	19	MD: 5.00 mg/dl [-1.54, 11.54]	No difference	⊕⊕◯◯ LOW	IMPORTANT
Outcome: LDL											
1[5]	RCT	No serious	Not applicable	Not applicable	Very serious imprecision[4]	17	19	MD: -21.00 mg/dl [-40.93, -1.07]	AA + LS	⊕⊕◯◯ LOW	IMPORTANT
Outcome: Triglycerides											
1[5]	RCT	No serious	Not applicable	Not applicable	Very serious imprecision[4]	17	19	MD: -50.00 mg/dl [-77.60, -22.40]	AA + LS	⊕⊕◯◯ LOW	IMPORTANT

[1] Gambineri 2006, Amiri 2014
[2] Not downgraded as evidence is either low or moderate risk, and the study with low risk has a weighting of 64.9%.
[3] Downgraded once due to statistically significant heterogeneity
[4] Downgraded once due to small sample size or twice for very small sample.
[5] Gambineri 2006 (low risk of bias study).
[6] Downgraded for indirectness as Amiri 2014 stated it was an overweight population, but the study has an inclusion of women with BMI 19 to 35 (not reporting BMIs), thus indicating this population could include lean women, whereas Gambineri 2006 is an overweight population with reported BMIs.

Supplementary File 3

GRADE assessment – Anti-androgen (every day) vs Anti-androgen (every 3 days) - ADULTS

Anti-androgen (every day) vs Anti-androgen (every 3 days) – ADULTS (6 months to 12 months)											
	Quality assessment					No of participants					
No. studies	Design	Risk of bias	Inconsistency	Indirectness	Imprecision	Daily AA	Every 3d AA	Effect, random, MD or OR	Favours	Certainty	Importance
Outcome: Hirsutism											
2	RCT	Very serious[1]	No serious inconsistency	No serious	Serious imprecision[3]	40	40	MD: -3.48 [-4.58, -2.39]	Daily Anti-androgen	⊕◯◯◯ VERY LOW	CRITICAL
Outcome: Body mass index											
1	RCT	Very serious[5]	Not applicable	Not applicable	Very serious imprecision[3]	8	8	MD: 0.30 kg/m^2 [-3.67, 4.27]	No difference	⊕◯◯◯ VERY LOW	CRITICAL
Outcome: SHBG											
2	RCT	Very serious[1]	No serious inconsistency	Not applicable	Serious imprecision[3]	40	40	MD: 0.29 nmol/l [-2.18, 2.76]	No difference	⊕◯◯◯ VERY LOW	IMPORTANT
Outcome: Androstenedione											
2	RCT	Very serious[1]	No serious inconsistency	Not applicable	Very serious imprecision[3,4]	40	40	MD: -0.30 ng/ml [-0.50, -0.10]	No difference	⊕◯◯◯ VERY LOW	IMPORTANT
Outcome: Testosterone											
2	RCT	Very serious[1]	Very serious incosistency[2]	Not applicable	Serious imprecision[3]	40	40	MD: -0.25 ng/ml [-0.73, 0.23]	No difference	⊕◯◯◯ VERY LOW	IMPORTANT
Outcome: DHEAS											
2	RCT	Very serious[1]	Very serious incosistency[2]	Not applicable	Serious imprecision[3]	40	40	MD: 0.19 µg/ml [-0.79, 1.17]	No difference	⊕◯◯◯ VERY LOW	IMPORTANT
Outcome: Fasting insulin											
1	RCT	Very serious[5]	Not applicable	Not applicable	Serious imprecision[3]	32	32	MD: 0.70 µU/ml [-0.17, 1.57]	No difference	⊕◯◯◯ VERY LOW	IMPORTANT
Outcome: Dry skin											
1	RCT	Very serious[5]	Not applicable	Not applicable	Serious imprecision[3]	32	32	OR: 6.60 [2.21, 19.73]	Daily Anti-androgen	⊕◯◯◯ VERY LOW	IMPORTANT
Outcome: Decreased libido											
1	RCT	Very serious[5]	Not applicable	Not applicable	Serious imprecision[3]	32	32	OR: 1.62 [0.41, 6.38]	No difference	⊕◯◯◯ VERY LOW	IMPORTANT
Outcome: Headache											
1	RCT	Very serious[5]	Not applicable	Not applicable	Serious imprecision[3]	32	32	OR: 0.23 [0.02, 2.14]	No difference	⊕◯◯◯ VERY LOW	IMPORTANT

[1] Downgraded twice as both studies are high risk of bias
[2] Downgraded twice due to no overlapping CIs, and statistically significant heterogeneity
[3] Downgraded once due to small sample size or twice for very small sample size
[4] Downgraded due to indirectness because of one study
[5] Downgraded twice due to single study assessed is high RoB

Supplementary File 3

GRADE assessment – Anti-androgen vs metformin - ADULTS

Anti-androgen vs MET – ADULTS (12 months)											
	Quality assessment					No of participants					
**No. studies**	**Design**	**Risk of bias**	**Inconsistency**	**Indirectness**	**Imprecision**	**Met**	**OCP**	**Effect, fixed**	**Favours**	**Certainty**	**Importance**
Outcome: BMI											
1	RCT	Very serious[1]	Not applicable	Not applicable	Very serious[2]	16	19	MD: -0.20 kg/m^2 [-2.37, 1.97]	No difference	⊕◯◯◯ VERY LOW	CRITICAL
Outcome: Hirsutism											
1	RCT	Very serious[1]	Not applicable	Not applicable	Very serious[2]	16	19	MD: 0.60 [-2.80, 4.00]	No difference	⊕◯◯◯ VERY LOW	CRITICAL
Outcome: SHBG											
1	RCT	Very serious[1]	Not applicable	Not applicable	Very serious[2]	16	19	MD: 11.50 nmol/l [-0.08, 23.08]	Anti-androgen	⊕◯◯◯ VERY LOW	IMPORTANT
Outcome: Free testosterone											
1	RCT	Very serious[1]	Not applicable	Not applicable	Very serious[2]	16	19	MD: -0.30 pg/ml [-0.85, 0.25]	No difference	⊕◯◯◯ VERY LOW	IMPORTANT
Outcome: DHEAS											
1	RCT	Very serious[1]	Not applicable	Not applicable	Very serious[2]	16	19	MD: -669.00 ng/ml [-1430.65, 92.65]	No difference	⊕◯◯◯ VERY LOW	IMPORTANT
Outcome: Androstenedione											
1	RCT	Very serious[1]	Not applicable	Not applicable	Very serious[2]	16	19	MD: 0.30 ng/ml [-0.13, 0.73]	No difference	⊕◯◯◯ VERY LOW	IMPORTANT
Outcome: HOMA-IR											
1	RCT	Very serious[1]	Not applicable	Not applicable	Very serious[2]	16	19	MD: -0.20 [-0.88, 0.48]	No difference	⊕◯◯◯ VERY LOW	IMPORTANT

[1] Downgraded twice as only study assessed is high RoB
[2] Downgraded twice as very small sample size

Supplementary File 3

GRADE assessment – Anti-androgen vs Anti-androgen + metformin - ADULTS

**Anti-androgen vs Anti-androgen + MET – ADULTS (12 months)**											
	Quality assessment					No of participants					
**No. studies**	**Design**	**Risk of bias**	**Inconsistency**	**Indirectness**	**Imprecision**	**AA**	**AA + MET**	**Effect, fixed**	**Favours**	**Certainty**	**Importance**
Outcome: BMI											
1	RCT	Very serious[1]	Not applicable	Not applicable	Very serious[2]	16	17	MD: 0.10 kg/m^2 [-2.25, 2.45]	No difference	⊕◯◯◯ VERY LOW	CRITICAL
Outcome: Hirsutism											
1	RCT	Very serious[1]	Not applicable	Not applicable	Very serious[2]	16	17	MD: -0.40 [-4.05, 3.25]	No difference	⊕◯◯◯ VERY LOW	CRITICAL
Outcome: SHBG											
1	RCT	Very serious[1]	Not applicable	Not applicable	Very serious[2]	16	17	MD: -1.00 nmol/l [-14.72, 12.72]	No difference	⊕◯◯◯ VERY LOW	IMPORTANT
Outcome: Free testosterone											
1	RCT	Very serious[1]	Not applicable	Not applicable	Very serious[2]	16	17	MD: 0.10 pg/ml [-0.52, 0.72]	No difference	⊕◯◯◯ VERY LOW	IMPORTANT
Outcome: DHEAS											
1	RCT	Very serious[1]	Not applicable	Not applicable	Very serious[2]	16	17	MD: -198 ng/ml [-941.98, 545.98]	No difference	⊕◯◯◯ VERY LOW	IMPORTANT
Outcome: Androstenedione											
1	RCT	Very serious[1]	Not applicable	Not applicable	Very serious[2]	16	17	MD: 0.30 ng/ml [-0.14, 0.74]	No difference	⊕◯◯◯ VERY LOW	IMPORTANT
Outcome: HOMA-IR											
1	RCT	Very serious[1]	Not applicable	Not applicable	Very serious[2]	16	17	MD: -0.40 [-1.07, 0.27]	No difference	⊕◯◯◯ VERY LOW	IMPORTANT

[1] Downgraded twice as only study assessed is high RoB
[2] Downgraded twice as very small sample size

Supplementary File 3

GRADE assessment – Anti-androgen + lifestyle vs metformin + lifestyle - ADULTS

Anti-androgen + LS vs MET + LS - ADULTS (6 months to 12 months)											
	Quality assessment					No of participants					
**No. studies**	**Design**	**Risk of bias**	**Inconsistency**	**Indirectness**	**Imprecision**	**AA + LS**	**MET + LS**	**Effect, fixed**	**Favours**	**Certainty**	**Importance**
Outcome: Weight											
2	RCT	No serious	Serious[2]	No serious	Serious[5]	68	76	MD: -5.64 kg [-19.33, 8.05]	No difference	⊕⊕◯◯ LOW	CRITICAL
Outcome: BMI											
4	RCT	Serious[1]	Serious[3]	Serious[6]	No serious	129	136	MD: -0.79 kg/m^2 [-2.45, 0.87]	No difference	⊕◯◯◯ VERY LOW	CRITICAL
Outcome: Hirsutism											
4	RCT	Serious[1]	Serious[3]	Serious[6]	No serious	129	136	MD: -1.59 [-3.06, -0.12]	AA + LS	⊕◯◯◯ VERY LOW	CRITICAL
Outcome: Frequency of menstruation											
2	RCT	Serious[1]	No serious	No serious	No serious	85	91	MD: 0.79 cycles/year [0.05, 1.53]	AA + LS	⊕⊕⊕◯ MODERATE	CRITICAL
Outcome: WHR											
2	RCT	Serious[1]	No serious	No serious	No serious	85	91	MD: 0.01 [-0.04, 0.06]	No difference	⊕⊕⊕◯ MODERATE	IMPORTANT
Outcome: SHBG											
2	RCT	Serious[1]	No serious	Serious[6]	Serious[4]	44	45	MD: 7.70 nmol/l [0.75, 14.66]	AA + LS	⊕◯◯◯ VERY LOW	IMPORTANT
Outcome: Testosterone											
3	RCT	Serious[1]	No serious	No serious	No serious	102	111	MD: -0.01 nmol/l [-0.26, 0.24]	No difference	⊕⊕⊕◯ MODERATE	IMPORTANT
Outcome: DHEAS											
2	RCT	Serious[1]	Very serious[2,3]	No serious	No serious	51	55	MD: -1.02 μmol/l [-3.27, 1.24]	No difference	⊕◯◯◯ VERY LOW	IMPORTANT
Outcome: Fasting insulin											
3	RCT	Serious[1]	No serious	No serious	No serious	102	111	MD: -2.11 μU/ml [-3.97, -0.26]	AA + LS	⊕⊕⊕◯ MODERATE	IMPORTANT
Outcome: Fasting glucose											
3	RCT	Serious[1]	No serious	Serious[6]	No serious	112	116	MD: 0.44 mg/dl [-2.14, 3.02]	No difference	⊕⊕◯◯ LOW	IMPORTANT
Outcome: Fasting glucose-insulin ratio											
2	RCT	Serious[1]	No serious	No serious	No serious	85	91	MD: -1.12 [-1.44, -0.79]	AA + LS	⊕⊕⊕◯ MODERATE	IMPORTANT
Outcome: QUICKI											
2	RCT	No serious	Serious[3]	No serious	No serious	68	76	MD: 0.01 [-0.02, 0.04]	No difference	⊕⊕⊕◯ MODERATE	IMPORTANT
Outcome: FAI											

Supplementary File 3

1	RCT	No serious	Not applicable	Not applicable	Very Serious[4]	17	20	MD: -0.60 [-1.99, 0.79]	No difference	⊕⊕◯◯ LOW	IMPORTANT
Outcome: Androstenedione											
1	RCT	No serious	Not applicable	Not applicable	Very Serious[4]	17	20	MD: -39.00 ng/dl [-123.43, 45.43]	No difference	⊕⊕◯◯ LOW	IMPORTANT
Outcome: OGTT											
1	RCT	Serious[1]	Not applicable	Not applicable	Very Serious[4]	27	25	MD: -9.54 mg/dl [-23.70, 4.62]	No difference	⊕◯◯◯ VERY LOW	IMPORTANT
Outcome: HOMA											
1	RCT	Serious[1]	Not applicable	Not applicable	Serious[4]	34	35	MD: 2.72 [-0.28, 5.72]	No difference	⊕⊕◯◯ LOW	IMPORTANT
Outcome: Total cholesterol											
1	RCT	Serious[1]	Not applicable	Not applicable	Very Serious[4,5]	27	25	MD: 7.20 mmol/l [-13.06, 27.46]	No difference	⊕◯◯◯ VERY LOW	IMPORTANT
Outcome: HDL											
1	RCT	No serious	Not applicable	Not applicable	Very Serious[4]	17	20	MD: 0.21 mmol/l [0.05, 0.37]	AA + LS	⊕⊕◯◯ LOW	IMPORTANT
Outcome: LDL											
1	RCT	No serious	Not applicable	Not applicable	Very Serious[4]	17	20	MD: -0.29 mmol/l [-0.83, 0.25]	No difference	⊕⊕◯◯ LOW	IMPORTANT
Outcome: Triglycerides											
1	RCT	No serious	Not applicable	Not applicable	Very Serious[4]	17	20	MD: -0.23 mmol/l [-0.51, 0.05]	No difference	⊕⊕◯◯ LOW	IMPORTANT

[1] One or more studies have moderate RoB with significant weighting
[2] Downgraded for no overlapping CIs
[3] Downgraded for statistically significant ($p<0.05$) or high heterogeneity (>50%).
[4] Downgraded due to sample size/participants.
[5] Downgraded due to wide CIs
[6] Downgraded for indirectness as Amiri 2014 stated it was an overweight population, but the study has an inclusion of women with BMI 19 to 35 (not reporting BMIs), thus indicating this population could include lean women, whereas the other studies have clearly reported BMIs in overweight/obese individuals.

Supplementary File 3

GRADE assessment – Anti-androgen + metformin + lifestyle vs metformin + lifestyle - ADULTS

**Anti-androgen + MET + LS vs MET + LS – ADULTS (6 months to 12 months)**											
	Quality assessment					No of participants					
**No. studies**	**Design**	**Risk of bias**	**Inconsistency**	**Indirectness**	**Imprecision**	**AA + MET + LS**	**MET + LS**	**Effect, fixed**	**Favours**	**Certainty**	**Importance**
Outcome: Weight											
3	RCT	No serious	Serious[2]	No serious	Serious[5]	108	102	MD: -0.92 kg [-7.30, 5.45]	No difference	⊕⊕◯◯ LOW	CRITICAL
Outcome: BMI											
4	RCT	Serious[1]	No serious	Serious[4]	No serious	135	127	MD: -0.20 kg/m^2 [-1.43, 1.02]	No difference	⊕⊕◯◯ LOW	CRITICAL
Outcome: Hirsutism											
4	RCT	Serious[1]	No serious	Serious[4]	No serious	135	127	MD: -0.58 [-1.86, 0.69]	No difference	⊕⊕◯◯ LOW	CRITICAL
Frequency of menstruation (number of cycles per year)											
1	RCT	No serious	Not applicable	Not applicable	No serious	62	56	MD: -0.84 cycles/year [-0.31, 1.99]	No difference	⊕⊕⊕⊕ HIGH	CRITICAL
Outcome: WHR											
2	RCT	Serious[1]	Serious[2]	Serious[4]	No serious	89	81	MD: -0.00 [-0.06, 0.06]	No difference	⊕◯◯◯ VERY LOW	IMPORTANT
Outcome: SHBG											
3	RCT	Serious[1]	No serious	Serious[4]	No serious	73	71	MD: -0.59 nmol/l [-4.53, 3.34]	No difference	⊕⊕◯◯ LOW	IMPORTANT
Outcome: Testosterone											
4	RCT	Serious[1]	Serious[2]	Serious[4]	No serious	135	127	MD: -0.29 nmol/l [-0.52, -0.06]	AA + MET + LS	⊕◯◯◯ VERY LOW	IMPORTANT
Outcome: DHEAS											
2	RCT	No serious	Serious[2]	No serious	No serious	46	46	MD: -0.24 µg/ml [-0.92, 0.45]	No difference	⊕⊕⊕◯ MODERATE	IMPORTANT
Outcome: Fasting insulin											
3	RCT	No serious	No serious	No serious	No serious	108	102	MD: -1.22 µU/ml [-2.87, 0.43]	No difference	⊕⊕⊕⊕ HIGH	IMPORTANT
Outcome: Fasting glucose											
4	RCT	Serious[1]	No serious	Serious[4]	No serious	135	127	MD: -2.93 mg/dl [-5.78, -0.09]	AA + MET + LS	⊕⊕◯◯ LOW	IMPORTANT
Outcome: HDL											
2	RCT	No serious	Serious[2]	No serious	Serious[6]	46	46	MD: 1.75 mg/dl [-7.72, 11.23]	No difference	⊕⊕◯◯ LOW	IMPORTANT
Outcome: LDL											
2	RCT	No serious	No serious	No serious	Serious[6]	46	46	MD: -8.24 mg/dl [-20.34, 3.86]	No difference	⊕⊕⊕◯ MODERATE	IMPORTANT
Outcome: Triglycerides											

Supplementary File 3

2	RCT	No serious	No serious	No serious	Serious[6]	46	46	MD: -5.48 mg/dl [-24.06, 13.10]	No difference	⊕⊕⊕◯ MODERATE	IMPORTANT
HOMA-IR											
2	RCT	No serious	No serious	No serious	No serious	88	82	MD: -0.22 [-0.67, 0.23]	No difference	⊕⊕⊕⊕ HIGH	IMPORTANT
FAI											
1	RCT	No serious	Not applicable	Not applicable	Serious	26	26	MD: -2.10 [-4.47, 0.27]	No difference	⊕⊕⊕◯ MODERATE	IMPORTANT
Androstenedione											
1	RCT	No serious	Not applicable	Not applicable	Very Serious[6]	20	20	MD: -5.00 ng/dl [-96.42, 86.42]	No difference	⊕⊕◯◯ LOW	IMPORTANT
Fasting glucose-insulin ratio											
1	RCT	No serious	Not applicable	Not applicable	No serious	62	56	MD: 1.74 [-1.45, 4.93]	No difference	⊕⊕⊕⊕ HIGH	IMPORTANT
QUICKI											
1	RCT	No serious	Not applicable	Not applicable	Very Serious[6]	20	20	MD: 0.01 [-0.01, 0.03]	No difference	⊕⊕◯◯ LOW	IMPORTANT
OGTT											
1	RCT	Serious[1]	Not applicable	Not applicable	Serious[6]	27	25	MD: -4.88 mg/dl [-20.32, 10.56]	No difference	⊕⊕◯◯ LOW	IMPORTANT
Insulin sensitivity index											
1	RCT	No serious	Not applicable	Not applicable	Very Serious[6]	20	20	MD: 6.90 [2.96, 10.84]	AA + MET + LS	⊕⊕◯◯ LOW	IMPORTANT
Total cholesterol											
1	RCT	No serious	Not applicable	Not applicable	Serious[6]	26	26	MD: 0.30 mg/dl [-16.67, 17.36]	No difference	⊕⊕⊕◯ MODERATE	IMPORTANT

[1] One study has moderate RoB and has significant weighting
[2] Downgraded for statistically significant or high heterogeneity (>50%)
[3] Downgraded for no overlapping CIs
[4] Downgraded for indirectness as Amiri 2014 stated it was an overweight population, but the study has an inclusion of women with BMI 19 to 35 (not reporting BMIs), thus indicating this population could include lean women, whereas the other studies have clearly reported BMIs in overweight/obese individuals.
[5] Downgraded for wide CIs
[6] Downgraded once for small sample and twice for very small sample

Supplementary File 3

GRADE assessment – Anti-androgen + lifestyle vs Anti-androgen + metformin + lifestyle - ADULTS

**Anti-androgen + LS vs Anti-androgen + MET + LS – ADULTS (6 months to 12 months)**											
	Quality assessment					No of participants					
**No. studies**	**Design**	**Risk of bias**	**Inconsistency**	**Indirectness**	**Imprecision**	**AA + LS**	**AA + MET + LS**	**Effect, random**	**Favours**	**Certainty**	**Importance**
Outcome: BMI											
3	RCT	Serious[1]	No serious	Serious[7]	No serious	95	109	MD: -0.05 kg/m^2 [-1.21, 1.11]	No difference	⊕⊕◯◯ LOW	CRITICAL
Outcome: Hirsutism											
3	RCT	Serious[1]	Serious[2,3]	Serious[7]	No serious	95	109	MD: -0.84 [-2.71, 1.03]	No difference	⊕◯◯◯ VERY LOW	CRITICAL
Outcome: Weight											
1	RCT	No serious	Not applicable	Not applicable	Serious[6]	51	62	MD: 1.11 kg [-1.97, 4.19]	No difference	⊕⊕⊕◯ MODERATE	CRITICAL
Outcome: SHBG											
2	RCT	Serious[1]	Serious[3]	Serious[7]	Very serious[5,6]	44	47	MD: 8.88 nmol/l [-7.01, 24.78]	No difference	⊕◯◯◯ VERY LOW	IMPORTANT
Outcome: Testosterone											
3	RCT	Serious[1]	No serious	Serious[7]	No serious	95	109	MD: 0.05 nmol/l [-0.15, 0.24]	No difference	⊕⊕◯◯ LOW	IMPORTANT
Outcome: Fasting insulin											
2	RCT	No serious	No serious	No serious	No serious	68	82	MD: -0.18 µIU/ml [-1.94, 1.59]	No difference	⊕⊕⊕⊕ HIGH	IMPORTANT
Outcome: Fasting glucose											
3	RCT	Serious[1]	No serious	Serious[7]	No serious	95	109	MD: 3.81 mg/dl [1.35, 6.28]	AA + LS	⊕⊕◯◯ LOW	IMPORTANT
Outcome: QUICKI											
2	RCT	No serious	Very serious[2,3]	No serious	No serious	68	82	MD: 0.00 [-0.03, 0.03]	No difference	⊕⊕◯◯ LOW	IMPORTANT
Outcome: WHR											
1	RCT	No serious	Not applicable	Not applicable	No serious	51	62	MD: 0.05 [0.03, 0.07]	AA + LS	⊕⊕⊕⊕ HIGH	IMPORTANT
Outcome: Total cholesterol											
1	RCT	Serious[1]	Not applicable	Not applicable	Very serious[5,6]	27	27	MD: -2.24 mg/dl [-26.00, 21.52]	No difference	⊕◯◯◯ VERY LOW	IMPORTANT
Outcome: FAI											
1	RCT	No serious	Not applicable	Not applicable	Very Serious[5]	17	20	MD: 0.50 [-0.57, 1.57]	No difference	⊕⊕◯◯ LOW	IMPORTANT
Outcome: DHEAS											
1	RCT	No serious	Not applicable	Not applicable	Very Serious[5]	17	20	MD: -0.20 µg/ml [-0.82, 0.42]	No difference	⊕⊕◯◯ LOW	IMPORTANT
Outcome: Androstenedione											

Supplementary File 3

1	RCT	No serious	Not applicable	Not applicable	Very serious[5,6]	17	20	MD: -34.00 ng/ml [-98.19, 30.19]	No difference	⊕⊕◯◯ LOW	IMPORTANT
Outcome: Triglycerides											
1	RCT	No serious	Not applicable	Not applicable	Very serious[5,6]	17	20	MD: -19.00 mg/dl [-38.11, 0.11]	AA + LS	⊕⊕◯◯ LOW	IMPORTANT
Outcome: HDL											
1	RCT	No serious	Not applicable	Not applicable	Very serious[5,6]	17	20	MD: 1.00 mg/dl [-7.59, 9.59]	No difference	⊕⊕◯◯ LOW	IMPORTANT
Outcome: HOMA-IR											
1	RCT	No serious	Not applicable	Not applicable	No serious	51	62	MD: 0.60 [-0.04, 1.24]	No difference	⊕⊕⊕⊕ HIGH	IMPORTANT

[1] Downgraded once as Amiri 2014 is moderate risk of bias and has sufficient weighting OR only identified study is moderate RoB
[2] Downgraded once as one study or more has no overlapping CIs
[3] Downgraded once due to statistical heterogeneity
[5] Downgraded due to small sample size or very small sample size
[6] Downgraded due to wide CIs
[7] Downgraded for indirectness as Amiri 2014 stated it was an overweight population, but the study has an inclusion of women with BMI 19 to 35 (not reporting BMIs), thus indicating this population could include lean women, whereas Gambineri 2006 is an overweight population with reported BMIs.

GRADE assessment – Anti-androgen vs COCP – ADULTS

**Anti-androgen vs COCP – ADULTS (12 months)**											
	Quality assessment					No of participants					
**No. studies**	**Design**	**Risk of bias**	**Inconsistency**	**Indirectness**	**Imprecision**	**AA**	**COCP**	**Effect, fixed**	**Favours**	**Certainty**	**Importance**
Outcome: Hirsutism											
1	RCT	Serious[1]	Not applicable	Not applicable	Very serious[2]	10	9	MD: 4.00 [1.21, 6.79]	Anti-androgen	⊕◯◯◯ VERY LOW	CRITICAL

[1] Downgraded twice as only study assessed is high RoB
[2] Downgraded twice for very small sample size

Supplementary File 3

GRADE assessment – Anti-androgen + COCP vs COCP +/- Placebo – ADULTS

**Anti-androgen + COCP vs COCP +/- Placebo – ADULTS (6 months to 12 months)**											
	Quality assessment					No of participants					
**No. studies**	**Design**	**Risk of bias**	**Inconsistency**	**Indirectness**	**Imprecision**	**AA + COCP**	**COCP +/- PLAC**	**Effect, fixed**	**Favours**	**Certainty**	**Importance**
Outcome: Weight											
2	RCT	Serious[1]	No serious	No serious	Very Serious[5,6]	48	45	MD: 5.57 kg [-0.67, 11.85]	No difference	⊕◯◯◯<br>VERY LOW	CRITICAL
Outcome: BMI											
3	RCT	Serious[1]	No serious	No serious	No serious	64	66	MD: 1.72 kg/m^2 [-0.29, 3.74]	No difference	⊕⊕⊕◯<br>MODERATE	CRITICAL
Outcome: ALT											
1	RCT	Serious[1]	Not applicable	Not applicable	Serious[6]	28	24	MD: 1.10 [-2.84, 5.04]	No difference	⊕⊕◯◯<br>LOW	CRITICAL
Outcome: AST											
1	RCT	Serious[1]	Not applicable	Not applicable	Serious[6]	28	24	MD: -0.60 [-3.27, 2.07]	No difference	⊕⊕◯◯<br>LOW	CRITICAL
Outcome: Hirsutism											
1	RCT	Serious[1]	Not applicable	Not applicable	Serious[6]	72	25	AA + OCP: mean: -57% SD: 2.4%; OCP: mean: -68%, SD: 5.2%	No difference	⊕⊕◯◯<br>LOW	CRITICAL
Outcome: Minor depressive state/mood reduction											
1	RCT	Serious[1]	Not applicable	Not applicable	Serious[6]	28	24	OR: 2.67 [0.10, 68.70]	No difference	⊕⊕◯◯<br>LOW	CRITICAL
Outcome: Dysmenorrhea											
1	RCT	Serious[1]	Not applicable	Not applicable	Serious[6]	28	24	OR: 0.27 [0.01, 7.07]	No difference	⊕⊕◯◯<br>LOW	CRITICAL
Outcome: Acne											
1	RCT	Serious[1]	Not applicable	Not applicable	Serious[6]	72	25	AA + OCP: mean: -78% SD: 13.6%; OCP: mean: -68%, SD: 26%	No difference	⊕⊕◯◯<br>LOW	IMPORTANT
Outcome: Testosterone											
2	RCT	Serious[1]	Serious[3]	No serious	Serious[6]	36	42	MD: 0.10 nmol/l [-0.60, 0.79]	No difference	⊕◯◯◯<br>VERY LOW	IMPORTANT
Outcome: SHBG											
2	RCT	Very serious[1]	Very serious[3,4]	No serious	Very Serious[5,6]	29	30	MD: -38.37 nmol/l[118.45, 41.72]	No difference	⊕◯◯◯<br>VERY LOW	IMPORTANT
Outcome: Total cholesterol											
2	RCT	Serious[1]	No serious	No serious	Serious[6]	48	45	MD: 20.81 mg/dl [7.81, 33.82]	AA + COCP	⊕⊕◯◯<br>LOW	IMPORTANT
Outcome: HDL											
2	RCT	Serious[1]	No serious	No serious	Serious[6]	48	45	MD: 1.44 mg/dl [-5.55, 8.42]	No difference	⊕⊕◯◯<br>LOW	IMPORTANT
Outcome: LDL											

Supplementary File 3

2	RCT	Serious[1]	No serious	No serious	Serious[6]	48	45	MD: 15.12 mg/dl [3.20, 27.04]	AA + COCP	⊕⊕◯◯ LOW	IMPORTANT
Outcome: Triglycerides											
2	RCT	Serious[1]	No serious	No serious	Serious[6]	48	45	MD: 41.34 mg/dl [20.26, 62.42]	AA + COCP	⊕⊕◯◯ LOW	IMPORTANT
Outcome: Breast tenderness											
2	RCT	Serious[1]	Serious[2]	Serious[7]	Serious[6]	100	49	OR: 0.73 [0.11, 5.00]	No difference	⊕◯◯◯ VERY LOW	IMPORTANT
Outcome: Nausea											
2	RCT	Serious[1]	No serious	Serious[7]	Serious[6]	100	49	OR: 0.67 [0.12, 3.63]	No difference	⊕◯◯◯ VERY LOW	IMPORTANT
Fasting insulin											
1	RCT	Serious[1]	Not applicable	Not applicable	Serious[6]	20	21	MD: 3.50 µIU/ml [0.20, 6.80]	AA + COCP	⊕⊕◯◯ LOW	IMPORTANT
Fasting glucose											
1	RCT	Serious[1]	Not applicable	Not applicable	Serious[6]	28	24	MD: 2.60 [-1.74, 6.94]	No difference	⊕⊕◯◯ LOW	IMPORTANT
HOMA-IR											
1	RCT	Serious[1]	Not applicable	Not applicable	Serious[6]	20	21	MD: 0.70 [0.02, 1.38]	AA + COCP	⊕⊕◯◯ LOW	IMPORTANT
Free testosterone											
1	RCT	Very serious[1]	Not applicable	Not applicable	Very serious[6]	9	9	MD: -0.10 pg/ml [-0.47, 0.27]	No difference	⊕◯◯◯ VERY LOW	IMPORTANT
FAI											
1	RCT	Serious[1]	Not applicable	Not applicable	Serious[6]	20	21	MD: 0.50 [0.01, 0.99]	AA + COCP	⊕⊕◯◯ LOW	IMPORTANT
DHEAS											
1	RCT	Very serious[1]	Not applicable	Not applicable	Very serious[6]	9	9	MD: -0.40 µg/ml [-1.23, 0.43]	No difference	⊕◯◯◯ VERY LOW	IMPORTANT
Androstenedione											
1	RCT	Very serious[1]	Not applicable	Not applicable	Very serious[6]	9	9	MD: 0.10 ng/ml [-0.92, 1.12]	No difference	⊕◯◯◯ VERY LOW	IMPORTANT
LDL/HDL ratio											
1	RCT	Serious[1]	Not applicable	Not applicable	Serious[6]	72	25	AA + OCP: mean: -5.1%, SD: 71.3%; OCP: -5.8%, SD: 35.5%	No difference	⊕⊕◯◯ LOW	IMPORTANT
CRP											
1	RCT	Serious[1]	Not applicable	Not applicable	Serious[6]	20	21	MD: 2.40 mg/l [-1.79, 6.59]	No difference	⊕⊕◯◯ LOW	IMPORTANT
Outcome: Acne											
1	RCT	Serious[1]	Not applicable	Not applicable	Serious[6]	72	25	AA + OCP: mean: -78%, SD: 13.6%; OCP: -68%, SD: 26%	No difference	⊕⊕◯◯ LOW	IMPORTANT
Menorrhagia/Heavy menstrual bleeding											
1	RCT	Serious[1]	Not applicable	Not applicable	Serious[6]	28	24	OR: 2.67 [0.10, 68.70]	No difference	⊕⊕◯◯ LOW	IMPORTANT
Premenstrual pelvic pain											

Supplementary File 3

1	RCT	Serious[1]	Not applicable	Not applicable	Serious[6]	72	25	AA + OCP: N events: 0, total: 72; OCP + P: N events: 0, total: 25	No difference	⊕⊕◯◯ LOW	IMPORTANT
Metrorrhagia											
1	RCT	Serious[1]	Not applicable	Not applicable	Serious[6]	28	24	OR: 2.67 [0.10, 68.70]	No difference	⊕⊕◯◯ LOW	IMPORTANT
Hypercholesterolemia											
1	RCT	Serious[1]	Not applicable	Not applicable	Serious[6]	28	24	OR: 1.40 [0.47, 4.20]	No difference	⊕⊕◯◯ LOW	IMPORTANT
Hypertriglyceridemia											
1	RCT	Serious[1]	Not applicable	Not applicable	Serious[6]	28	24	OR: 1.40 [0.47, 4.20]	No difference	⊕⊕◯◯ LOW	IMPORTANT
Menstrual spotting											
1	RCT	Serious[1]	Not applicable	Not applicable	Serious[6]	28	24	OR: 1.67 [0.42, 6.58]	No difference	⊕⊕◯◯ LOW	IMPORTANT

[1] Downgraded as at least one study is moderate RoB or very serious for at least one high RoB
[2] Downgraded due to inconsistent direction of effect
[3] Downgraded due to statistically significant (p<0.05) or high heterogeneity (>50%).
[4] Downgraded due to no overlapping or at least one non-overlapping CI
[5] Downgraded due to wide CIs
[6] Downgraded once due to sample size (and when sample is not similar numbers between intervention and comparison group) and twice for very small sample
[7] Downgraded due to indirectness as not systematic measurement of this outcome in Moretti 2018).

Supplementary File 3

GRADE assessment – Anti-androgen + COCP vs metformin - ADULTS

**Anti-androgen + COCP vs MET – ADULTS (6 months to 12 months)**											
	Quality assessment					No of participants					
**No. studies**	**Design**	**Risk of bias**	**Inconsistency**	**Indirectness**	**Imprecision**	**AA + COCP**	**MET**	**Effect, fixed**	**Favours**	**Certainty**	**Importance**
Outcome: BMI											
2	RCT	Serious[1]	No serious	No serious	No serious	50	57	MD: -0.07 [-1.81, 1.67]	No difference	⊕⊕⊕◯ MODERATE	CRITICAL
Outcome: Hirsutism											
1	RCT	Very serious[1]	Not applicable	Not applicable	Serious[2]	24	22	MD: 4.6 [-2.6, 6.7]	MET	⊕◯◯◯ VERY LOW	CRITICAL
Outcome: Menstrual dysfunction											
1	RCT	Very serious[1]	Not applicable	Not applicable	Serious[2]	24	22	OR: 0.06 [0.02, 0.23]	AA + COCP	⊕◯◯◯ VERY LOW	CRITICAL
Outcome: Fasting glucose											
1	RCT	No serious	Not applicable	Not applicable	Serious[2]	34	34	MD: 0.70 nmol/L [0.32, 1.07]	AA + COCP	⊕⊕◯◯ LOW	IMPORTANT
Outcome: Fasting insulin											
1	RCT	Serious[1]	Not applicable	Not applicable	Very serious[2]	16	23	AA + OCP: median: 2.88 mU/l, IQR: 2.36-3.14; MET: median: 2.37 mU/l, IQR: 2.10-3.23	No difference	⊕◯◯◯ VERY LOW	IMPORTANT
Outcome: HOMA-IR											
1	RCT	Serious[1]	Not applicable	Not applicable	Very serious[2]	16	23	AA + OCP: median: 1.27 IQR: 0.67-1.58; MET: median: 0.77, IQR: 0.40-1.53	No difference	⊕◯◯◯ VERY LOW	IMPORTANT
Outcome: Testosterone											
1	RCT	Serious[1]	Not applicable	Not applicable	Very serious[2]	16	23	MD: 0.01 nmol/l [-0.72, 0.74]	No difference	⊕◯◯◯ VERY LOW	IMPORTANT
Outcome: Free testosterone											
1	RCT	Very serious[1]	Not applicable	Not applicable	Serious[2]	24	22	MD: 0.025 nmol/l [0.012, 0.039]	MET	⊕◯◯◯ VERY LOW	IMPORTANT
Outcome: Androstenedione											
1	RCT	Very serious[1]	Not applicable	Not applicable	Serious[2]	24	22	MD: 5.5 nmol/l [1.8, 9.2]	MET	⊕◯◯◯ VERY LOW	IMPORTANT
Outcome: DHEAS											
1	RCT	Very serious[1]	Not applicable	Not applicable	Serious[2]	24	22	MD: 2.7 µmol/l [1.4, 4.0]	MET	⊕◯◯◯ VERY LOW	IMPORTANT
Outcome: HDL											
1	RCT	No serious	Not applicable	Not applicable	Serious[2]	34	34	MD: -0.00 mmol/l [-0.09, 0.08]	No difference	⊕⊕◯◯ LOW	IMPORTANT
Outcome: Triglycerides											
1	RCT	No serious	Not applicable	Not applicable	Serious[2]	34	34	MD: -0.06 mmol/l [-0.35, 0.23]	No difference	⊕⊕⊕◯ MODERATE	IMPORTANT
Outcome: CRP											

Supplementary File 3

1	RCT	No serious	Not applicable	Not applicable	Serious[2]	34	34	MD: -0.23 mg/l [-0.42, -0.04]	AA + COCP	⊕⊕⊕◯ MODERATE	IMPORTANT
Outcome: Abnormal glucose tolerance											
1	RCT	Very serious[1]	Not applicable	Not applicable	Serious[2]	24	22	OR: 1.7 [0.7, 4.4]	No difference	⊕◯◯◯ VERY LOW	IMPORTANT
Outcome: Dyslipidemia											
1	RCT	Very serious[1]	Not applicable	Not applicable	Serious[2]	24	22	OR: 0.6 [0.2, 1.8]	No difference	⊕◯◯◯ VERY LOW	IMPORTANT

[1] Downgraded once if one or more studies have moderate RoB with significant weighting and very serious for at least one high RoB
[2] Downgraded once for small sample size or twice for very small sample size.

GRADE assessment – Anti-androgen + metformin vs COCP - ADOLESCENTS

**Anti-androgen + MET vs COCP – ADOLESCENTS (9 months) (also in COCP and metformin questions, 4.2, 4.3)**											
	Quality assessment					No of participants					
**No. studies**	**Design**	**Risk of bias**	**Inconsistency**	**Indirectness**	**Imprecision**	**AA + MET**	**COCP**	**Effect, random**	**Favours**	**Certainty**	**Importance**
Outcome: BMI											
1	RCT	Serious[1]	Not applicable	Not applicable	Very serious[2]	16	16	MD: -0.50 kg/m^2 [-2.03, 1.03]	No difference	⊕◯◯◯ VERY LOW	CRITICAL
Outcome: Hirsutism											
1	RCT	Serious[1]	Not applicable	Not applicable	Very serious[2]	16	16	MD: -0.50 [-2.73, 1.73]	No difference	⊕◯◯◯ VERY LOW	CRITICAL
Outcome: Fasting glucose/insulin ratio											
1	RCT	Serious[1]	Not applicable	Not applicable	Very serious[2]	16	16	MD: 2.40 [-0.93, 5.73]	No difference	⊕◯◯◯ VERY LOW	IMPORTANT
Outcome: SHBG											
1	RCT	Serious[1]	Not applicable	Not applicable	Very serious[2]	16	16	MD: -3.40 µg/dl [-4.21, -2.59]	AA + MET	⊕◯◯◯ VERY LOW	IMPORTANT
Outcome: Testosterone											
1	RCT	Serious[1]	Not applicable	Not applicable	Very serious[2]	16	16	MD: -5.00 ng/dl [-23.91, 16.91]	No difference	⊕◯◯◯ VERY LOW	IMPORTANT
Outcome: Triglycerides											
1	RCT	Serious[1]	Not applicable	Not applicable	Very serious[2]	16	16	MD: -44.00 mg/dl [-61.53, -26.47]	AA + MET	⊕◯◯◯ VERY LOW	IMPORTANT
Outcome: HDL											
1	RCT	Serious[1]	Not applicable	Not applicable	Very serious[2]	16	16	MD: -9.00 mg/dl [-17.77, -0.23]	AA + MET	⊕◯◯◯ VERY LOW	IMPORTANT
Outcome: LDL											
1	RCT	Serious[1]	Not applicable	Not applicable	Very serious[2]	16	16	MD: -26.00 mg/dl [-42.86, -9.14]	AA + MET	⊕◯◯◯ VERY LOW	IMPORTANT

[1] Downgraded once as the single study is moderate RoB
[2] Downgraded twice due to very low sample size

Supplementary File 3

GRADE assessment – Anti-androgen + metformin + COCP vs COCP - ADULTS

**Anti-androgen + MET + COCP vs COCP – ADULTS (9 months) (also in metformin and COCP questions, 4.2, 4.3).**											
	Quality assessment					No of participants					
**No. studies**	**Design**	**Risk of bias**	**Inconsistency**	**Indirectness**	**Imprecision**	**AA + MET+ COCP**	**COCP**	**Effect, fixed**	**Favours**	**Certainty**	**Importance**
Outcome: BMI											
1	RCT	Serious[1]	Not applicable	Not applicable	Very serious[2]	11	11	MD: -0.30 kg/m^2 [-2.19, 1.59]	No difference	⨁◯◯◯ VERY LOW	CRITICAL
Outcome: Hirsutism											
1	RCT	Serious[1]	Not applicable	Not applicable	Very serious[2]	11	11	MD: 1.00 [-0.92, 2.92]	No difference	⨁◯◯◯ VERY LOW	CRITICAL
Outcome: Fasting glucose/insulin ratio											
1	RCT	Serious[1]	Not applicable	Not applicable	Very serious[2]	11	11	MD: 0.90 [-0.66, 2.46]	No difference	⨁◯◯◯ VERY LOW	IMPORTANT
Outcome: SHBG											
1	RCT	Serious[1]	Not applicable	Not applicable	Very serious[2]	11	11	MD: 1.10 µg/dl [0.13, 2.07]	COCP	⨁◯◯◯ VERY LOW	IMPORTANT
Outcome: Testosterone											
1	RCT	Serious[1]	Not applicable	Not applicable	Very serious[2]	11	11	MD: 1.00 ng/dl [-17.48, 19.48]	No difference	⨁◯◯◯ VERY LOW	IMPORTANT
Triglycerides											
1	RCT	Serious[1]	Not applicable	Not applicable	Very serious[2]	11	11	MD: -8.00 mg/dl [-47.70, 31.70]	No difference	⨁◯◯◯ VERY LOW	IMPORTANT
Outcome: HDL											
1	RCT	Serious[1]	Not applicable	Not applicable	Very serious[2]	11	11	MD: 0.00 mg/dl [-12.57, 12.57]	No difference	⨁◯◯◯ VERY LOW	IMPORTANT
Outcome: LDL											
1	RCT	Serious[1]	Not applicable	Not applicable	Very serious[2]	11	11	MD: -7.00 mg/dl [-25.06, 11.06]	No difference	⨁◯◯◯ VERY LOW	IMPORTANT

[1] Downgraded once as the single study is moderate RoB
[2] Downgraded twice due to very small sample size/participants

Supplementary File 3

GRADE assessment – Anti-androgen + pioglitazone + metformin (SPIOMET) vs COCP - ADULTS

**Anti-androgen + Pioglitazone + MET (SPIOMET) vs COCP (12 months) – ADOLESCENTS (also in COCP and metformin questions, 4.2, 4.3).**											
	Quality assessment					No of participants					
**No. studies**	**Design**	**Risk of bias**	**Inconsistency**	**Indirectness**	**Imprecision**	**SPIO MET**	**COCP**	**Effect, fixed**	**Favours**	**Certainty**	**Importance**
Outcome: BMI											
1	RCT	Serious[1]	Not applicable	Not applicable	Serious[2]	31	31	MD: -1.00 kg/m^2 [-3.08, 1.08]	No difference	⊕⊕◯◯ LOW	CRITICAL
Outcome: Hirsutism											
1	RCT	Serious[1]	Not applicable	Not applicable	Serious[2]	31	31	MD: -3.00 [-5.77, -0.23]	SPIOMET	⊕⊕◯◯ LOW	CRITICAL
Outcome: ALT											
1	RCT	Serious[1]	Not applicable	Not applicable	Serious[2]	31	31	MD: -0.09 µkat/L [-0.16, -0.02]	SPIOMET	⊕⊕◯◯ LOW	CRITICAL
Outcome: AST											
1	RCT	Serious[1]	Not applicable	Not applicable	Serious[2]	31	31	MD: 0.00 [-0.06, 0.06]	No difference	⊕⊕◯◯ LOW	CRITICAL
Outcome: Waist circumference											
1	RCT	Serious[1]	Not applicable	Not applicable	Serious[2]	31	31	MD: -4.00 cm [-8.38, 0.38]	No difference	⊕⊕◯◯ LOW	IMPORTANT
Outcome: SHBG											
1	RCT	Serious[1]	Not applicable	Not applicable	Serious[2]	31	31	MD: -29.00 nmol/l [-39.56, -18.44]	SPIOMET	⊕⊕◯◯ LOW	IMPORTANT
Outcome: Testosterone											
1	RCT	Serious[1]	Not applicable	Not applicable	Serious[2]	31	31	MD: -0.10 nmol/l [-0.38, 0.18]	No difference	⊕⊕◯◯ LOW	IMPORTANT
Outcome: Androstenedione											
1	RCT	Serious[1]	Not applicable	Not applicable	Serious[2]	31	31	MD: 1.00 nmol/l [0.29,1.71]	SPIOMET	⊕⊕◯◯ LOW	IMPORTANT
Outcome: Fasting insulin											
1	RCT	Serious[1]	Not applicable	Not applicable	Serious[2]	31	31	MD: -62.00 pmol/l [-81.40, -42.60]	SPIOMET	⊕⊕◯◯ LOW	IMPORTANT
Outcome: HOMA-IR											
1	RCT	Serious[1]	Not applicable	Not applicable	Serious[2]	31	31	MD: -1.80 [-2.42, -1.18]	SPIOMET	⊕⊕◯◯ LOW	IMPORTANT
Outcome: Triglycerides											
1	RCT	Serious[1]	Not applicable	Not applicable	Serious[2]	31	31	MD: -0.08 nmol/l [-0.22, 0.06]	No difference	⊕⊕◯◯ LOW	IMPORTANT
Outcome: LDL											
1	RCT	Serious[1]	Not applicable	Not applicable	Serious[2]	31	31	MD: -0.50 mmol/l [-0.78, -0.22]	SPIOMET	⊕⊕◯◯ LOW	IMPORTANT
Outcome: HDL											

Supplementary File 3

1	RCT	Serious[1]	Not applicable	Not applicable	Serious[2]	31	31	MD: 0.10 mmol/l [-0.18. 0.38]	No difference	⊕⊕◯◯ LOW	IMPORTANT
Outcome: CRP											
1	RCT	Serious[1]	Not applicable	Serious[3]	Serious[2]	31	31	MD: -18.10 mmol/l [-25, 75, -10.45]	SPIOMET	⊕⊕◯◯ LOW	IMPORTANT
Outcome: FAI											
1	RCT	Serious[1]	Not applicable	Not applicable	Serious[2]	29	29	MD: -2.20 [-4.38, -0.02]	SPIOMET	⊕⊕◯◯ LOW	IMPORTANT
Outcome: Acne											
1	RCT	Serious[1]	Not applicable	Not applicable	Very serious[2]	18	18	MD: -0.30 [-3.07, 2.46]	No difference	⊕⊕◯◯ LOW	IMPORTANT

[1] Downgraded once as single study is moderate risk of bias
[2] Downgraded once due sample size/participants

Supplementary File 4

Data and analyses

1 Anti-androgen vs Placebo - ADOLESCENTS

Outcome or Subgroup	Studies	Participants	Statistical Method	Effect Estimate
1.1 Body mass index [kg]	1	14	Mean Difference (IV, Random, 95% CI)	0.50 [-3.64, 4.64]
1.2 Hirsutism [FG score]	1	14	Mean Difference (IV, Random, 95% CI)	-4.00 [-6.81, -1.19]
1.3 SHBG [µg/ml]	1	14	Mean Difference (IV, Random, 95% CI)	0.00 [-1.27, 1.27]
1.4 Testosterone [ng/dl]	1	14	Mean Difference (IV, Random, 95% CI)	0.30 [-2.27, 2.87]
1.5 DHEAS [µmol/L]	1	14	Mean Difference (IV, Random, 95% CI)	0.60 [0.07, 1.13]
1.6 Androstenedione [Ng/ml]	1	14	Mean Difference (IV, Random, 95% CI)	-0.10 [-1.58, 1.38]

2 Anti-androgen vs Placebo - ADULTS

Outcome or Subgroup	Studies	Participants	Statistical Method	Effect Estimate
2.1 Body mass index [kg]	1	11	Mean Difference (IV, Random, 95% CI)	-0.80 [-3.64, 2.04]
2.2 Fasting glucose [mg/dL]	1	11	Mean Difference (IV, Random, 95% CI)	3.30 [-2.93, 9.53]
2.3 SHBG [nmol/L]	1	11	Mean Difference (IV, Random, 95% CI)	1.80 [-17.51, 21.11]
2.4 Total testosterone [ng/dl]	1	11	Mean Difference (IV, Random, 95% CI)	12.30 [-22.13, 46.73]
2.5 DHEAS [µg/dL]	1	11	Mean Difference (IV, Random, 95% CI)	-24.50 [-62.22, 13.22]
2.6 Androstenedione [mg/dL]	1	11	Mean Difference (IV, Random, 95% CI)	38.90 [-84.25, 162.05]
2.7 Free testosterone [pg/ml]	1	11	Mean Difference (IV, Random, 95% CI)	0.50 [-2.58, 3.58]
2.8 Dihydrotestosterone [ng/dL]	1	11	Mean Difference (IV, Random, 95% CI)	-0.50 [-8.05, 7.05]
2.9 Fasting insulin [uU/mL]	1	11	Mean Difference (IV, Random, 95% CI)	-3.00 [-6.39, 0.39]
2.10 Insulin sensitivity [/min/uU/mL]	1	11	Mean Difference (IV, Random, 95% CI)	-1.00 [-4.02, 2.02]

3 Anti-androgen + lifestyle vs Placebo + lifestyle

Outcome or Subgroup	Studies	Participants	Statistical Method	Effect Estimate
3.1 BMI [kg/m^2]	2	89	Mean Difference (IV, Random, 95% CI)	-3.08 [-8.67, 2.50]
3.2 Hirsutism [FG score]	2	89	Mean Difference (IV, Random, 95% CI)	-0.93 [-3.37, 1.51]
3.3 SHBG [nmol/l]	2	89	Mean Difference (IV, Random, 95% CI)	9.72 [-0.71, 20.14]
3.4 Weight [kg]	1	36	Mean Difference (IV, Random, 95% CI)	-17.00 [-25.37, -8.63]
3.5 FAI [ng/ml]	1	36	Mean Difference (IV, Random, 95% CI)	-0.80 [-2.34, 0.74]
3.6 DHEAS	1	36	Mean Difference (IV, Random, 95% CI)	-2.34 [-4.06, -0.62]
3.7 Androstenedione	1	36	Mean Difference (IV, Random, 95% CI)	-18.00 [-74.86, 38.86]
3.8 Fasting insulin	1	36	Mean Difference (IV, Random, 95% CI)	-4.00 [-6.98, -1.02]
3.9 HOMA	1	20	Mean Difference (IV, Random, 95% CI)	1.60 [-1.64, 4.84]
3.10 Fasting glucose	1	36	Mean Difference (IV, Random, 95% CI)	0.00 [-5.24, 5.24]

3.11 QUICKI	1	36	Mean Difference (IV, Random, 95% CI)	0.04 [0.02, 0.06]
3.12 Insulin sensitivity index	1	36	Mean Difference (IV, Random, 95% CI)	5.10 [2.32, 7.88]
3.13 HDL	1	36	Mean Difference (IV, Random, 95% CI)	5.00 [-1.54, 11.54]
3.14 LDL	1	36	Mean Difference (IV, Random, 95% CI)	-21.00 [-40.93, -1.07]
3.15 Triglycerides	1	36	Mean Difference (IV, Random, 95% CI)	-50.00 [-77.60, -22.40]
3.16 Frequency of menstruation	1	36	Mean Difference (IV, Random, 95% CI)	-0.80 [-1.54, -0.06]
3.17 Testosterone	1	36	Mean Difference (IV, Random, 95% CI)	0.05 [-0.05, 0.15]

4 AA (daily) vs AA (every 3 days)

Outcome or Subgroup	Studies	Participants	Statistical Method	Effect Estimate
4.1 Hirsutism [FG score]	2	80	Mean Difference (IV, Random, 95% CI)	-3.48 [-4.58, -2.39]
4.2 SHBG [nmol/l]	2	80	Mean Difference (IV, Random, 95% CI)	0.29 [-2.18, 2.76]
4.3 Androstenedione [ng/ml]	2	80	Mean Difference (IV, Random, 95% CI)	-0.30 [-0.50, -0.10]
4.4 Testosterone [ng/ml]	2	80	Mean Difference (IV, Random, 95% CI)	-0.25 [-0.73, 0.23]
4.5 DHEAS [µg/ml]	2	80	Mean Difference (IV, Random, 95% CI)	0.19 [-0.79, 1.17]
4.6 Dry skin	1	64	Odds Ratio (IV, Random, 95% CI)	6.60 [2.21, 19.73]
4.7 Decreased libido	1	64	Odds Ratio (IV, Random, 95% CI)	1.62 [0.41, 6.38]
4.8 Headache	1	64	Odds Ratio (IV, Random, 95% CI)	0.23 [0.02, 2.14]
4.9 Body mass index [kg/m^2]	1	16	Mean Difference (IV, Random, 95% CI)	0.30 [-3.67, 4.27]
4.10 Fasting insulin [µU/ml]	1	64	Mean Difference (IV, Random, 95% CI)	0.70 [-0.17, 1.57]

5 Anti-androgen vs MET - ADULTS

Outcome or Subgroup	Studies	Participants	Statistical Method	Effect Estimate
5.1 BMI	1	35	Mean Difference (IV, Random, 95% CI)	-0.20 [-2.37, 1.97]
5.2 Hirsutism	1	35	Mean Difference (IV, Random, 95% CI)	0.60 [-2.80, 4.00]
5.3 SHBG	1	35	Mean Difference (IV, Random, 95% CI)	11.50 [-0.08, 23.08]
5.4 Free testosterone	1	35	Mean Difference (IV, Random, 95% CI)	-0.30 [-0.85, 0.25]
5.5 DHEAS	1	35	Mean Difference (IV, Random, 95% CI)	-669.00 [-1430.65, 92.65]
5.6 Androstenedione	1	35	Mean Difference (IV, Random, 95% CI)	0.30 [-0.13, 0.73]
5.7 HOMA-IR	1	35	Mean Difference (IV, Random, 95% CI)	-0.20 [-0.88, 0.48]

6 Anti-androgen vs Anti-androgen + MET - ADULTS

Outcome or Subgroup	Studies	Participants	Statistical Method	Effect Estimate
6.1 BMI	1	33	Mean Difference (IV, Random, 95% CI)	0.10 [-2.25, 2.45]
6.2 Hirsutism	1	33	Mean Difference (IV, Random, 95% CI)	-0.40 [-4.05, 3.25]
6.3 SHBG	1	33	Mean Difference (IV, Random, 95% CI)	-1.00 [-14.72, 12.72]

6.4 Free testosterone	1	33	Mean Difference (IV, Random, 95% CI)	0.10 [-0.52, 0.72]
6.5 DHEAS	1	33	Mean Difference (IV, Random, 95% CI)	-198.00 [-941.98, 545.98]
6.6 Androstenedione	1	33	Mean Difference (IV, Random, 95% CI)	0.30 [-0.14, 0.74]
6.7 HOMA-IR	1	33	Mean Difference (IV, Random, 95% CI)	-0.40 [-1.07, 0.27]

7 Anti-androgen + lifestyle vs MET + lifestyle - ADULTS

Outcome or Subgroup	Studies	Participants	Statistical Method	Effect Estimate
7.1 Weight [kg]	2	144	Mean Difference (IV, Random, 95% CI)	-5.64 [-19.33, 8.05]
7.2 Body mass index [kg/m^2]	4	265	Mean Difference (IV, Random, 95% CI)	-0.79 [-2.45, 0.87]
7.3 WHR [cm]	2	176	Mean Difference (IV, Random, 95% CI)	0.02 [-0.00, 0.04]
7.4 Frequency of menstruation [cycles/y]	2	176	Mean Difference (IV, Random, 95% CI)	0.79 [0.05, 1.53]
7.5 Hirsutism [FG score]	4	265	Mean Difference (IV, Random, 95% CI)	-1.59 [-3.06, -0.12]
7.6 SHBG [nmol/l]	2	89	Mean Difference (IV, Random, 95% CI)	7.70 [0.75, 14.66]
7.7 Testosterone [nmol/l]	3	213	Mean Difference (IV, Random, 95% CI)	0.03 [-0.21, 0.27]
7.8 DHEAS [µmol/l]	2	106	Mean Difference (IV, Random, 95% CI)	-1.02 [-3.27, 1.24]
7.9 Fasting insulin [µU/ml]	4	213	Mean Difference (IV, Random, 95% CI)	-2.11 [-3.97, -0.26]
7.10 Fasting glucose [mg/dl]	3	228	Mean Difference (IV, Random, 95% CI)	0.44 [-2.14, 3.02]
7.11 Fasting glucose-insulin ratio [mg/dl]	2	176	Mean Difference (IV, Random, 95% CI)	-1.12 [-1.44, -0.79]
7.12 QUICKI [mg/dl]	2	144	Mean Difference (IV, Random, 95% CI)	0.01 [-0.02, 0.04]
7.13 FAI	1	37	Mean Difference (IV, Random, 95% CI)	-0.60 [-1.99, 0.79]
7.14 Androstenedione	1	37	Mean Difference (IV, Random, 95% CI)	-39.00 [-123.43, 45.43]
7.15 OGTT	1	52	Mean Difference (IV, Random, 95% CI)	-9.54 [-23.70, 4.62]
7.16 HOMA	1	69	Mean Difference (IV, Random, 95% CI)	2.72 [-0.28, 5.72]
7.17 Total cholesterol	1	52	Mean Difference (IV, Random, 95% CI)	7.20 [-13.06, 27.46]
7.18 HDL	1	37	Mean Difference (IV, Random, 95% CI)	0.21 [0.05, 0.37]
7.19 LDL	1	37	Mean Difference (IV, Random, 95% CI)	-0.29 [-0.83, 0.25]
7.20 Triglycerides	1	37	Mean Difference (IV, Random, 95% CI)	-0.23 [-0.51, 0.05]

8 Anti-androgen + MET + lifestyle vs MET + lifestyle - ADULTS

Outcome or Subgroup	Studies	Participants	Statistical Method	Effect Estimate
8.1 Weight [kg]	3	210	Mean Difference (IV, Random, 95% CI)	-0.92 [-7.30, 5.45]
8.2 Body mass index [kg/m^2]	4	262	Mean Difference (IV, Random, 95% CI)	-0.20 [-1.43, 1.02]
8.3 WHR [cm]	2	170	Mean Difference (IV, Random, 95% CI)	-0.00 [-0.06, 0.06]
8.4 Hirsutism [FG score]	4	262	Mean Difference (IV, Random, 95% CI)	-0.58 [-1.86, 0.69]
8.5 SHBG [nmol/l]	3	144	Mean Difference (IV, Random, 95% CI)	-0.59 [-4.53, 3.34]

8.6 Testosterone [nmol/l]	4	262	Mean Difference (IV, Random, 95% CI)	-0.29 [-0.52, -0.06]
8.7 DHEAS [µg/ml]	2	92	Mean Difference (IV, Random, 95% CI)	-0.24 [-0.92, 0.45]
8.8 Fasting insulin [µU/ml]	3	210	Mean Difference (IV, Random, 95% CI)	-1.22 [-2.87, 0.43]
8.9 Fasting glucose [mg/dl]	4	262	Mean Difference (IV, Random, 95% CI)	-2.93 [-5.78, -0.09]
8.10 HDL [mg/dl]	2	92	Mean Difference (IV, Random, 95% CI)	1.75 [-7.72, 11.23]
8.11 LDL [mg/dl]	2	92	Mean Difference (IV, Random, 95% CI)	-8.24 [-20.34, 3.86]
8.12 Triglycerides [mg/dl]	2	92	Mean Difference (IV, Random, 95% CI)	-5.48 [-24.06, 13.10]
8.13 HOMA-IR	2	170	Mean Difference (IV, Random, 95% CI)	-0.22 [-0.67, 0.23]
8.14 Frequency of menstruation	1	118	Mean Difference (IV, Random, 95% CI)	0.84 [-0.31, 1.99]
8.15 FAI	1	52	Mean Difference (IV, Random, 95% CI)	-2.10 [-4.47, 0.27]
8.16 Androstenedione	1	40	Mean Difference (IV, Random, 95% CI)	-5.00 [-96.42, 86.42]
8.17 Fasting glucose-insulin ratio	1	118	Mean Difference (IV, Random, 95% CI)	1.74 [-1.45, 4.93]
8.18 QUICKI	1	40	Mean Difference (IV, Random, 95% CI)	0.01 [-0.01, 0.03]
8.19 OGTT	1	52	Mean Difference (IV, Random, 95% CI)	-4.88 [-20.32, 10.56]
8.20 Insulin sensitivity index	1	40	Mean Difference (IV, Random, 95% CI)	6.90 [2.96, 10.84]
8.21 Total cholesterol	1	52	Mean Difference (IV, Random, 95% CI)	0.30 [-16.76, 17.36]

9 Anti-androgen + lifestyle vs Anti-androgen + Metformin + lifestyle

Outcome or Subgroup	Studies	Participants	Statistical Method	Effect Estimate
9.1 BMI [kg/m^2]	3	204	Mean Difference (IV, Random, 95% CI)	-0.05 [-1.21, 1.11]
9.2 Hirsutism [FG score]	3	204	Mean Difference (IV, Random, 95% CI)	-0.84 [-2.71, 1.03]
9.3 SHBG [nmol/l]	2	91	Mean Difference (IV, Random, 95% CI)	8.88 [-7.01, 24.78]
9.4 Testosterone [nmol/l]	3	204	Mean Difference (IV, Random, 95% CI)	0.05 [-0.15, 0.24]
9.5 Fasting insulin [µIU/ml]	2	150	Mean Difference (IV, Random, 95% CI)	-0.18 [-1.94, 1.59]
9.6 Fasting glucose [mg/dl]	3	204	Mean Difference (IV, Random, 95% CI)	3.81 [1.35, 6.28]
9.7 QUICKI	2	150	Mean Difference (IV, Random, 95% CI)	0.00 [-0.03, 0.03]
9.8 Weight	1	113	Mean Difference (IV, Random, 95% CI)	1.11 [-1.97, 4.19]
9.9 WHR	1	113	Mean Difference (IV, Random, 95% CI)	0.05 [0.03, 0.07]
9.10 Total cholesterol	1	54	Mean Difference (IV, Random, 95% CI)	-2.24 [-26.00, 21.52]
9.11 FAI	1	37	Mean Difference (IV, Random, 95% CI)	0.50 [-0.57, 1.57]
9.12 DHEAS	1	37	Mean Difference (IV, Random, 95% CI)	-0.20 [-0.82, 0.42]
9.13 Androstenedione	1	37	Mean Difference (IV, Random, 95% CI)	-34.00 [-98.19, 30.19]
9.14 Triglycerides	1	37	Mean Difference (IV, Random, 95% CI)	-19.00 [-38.11, 0.11]
9.15 HDL	1	37	Mean Difference (IV, Random, 95% CI)	1.00 [-7.59, 9.59]

9.16 HOMA-IR	1	113	Mean Difference (IV, Random, 95% CI)	0.60 [-0.04, 1.24]

10 Anti-androgen vs COCP - ADULTS

Outcome or Subgroup	Studies	Participants	Statistical Method	Effect Estimate
10.1 Hirsutism	1	19	Mean Difference (IV, Random, 95% CI)	-4.00 [-4.90, -3.10]

11 Anti-androgen + COCP vs COCP +/- placebo - ADULTS

Outcome or Subgroup	Studies	Participants	Statistical Method	Effect Estimate
11.1 Weight [kg]	2	93	Mean Difference (IV, Random, 95% CI)	5.57 [-0.67, 11.81]
11.2 Body mass index [kg/m^2]	3	130	Mean Difference (IV, Random, 95% CI)	1.72 [-0.29, 3.74]
11.3 Testosterone [nmol/l]	2	78	Mean Difference (IV, Random, 95% CI)	0.10 [-0.60, 0.79]
11.4 SHBG [nmol/l]	2	59	Mean Difference (IV, Random, 95% CI)	-38.37 [-118.45, 41.72]
11.5 Total cholesterol [mg/dl]	2	93	Mean Difference (IV, Random, 95% CI)	20.81 [7.81, 33.82]
11.6 HDL [mg/dl]	2	93	Mean Difference (IV, Random, 95% CI)	1.44 [-5.55, 8.42]
11.7 LDL [mg/dl]	2	93	Mean Difference (IV, Random, 95% CI)	15.12 [3.20, 27.04]
11.8 Triglycerides [mg/dl]	2	93	Mean Difference (IV, Random, 95% CI)	41.34 [20.26, 62.42]
11.9 Breast tenderness	2	149	Odds Ratio (IV, Random, 95% CI)	0.73 [0.11, 5.00]
11.10 Nausea	2	149	Odds Ratio (IV, Random, 95% CI)	0.67 [0.12, 3.63]
11.11 Fasting insulin	1	41	Mean Difference (IV, Random, 95% CI)	3.50 [0.20, 6.80]
11.12 Fasting glucose	1	52	Mean Difference (IV, Random, 95% CI)	2.60 [-1.74, 6.94]
11.13 HOMA-IR	1	41	Mean Difference (IV, Random, 95% CI)	0.70 [0.02, 1.38]
11.14 FAI	1	41	Mean Difference (IV, Random, 95% CI)	0.50 [0.01, 0.99]

12 Anti-androgen + COCP vs MET - ADULTS

Outcome or Subgroup	Studies	Participants	Statistical Method	Effect Estimate
12.1 Body mass index [kg/m^2]	2	107	Mean Difference (IV, Random, 95% CI)	-0.07 [-1.81, 1.67]
12.2 HDL	1	68	Mean Difference (IV, Random, 95% CI)	-0.00 [-0.09, 0.08]
12.3 fasting glucose	1	68	Mean Difference (IV, Random, 95% CI)	0.70 [0.32, 1.07]
12.4 testosterone	1	39	Mean Difference (IV, Random, 95% CI)	0.01 [-0.72, 0.74]
12.5 Triglycerides	1	68	Mean Difference (IV, Random, 95% CI)	-0.06 [-0.35, 0.23]
12.6 CRP	1	68	Mean Difference (IV, Random, 95% CI)	-0.23 [-0.42, -0.04]

Supplementary File 4

13 Anti-androgen + MET vs COCP - ADOLESCENTS

Outcome or Subgroup	Studies	Participants	Statistical Method	Effect Estimate
13.1 BMI	1	32	Mean Difference (IV, Random, 95% CI)	-0.50 [-2.03, 1.03]
13.2 Hirsutism	1	32	Mean Difference (IV, Random, 95% CI)	-0.50 [-2.73, 1.73]
13.3 Fasting glucose/insulin ratio	1	32	Mean Difference (IV, Random, 95% CI)	2.40 [-0.93, 5.73]
13.4 SHBG	1	32	Mean Difference (IV, Random, 95% CI)	-3.40 [-4.21, -2.59]
13.5 Testosterone	1	32	Mean Difference (IV, Random, 95% CI)	-5.00 [-26.91, 16.91]
13.6 Triglycerides	1	32	Mean Difference (IV, Random, 95% CI)	-44.00 [-61.53, -26.47]
13.7 HDL	1	32	Mean Difference (IV, Random, 95% CI)	-9.00 [-17.77, -0.23]
13.8 LDL	1	32	Mean Difference (IV, Random, 95% CI)	-26.00 [-42.86, -9.14]

14 Anti-androgen + MET + COCP vs COCP - ADULTS

Outcome or Subgroup	Studies	Participants	Statistical Method	Effect Estimate
14.1 BMI	1	22	Mean Difference (IV, Random, 95% CI)	-0.30 [-2.19, 1.59]
14.2 Hirsutism	1	22	Mean Difference (IV, Random, 95% CI)	1.00 [-0.92, 2.92]
14.3 Fasting glucose/insulin ratio	1	22	Mean Difference (IV, Random, 95% CI)	0.90 [-0.66, 2.46]
14.4 SHBG	1	22	Mean Difference (IV, Random, 95% CI)	1.10 [0.13, 2.07]
14.5 Testosterone	1	22	Mean Difference (IV, Random, 95% CI)	1.00 [-17.48, 19.48]
14.6 Triglycerides	1	22	Mean Difference (IV, Random, 95% CI)	-8.00 [-47.70, 31.70]
14.7 HDL	1	22	Mean Difference (IV, Random, 95% CI)	0.00 [-12.57, 12.57]
14.8 LDL	1	22	Mean Difference (IV, Random, 95% CI)	-7.00 [-25.06, 11.06]

16 SPIOMET vs COCP

Outcome or Subgroup	Studies	Participants	Statistical Method	Effect Estimate
16.1 BMI	1	62	Mean Difference (IV, Random, 95% CI)	-1.00 [-3.08, 1.08]
16.2 Waist circumference	1	62	Mean Difference (IV, Random, 95% CI)	-4.00 [-8.38, 0.38]

16.3 Hirsutism	1	62	Mean Difference (IV, Random, 95% CI)	-3.00 [-5.77, -0.23]
16.4 SHBG	1	62	Mean Difference (IV, Random, 95% CI)	-29.00 [-39.56, -18.44]
16.5 Testosterone	1	62	Mean Difference (IV, Random, 95% CI)	-0.10 [-0.38, 0.18]
16.6 Androstenedione	1	62	Mean Difference (IV, Random, 95% CI)	1.00 [0.29, 1.71]
16.7 Fasting insulin	1	62	Mean Difference (IV, Random, 95% CI)	-62.00 [-81.40, -42.60]
16.8 Acne	1	36	Mean Difference (IV, Random, 95% CI)	-0.30 [-3.07, 2.47]
16.9 HOMA-IR	1	62	Mean Difference (IV, Random, 95% CI)	-1.80 [-2.42, -1.18]
16.10 Triglycerides	1	62	Mean Difference (IV, Random, 95% CI)	-0.08 [-0.22, 0.06]
16.11 LDL	1	62	Mean Difference (IV, Random, 95% CI)	-0.50 [-0.78, -0.22]
16.12 HDL	1	62	Mean Difference (IV, Random, 95% CI)	0.10 [-0.18, 0.38]
16.13 CRP	1	62	Mean Difference (IV, Random, 95% CI)	-18.10 [-25.75, -10.45]
16.14 ALT	1	62	Mean Difference (IV, Random, 95% CI)	-0.09 [-0.16, -0.02]
16.15 FAI	1	58	Mean Difference (IV, Random, 95% CI)	-2.20 [-4.38, -0.02]
16.16 AST	1	62	Mean Difference (IV, Random, 95% CI)	0.00 [-0.06, 0.06]
